# Short-range Fgf signalling patterns hindbrain progenitors to induce the neurogenesis-to-oligodendrogenesis switch

**DOI:** 10.1242/dev.204256

**Published:** 2024-12-13

**Authors:** Tim J. Yeung, David G. Wilkinson

**Affiliations:** The Francis Crick Institute, 1 Midland Road, London NW1 1AT, UK

**Keywords:** Fgf signalling, Neurogenesis, Gliogenesis, Hindbrain development, Patterning, Zebrafish

## Abstract

In the vertebrate nervous system, neurogenesis generally precedes gliogenesis. The mechanisms driving the switch in cell type production and generation of the correct proportion of cell types remain unclear. Here, we show that Fgf20 signalling patterns progenitors to induce the switch from neurogenesis to oligodendrogenesis in the zebrafish hindbrain. Fgf20 emanating from earlier-born neurons signals at a short range to downregulate proneural gene expression in the segment centre with high spatial precision along both anterior-posterior and dorsal-ventral axes. This signal induces oligodendrocytes in the segment centre by upregulating *olig2* and *sox10* expression in pre-patterned competent progenitors. We show that the magnitude of proneural gene downregulation and the quantity of oligodendrocyte precursor cells specified is dependent on the extent of Fgf20 signalling. Overexpression of *fgf20a* induces precocious specification and differentiation of oligodendrocytes among *olig2*^+^ progenitors, resulting in an increase in oligodendrocytes at the expense of neurogenesis. Thus, Fgf20 signalling defines the proportion of each cell type produced. Taken together, Fgf20 signalling from earlier-born neurons patterns hindbrain segments spatially and temporally to induce the neurogenesis-to-oligodendrogenesis switch.

## Teaser

**Summary:** Fgf20-expressing neuronal clusters act as short-range signalling centres that pattern proneural gene expression within hindbrain segments and induce glial fates in the segment centre.

## INTRODUCTION

The vertebrate nervous system is characterised by its structural complexity that underlies its remarkable emergent properties. Unlike many other organs that comprise repeating tissue units with a limited number of cell types, the nervous system has an exceptionally large number of distinct cell types relative to its size (Tasic et al., 2018). Mechanisms that underlie increasingly refined tissue patterning over a prolonged period are particularly crucial to neural development.

Strategies for increasing heterogeneity among progenitor cells in the developing nervous system involve spatial and temporal regulation of transcription factor expression together with progenitor maintenance. Extracellular signalling molecules, such as morphogens, play an important role in the spatial patterning of the neural tube along both the anterior-posterior (AP) and the dorsal-ventral (DV) axes (Jessell, 2000; Papalopulu and Kintner, 1996; Sasai et al., 2014). The acquisition of cell type identities is dependent on the concentration and/or duration of morphogen signals (Tozer et al., 2013;Greenfeld et al., 2021). A classic example is the DV-patterning of the ventral neural tube by a concentration gradient of Sonic hedgehog (Shh) signalling (Dessaud et al., 2007), which results in the activation of a robust gene regulatory network and subdivision of the progenitor population into precise proportions (Exelby et al., 2021).

The superimposition of spatial and temporal patterning is required for the specification of later-born cell types. This is particularly relevant to gliogenesis, which generally occurs later in development, (Sagner, 2024) and is preceded by neurogenesis (Cepko, 2014; Guillemot, 2007; Miller and Gauthier, 2007; Rossi et al., 2017; Rowitch and Kriegstein, 2010). Extracellular signalling plays an important role in modulating intrinsic temporal programmes among neural progenitors for the timely production of different cell types (Dias et al., 2014; Rossi and Desplan, 2020; Sagner et al., 2021; Shi and Liu, 2011). Notably, it has been found that earlier-born neurons can act as a source of signalling to regulate the production of later-born cell types (Barnabé-Heider et al., 2005; Seuntjens et al., 2009). Such feedback regulatory mechanisms effectively provide temporal checkpoints in tissue development and ensure spatial precision in cell fate acquisition.

Temporal patterning is necessarily concomitant with progenitor maintenance to produce the correct proportion of late-born cell types. The inhibition of neurogenesis is an essential prerequisite that permits gliogenesis, as proneural basic helix-loop-helix (bHLH) transcription factors repress gliogenesis in neural progenitors (Sun et al., 2001; Zhao et al., 2015). Proneural bHLH factors also commit progenitors to neuronal differentiation (Bertrand et al., 2002; Guillemot and Hassan, 2017), which depletes the progenitor pool for later glial production. For example, the pMN progenitors in the spinal cord generate both somatic motor neurons and oligodendrocytes; lowering the activity of proneural neurogenin 2 by upregulation of *Olig2* helps maintain pMN progenitors for later oligodendrogenesis (Lee et al., 2005). Similarly, gliogenic transcription factors, such as Sox9 and Nfi, exert their function at least partly through inhibiting neurogenesis (Deneen et al., 2006; Stolt et al., 2003; Vong et al., 2015). Juxtacrine Notch signalling is a key pathway implicated in gliogenesis, which inhibits proneural genes through its downstream effectors the HES/Her transcription factors (Itoh et al., 2003; Kageyama and Ohtsuka, 1999). Notch signalling is often permissive rather than instructive for the acquisition of glial fate by inhibiting neurogenesis (Rowitch and Kriegstein, 2010). For example, forced activation of the Notch pathway in the ventral spinal cord results in an overall increase in oligodendrocytes but has a negligible effect on *olig2* expression or in advancing the timing of oligodendrocyte production (Park and Appel, 2003).

Previous studies have identified a role of Fgf20 signalling in patterning proneural gene expression in the hindbrain (Gonzalez-Quevedo et al., 2010; Terriente et al., 2012). Neural development in the hindbrain provides an accessible context for discovering patterning mechanisms required for increasing tissue complexity, as it shares many of the initial conditions of DV-patterning in its posterior counterpart at the spinal cord level (Krumlauf and Wilkinson, 2021). In the zebrafish hindbrain, proneural gene expression is patterned along the AP axis, with a high level of expression flanking the hindbrain boundaries and inhibition in each segment centre (Amoyel et al., 2005). The inhibition of proneural gene expression at the hindbrain boundaries is regulated by Notch signalling as well as Yap/Taz-TEAD-mediated mechanotransduction (Cheng et al., 2004; Voltes et al., 2019), while the inhibition in the segment centre is regulated by Fgf20a signalling emanating from a subset of earlier-born neurons positioned at each centre (Gonzalez-Quevedo et al., 2010). Accompanying the patterning of proneural gene expression, Fgf20 is also responsible for the upregulation of various markers in the segment centre, including gliogenic factors, such as *sox9* and members of the meteorin family (Gonzalez-Quevedo et al., 2010; Lee et al., 2010; Nishino et al., 2004; Stolt et al., 2003; Tambalo et al., 2020). Additionally, Fgf signalling has been implicated in the production of oligodendrocytes (Chandran et al., 2003; Esain et al., 2010; Kessaris et al., 2004). In particular, Esain et al. (2010) found that Fgf-receptor (Fgfr) activation is required for the production of oligodendrocyte precursor cells (OPCs) in the zebrafish hindbrain through its regulation of *olig2* and *sox9* expression. Crucially, the time during which Fgfr activation is required for this process significantly overlaps with the period of Fgf20 signalling (Gonzalez-Quevedo et al., 2010). This suggests a potential role of Fgf20 in mediating the switch from neurogenesis to gliogenesis.

Here, we find that Fgf20 signalling emanating from earlier-born neuronal clusters acts at short range to confer precise spatial and temporal patterning of the hindbrain segments to induce the neurogenesis-to-oligodendrogenesis switch. This signal inhibits neurogenesis by downregulating proneural gene expression and induces oligodendrocytes among competent progenitors in the segment centre. The short-range property of Fgf20 confers precise spatial patterning of multiple proneural genes along both the AP and DV axes. Additionally, we find that the downregulation of early proneural genes is highly sensitive to Fgf pathway activation. Thus, the propensity for neurogenesis among progenitors in the segment centre is defined by the dynamics of Fgf20 signalling. Fgf20 signalling acts instructively as it upregulates specification and differentiation factors of oligodendrocytes among competent cells in the segment centre, and the timing and extent of signalling determines the timing of induction and quantity of oligodendrocytes.

## RESULTS

### Short-range Fgf20 signalling activity in the hindbrain

It was previously hypothesised that *fgf20a*-expressing neuronal clusters establish a morphogen gradient from the segment centre, and that a high level of signalling in the progenitors is required for the inhibition of neurogenesis (Terriente et al., 2012). A correlation between the positioning of the *fgf20a-*expressing neuronal clusters and a patterned Fgfr pathway response in the neuroepithelium has been shown (Gonzalez-Quevedo et al., 2010). However, the methodologies used in these studies lack the spatial resolution necessary to test whether a graded response to Fgf20 is established for the neurogenesis inhibition. To address this, we set out to characterise the expression of *fgf20* and the downstream target genes, *etv5b* and *spry1* (Gonzalez-Quevedo et al., 2010; Raible and Brand, 2001; Roehl and Nüsslein-Volhard, 2001), using hybridisation chain reaction (HCR) RNA fluorescence *in situ* hybridization (RNA-FISH), which enables quantitative measurement of gene expression with high spatial resolution (Choi et al., 2010; Trivedi et al., 2018).

**Fig. 1.**
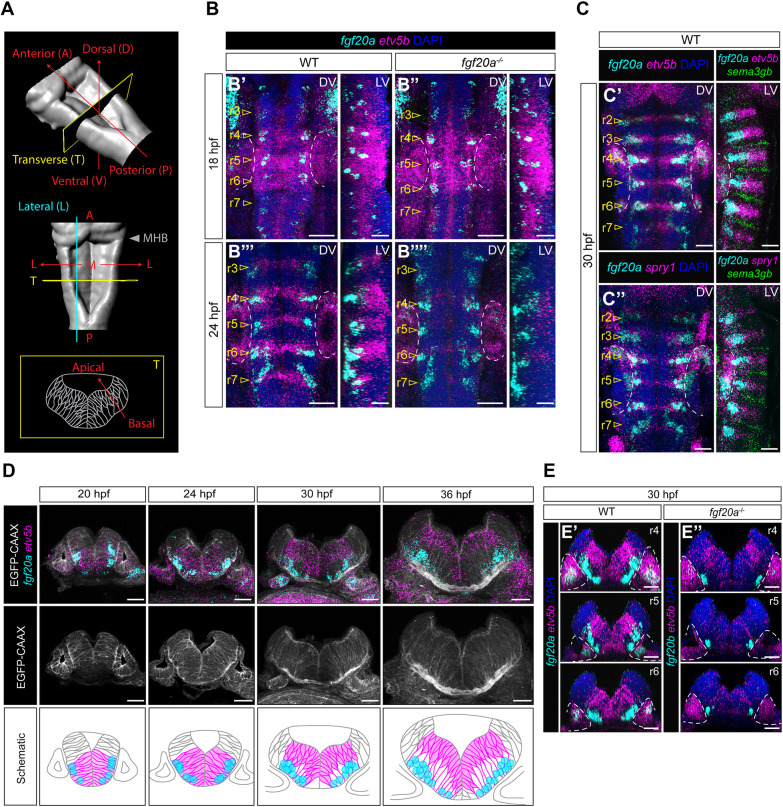
**Short-range Fgf20 signalling.** (A) 3D surface rendering of the hindbrain at 30 hpf based on DAPI staining; axes and planes of the tissue are indicated. M, medial; MHB, midbrain-hindbrain boundary. (B) HCR RNA-FISH for *fgf20a* (cyan) and *etv5b* (magenta) in WT and *fgf20a*^−*/*−^ at 18 hpf and 24 hpf. DAPI (blue). DV, dorsal view; LV, lateral view. *n*=8 (WT 18 hpf, B′); *n*=8 (*fgf20a*^−*/*−^ 18 hpf, B″); *n*=12 (WT 24 hpf, B‴); *n*=12 (*fgf20a*^−*/*−^ 24 hpf B⁗). (C) HCR RNA-FISH for *fgf20a* (cyan), *etv5b* (C′, magenta), *spry1* (C″, magenta) and boundary marker *sema3gb* (green) in WT embryo at 30 hpf. DAPI (blue). Arrowheads (yellow) indicate segment centre. The extent of Fgf20 signalling along the AP axis, as indicated by *etv5b* and *spry1* readouts, is highly restricted. *n*=8 (C′, DV); *n*=10 (C″, DV); *n*=6 (C′, LV); *n*=4 (C″, LV). (D) Transverse sections of hindbrain segment centre of r5 in WT embryo, representative of other hindbrain segments, at stages 20-42 hpf with HCR RNA-FISH for *fgf20a* (cyan), *etv5b* (magenta) and membrane markers EGFP-CAAX (white). Progenitors expressing *etv5b* are aligned with the position of *fgf20a-*expressing neurons, where the basal processes of these progenitors are adjacent to the signalling source. *n*≥8 per stage. (E) Transverse views of hindbrain segment centre in r4-6 with HCR RNA-FISH for *fgf20a* (cyan, E′, WT), *fgf20b* (cyan, E″, *fgf20a*^−*/*−^) and *etv5b* (magenta) at 30 hpf. DAPI (blue). As exemplified by the expression pattern in r5 and r6 of *fgf20a*^−*/*−^ (E″), the remaining low level of *etv5b* in *fgf20a*^−*/*−^ corresponds to Fgf20b signalling. *n*=12 per genotype. Dotted lines demarcate the expression in the otic vesicles. Scale bars: 30 μm.

*Fgf20a-*expressing neurons were first detected in the hindbrain segment centre at 16 h post-fertilisation (hpf), with two or three neurons per segment (Fig. S1A,B). The size of the *fgf20a*-expressing neuronal clusters increased rapidly in the hindbrain from 15-16 hpf onwards (Fig. 1A-C; Fig. S1C). Transverse sections of the segment centre showed that, from 20 hpf onwards, *fgf20a-*expressing neurons were positioned at a ventral-lateral region of the neural tube (Fig. 1D; Fig. S1B). This is concomitant with the morphological changes of the hindbrain during ventricle formation, which causes the lateralisation of the dorsal neural tube and the ventralisation of the mantle zone in which differentiated neurons reside (Elsen et al., 2008; Lowery and Sive, 2005). We found that the orthologue *fgf20b* was also expressed in the hindbrain segment centre (Fig. S2A) and was co-expressed with *fgf20a* in one or two neurons within each of the *fgf20a-*expressing neuronal clusters (hereafter referred to as *fgf20*-expressing neurons) (Fig. S2B). Nonetheless, the severity of *fgf20a*^−*/*−^ embryo defects in the patterning of proneural gene expression suggests that Fgf20a has a major role that is not compensated by Fgf20b (Gonzalez-Quevedo et al., 2010).

Despite detection of *fgf20-*expressing neurons from 16 hpf, *etv5b* expression was initially broad and only became restricted and upregulated in the segment centre from 20 hpf onwards (Fig. 1B,C; Fig. S1A). Before this stage, *etv5b* was expressed broadly in rhombomeres (r) 4-6 due to earlier segmental Fgf3/8a signalling (Maves et al., 2002; Walshe et al., 2002; Wiellette and Sive, 2004). Indeed, it was previously shown that suppression of segmental Fgf3 expression is necessary before the onset of Fgf20-signalling activity for the switch in Fgf signalling patterns (Leino et al., 2023). Importantly, analysis of *etv5b* and *spry1* expression revealed that the extent of Fgf signalling in response to Fgf20 was highly restricted along both the AP and DV axes of the segments. In dorsal and lateral views it was seen that the AP extent of *etv5b* (Fig. 1C′) and *spry1* (Fig. 1C″) expression in the neural epithelium aligned with *fgf20* expression in the mantle zone. We further analysed expression in transverse sections of the hindbrain neural tube in which the DV axis of the neuroepithelium was revealed. The membrane marker EGFP-CAAX was used to visualise the orientations of the neural progenitors (Fig. 1D). In the neural tube, the neuroepithelial cells were arranged perpendicular to the mantle zone, with close contacts between their basal processes and differentiated neurons. We found that *etv5b* expression was restricted to the subpopulation of progenitors that are perpendicular to the *fgf20-*expressing neurons. This suggests that Fgf signalling is activated only in progenitors that are highly proximal to the source of Fgf20. Therefore, Fgf20 signalling may act at short range around the basal region of the neuroepithelium. Indeed, in *efnB3b*^−*/*−^ embryos that have mispositioned *fgf20-*expressing neurons (Cayuso et al., 2019; Terriente et al., 2012), we observed a corresponding shift in *etv5b* expression along the DV axis to the position of *fgf20-*expressing neurons (Fig. S3). Similarly, in *fgf20a*^−*/*−^ embryos, a low level of *etv5b* expression correlated with the position of *fgf20b-*expressing neurons, except for r4, where dorsal *etv5b* expression was adjacent to Fgf3/8a signalling from the anterior pole of the otic vesicle (Figs 1E, 4G; Fig. S2C) (Maier and Whitfield, 2014). Together, these data highlight the limited range of Fgf20 signalling in the zebrafish hindbrain.

A previous study (Kalinina et al., 2009) has shown that some members of the mammalian FGF9/16/20 family, including FGF20, have strong equilibrium bias towards homodimerisation. This acts as an autoinhibitory control of their activity due to occlusion of receptor binding sites in the homodimer, with an increase in the ligand binding affinity to heparin sulphate, which significantly restricts the diffusion range (Harada et al., 2009; Kalinina et al., 2009). Protein sequence alignment of FGF20 and the zebrafish orthologues showed that the residues required for homodimerisation are conserved (Fig. S4). This suggests that the highly restricted signalling range of Fgf20 in the hindbrain could be due to biochemical properties of the ligand.

### Short-range Fgf20 signalling patterns proneural gene expression along the AP and DV axes

**Fig. 2.**
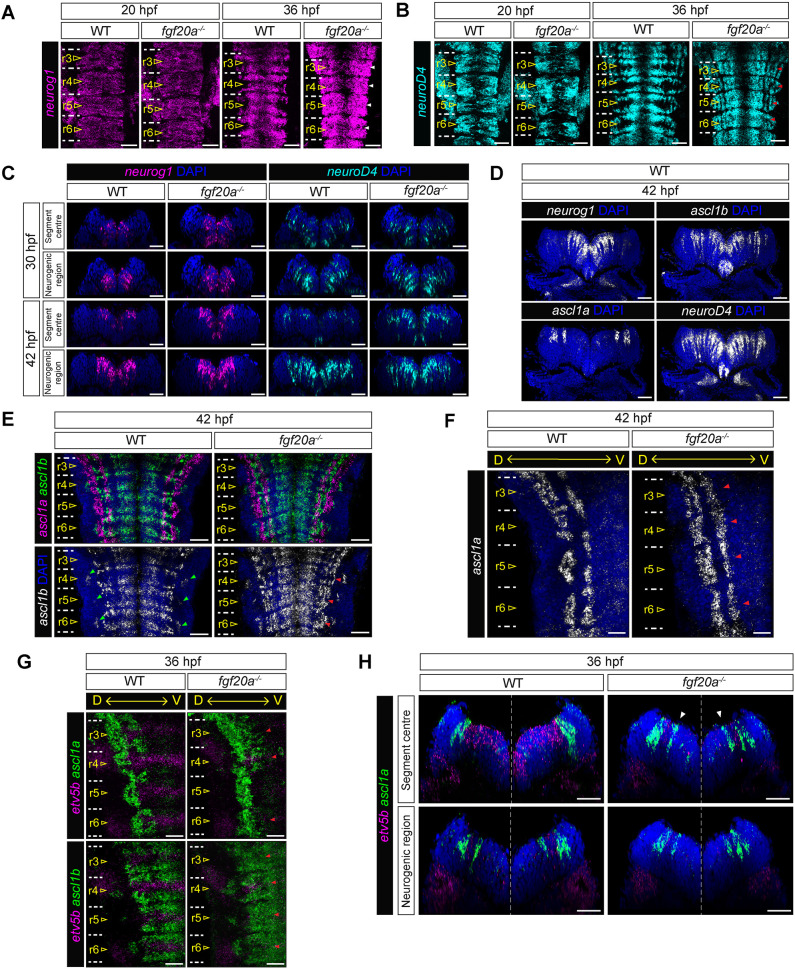
**Fgf20 signalling patterns proneural gene expression along both AP and DV axes.** (A) *Neurog1* in WT and *fgf20a*^−*/*−^ embryos at 20 hpf and 36 hpf. Dorsal view. *Neurog1* has high level expression flanking the boundaries and inhibition at the segment centre. This AP patterning fails in *fgf20a*^−*/*−^ embryos (white arrowheads)*. n*=12 per stage and genotype. (B) *Neurod4* expression in WT and *fgf20a*^−*/*−^ embryos at 20 hpf and 30 hpf. Dorsal view. *Neurod4* expression is patterned in a similar manner as *neurog1*, except at the most dorsal extent of the expression. This patterning fails in *fgf20a*^−*/*−^ (red arrowheads). *n*=12 per stage and genotype. (C) HCR RNA-FISH for *neurog1* (magenta) and *neurod4* (cyan) in WT and *fgf20a*^−*/*−^. DAPI (blue). Transverse view at r5. In WT, *neurog1* expression is lower in the segment centre than in the neurogenic zone, while in *fgf20a*^−*/*−^, the expression remains at a high level in the segment centre. For *neurod4*, the expression difference between WT and *fgf20a*^−*/*−^ only become more apparent at later stages. *n*≥8 per stage per genotype. (D) Expression of *neurog1*, *ascl1a*, *ascl1b* and late proneural gene *neurod4* in WT embryo at 42 hpf. DAPI (blue). Transverse sections at the neurogenic region of r5, representative of other hindbrain segments. Early proneural gene expression is patterned along the DV axis. *n*=4. (E) *Ascl1a* (magenta) and *ascl1b* (green) expression in WT and *fgf20a*^−*/*−^ embryos at 42 hpf. DAPI (blue). Dorsal view. AP-patterning of *ascl1a* and *ascl1b* expression failed in *fgf20a*^−*/*−^, resulting in ectopic expression in the segment centre (red arrowheads). Green arrowheads indicate the most dorsal extent of *ascl1b* expression. *n*=12 per genotype. (F) Expression of *ascl1a* in WT and *fgf20a*^−*/*−^ at 42 hpf. DAPI (blue). Dorsal view. The ventral domain of *ascl1a* expression is patterned by Fgf20a signalling. This pattering failed in *fgf20a*^−*/*−^ embryos (red arrowheads). *n*=12 per genotype. (G) Expression of *etv5b* (magenta), *ascl1a* (green) and *ascl1b* (green) in WT and *fgf20a*^−*/*−^ embryos at 36 hpf. DAPI (blue). Dorsal view. *Etv5b* and *ascl1a/b* expression has spatial complementarity in hindbrain segments. Red arrowheads in *fgf20a*^−*/*−^ embryos indicate regions with ectopic upregulation of proneural gene expression. *n*=12 per genotype. (H) Expression of *etv5b* (magenta) and *ascl1a* (green) in WT and *fgf20a*^−*/*−^ embryos at 36 hpf. DAPI (blue). Transverse view at r4. In the segment centre of WT embryos, the ventral domain of *ascl1a* is patterned by Fgf20a signalling, while the dorsal domain, which is beyond the signalling range, remains unpatterned. The patterning failed in *fgf20a*^−*/*−^ embryos with ectopic upregulation of *ascl1a* in the segment centre (white arrowheads)*. n*=6 per genotype. Dashed lines (white) indicate the position of hindbrain boundaries between segments. Scale bars: 30 µm (A-E,G,H); 15 µm (F).

Next, we investigated whether the short-range nature of Fgf20 signalling is linked to the spatially restricted expression of proneural genes. It was shown previously that expression of the early proneural gene *neurog1* and late proneural gene *neurod4* is patterned along the AP axis by Fgf20a signalling (Gonzalez-Quevedo et al., 2010). This patterning emerges from an initially homogeneous expression along the AP axis, resulting in high levels of proneural gene expression in areas flanking the hindbrain boundaries, with inhibition in the segment centre (Fig. 2A,B; Fig. S5). The sensitivity of the HCR technique revealed that a low level of *neurog1* and *neurod4* expression was maintained in the segment centre of wild-type (WT) embryos (Fig. 2C). This pattern failed to emerge in *fgf20a*^−*/*−^ embryos as high levels of *neurog1* and *neurod4* expression persisted throughout the segment (Fig. 2A-C).

In addition to *neurog1*, other early proneural genes, such as *ascl1a* and *ascl1b*, were expressed in the dorsal neural tube of the zebrafish hindbrain (Fig. 2D) (Thisse and Thisse, 2005; Amoyel et al., 2005; Jászai et al., 2003). We observed that the combination of the early proneural genes *ascl1a*, *ascl1b* and *neurog1* spatially overlap with the late proneural gene *neurod4* along both the AP and DV axes (Fig. S5C), consistent with *neurod4* acting downstream of these early proneural genes in this context (Bertrand et al., 2002). The most dorsal domain of *neurod4* expression in the segment centre persisted after downregulation of *neurog1* in the ventral segment centre, suggesting that there is DV patterning of neurogenesis (Fig. S5C) (Gonzalez-Quevedo et al., 2010). We therefore wondered whether early proneural genes are regulated along the DV axis by signalling from the ventrally-located *fgf20*-expressing neurons.

We first examined *ascl1a* and *ascl1b* expression at 42 hpf, when the patterning of proneural gene expression is fully established. *Ascl1b* was expressed broadly along the DV axis of the segment. In WT embryos, it was patterned along the AP axis, except for the most dorsal extent of the expression (Fig. 2E). We found that the AP-patterning of *ascl1b* expression failed to emerge in *fgf20a*^−*/*−^ embryos, with high levels of expression maintained in the segment centre. In contrast, *ascl1a* was expressed in the dorsal hindbrain in two narrow and discrete expression domains (Fig. 2E,F). Importantly, in WT embryos, the AP-patterning of *ascl1a* expression occurred only for the ventral domain, and this patterning was lost in *fgf20a*^−*/*−^ embryos, where ectopic expression was detected in the segment centre (Fig. 2F). Expression analysis of the proneural genes and *etv5b* at 36 hpf revealed high spatial complementarity between Fgf20 signalling and *ascl1a*/*b* (Fig. 2G). Crucially, the extent of Fgf20 signalling in the segment centre, as indicated by *etv5b* expression, did not surpass the ventral edge of the dorsal *ascl1a* domain (Fig. 2H). This suggests that Fgf20 signalling patterns proneural gene expression along both the AP and DV axes and the short-range nature of the signal confers the spatial precision of the patterning.

### Ectopic Fgf20 signalling leads to downregulation of the early proneural gene *neurog1*

**Fig. 3.**
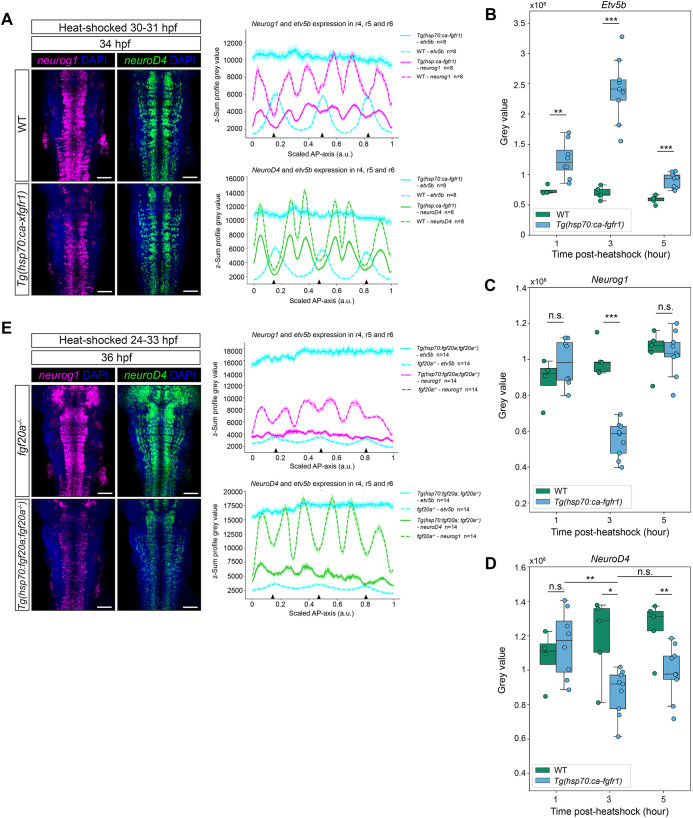
**Fgf20 signalling downregulates proneural gene expression.** (A-E) *Neurog1* (magenta) and *neurod4* (green) are downregulated by overactivation of the Fgfr pathway (A) or overexpression of *fgf20a* (E). Quantifications of HCR RNA-ISH for *etv5b* (B), *neurog1* (C) and *neurod4* (D) are shown as mean grey value measured across r4 to r6 along the AP axis from the confocal micrographs. Each line in A and E represents the mean±s.e.m. of the dataset. Arrowheads indicate the segment centre. (A) WT control and *Tg(hsp70:ca-xfgfr1)* embryos were heat-shocked at 30 hpf for 1 h in WT control and *Tg(hsp70:ca-xfgfr1)* embryos and fixed at 34 hpf. *n*=8 per genotype. (B,C,D) *Neurog1* expression is highly responsive to the level of Fgfr pathway overactivation. WT control and *Tg(hsp70:ca-xfgfr1)* embryos were heat-shocked at 30 hpf for 1 h and fixed at 1 h, 3 h and 5 h post heat-shock. Quantifications of HCR RNA-ISH for *etv5b* (B), *neurog1* (C), and *neurod4* (D) are total grey values measured across r3 to r6 from the confocal micrographs (Fig. S7B). The downregulation of *neurog1* (C) mirrors the overactivation of Fgfr pathway (B), while downregulation of *neurod4* (D) is relatively delayed and damped. Statistical significance was determined using Mann–Whitney *U-*test. n.s., not significant. *P≤0.05, **P≤0.01, ***P≤0.001. (E) *Fgf20a^−/−^ and Tg(hsp70:fgf20a; fgf20a^−/−^)* embryos were heat-shocked from 24 hpf to 33 hpf and fixed at 36 hpf. *n*=14 per genotype. Scale bars: 50 μm.

A prediction of the correlation between spatial expression of Fgf20 and inhibition of proneural gene expression is that ectopic activation of Fgf signalling will lead to a downregulation of *neurog1* expression. To address this, we used a transgenic zebrafish line [*Tg(hsp70:ca-xfgfr1)*] that consists of a heat-shock promotor controlling constitutively active *Xenopus* Fgfr1 (Marques et al., 2008). We performed a time-course analysis of *neurog1* and *neurod4* after *ca-xfgfr1* induction over the course of 32-36 hpf, the period during which hindbrain neurogenesis occurs rapidly (Lyons et al., 2003) (Fig. 3A-D). Heat-shock at 30-31 hpf induced ubiquitous overactivation within 1 h post heat-shock, with the highest level detected at 3 h. This then began to be down-regulated by 5 h (Fig. 3B; Fig. S8)*.* By 3 h post heat-shock, we observed significant downregulation of *neurog1* expression, and thus expression of *neurog1* is sensitive to Fgf signalling levels (Fig. 3B,C; Fig. S8). By 5 h post heat-shock, *neurog1* expression returned to the level seen in WT embryos. In contrast, *neurod4* had a dampened and delayed response to *ca-xfgfr1* induction (Fig. 3D; Fig. S8). These results suggest that Fgf signalling downregulates proneural gene expression in neural progenitors, and that expression of the early proneural gene *neurog1* is more sensitive than the late proneural gene *neurod4*.

**Fig. 4.**
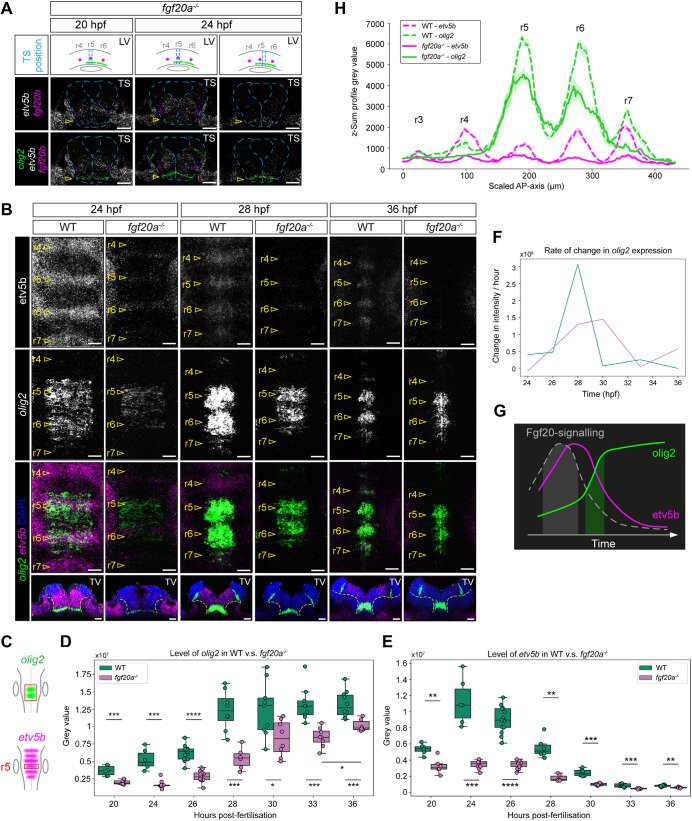
***Olig2* expression is modulated by Fgf20 signalling.** (A) Transverse sections of HCR RNA-FISH for *olig2* (green), *fgf20b* (magenta) and *etv5b* (grey) in *fgf20a*^−*/*−^ embryos at 20 hpf and 24 hpf. Dashed lines outline the neural tube. The positions of the transverse sections along the AP axis are indicated in the schematics. Yellow arrowheads indicate the position where ventral *olig2* expression is initiated. The *olig2* expression colocalised with *etv5b* expression in a ventral region distant from Fgf20b signalling. LV, lateral view; TS, transverse section. *n*=6 per stage. (B-H) Time course analysis of *olig2* expression in the ventral hindbrain of WT and *fgf20a*^−*/*−^ embryo*s* from 20 hpf to 36 hpf. *n*≥6 per stage per genotype. (B) HCR RNA-FISH for *olig2* (green) and *etv5b* (magenta) in WT and *fgf20a*^−*/*−^ embryos. DAPI (blue). Dorsal view across r4 to r7, showing stages representative of the transition in *olig2* expression pattern in the ventral hindbrain. Dashed lines indicate the boundary between the ventricular zone and the mantle zone. (C) Schematics showing the area of quantification of *olig2* (D) and *etv5b* (E) expression. (D) Box plot showing quantification of ventral *olig2* expression across r4 to r7 in A. Individual data points are shown, each representing measurement from an embryo. (E) Box plot showing quantification of *etv5b* expression of r5 in A, as a representative readout of Fgf20 signalling activity. Statistical significance was determined using Mann-Whitney *U*-test. *P≤0.05, **P≤0.01, ***P≤0.001, ****P≤0.0001. (F) Plot showing the temporal relationship between the estimated timing of Fgf20 signalling (grey line), based on the dynamics of *olig2* (D, green line) and *etv5b* (E, magenta line) expression in WT. (G) Plot showing the rate of change in *olig2* expression in D. (H) Expression profile of *olig2* and *etv5b* along the AP axis at 36 hpf in WT and *fgf20a*^−*/*−^ embryos in B*.* Each line represents the mean±s.e.m. of the dataset. *n*=5 for each dataset. Scale bars: 30 µm (A); 20 µm (B).

We assessed the ability of neural progenitors in the hindbrain to respond to Fgf20 signalling by generating a transgenic line [*Tg(hsp70:fgf20a;fgf20a*^−/−^*)*] with heat-shock promotor controlling Fgf20a expression in the *fgf20a*^−*/*−^ background. The ubiquitous overexpression of *fgf20a* at 24-25 hpf resulted in upregulation of *etv5b* and *spry1* in all hindbrain neural progenitors, including hindbrain boundary cells (Fig. S6). This result suggests that all hindbrain neural progenitors are receptive to Fgf20 signalling. Similar to the overactivation of Fgfr in *Tg(hsp70:ca-xfgfr1)*, the ubiquitous expression of *fgf20a* led to significant downregulation of proneural gene expression (Fig. 3E). Together, these results suggest that sustained Fgf20 signalling is required to downregulate proneural gene expression in the segment centre. Neuronal organisation following a loss of proneural gene patterning, either from the overexpression of *fgf20a* or loss-of-function of *fgf20a*, was markedly disorganised compared with WT embryos (Fig. S7).

### *Olig2* expression in the ventral hindbrain is modulated by Fgf20 signalling

The Fgf20-dependent downregulation of proneural gene expression is accompanied by the upregulation of several markers in the segment centre (Gonzalez-Quevedo et al., 2010; Tambalo et al., 2020), including those associated with gliogenesis, such as *sox9b* and *metrnla* (Fig. S15) (Lee et al., 2010; Nishino et al., 2004; Stolt et al., 2003). This points to the possibility that a function of Fgf20 signalling in the hindbrain is to regulate the transition from neurogenesis to gliogenesis. The study of gliogenesis has been challenging, as canonical glial markers are often also expressed in pluripotent neural stem cells (Than-Trong and Bally-Cuif, 2015). For example, *glial fibrillary acidic protein* (*gfap*) labelled a subset of progenitors committed to neuronal differentiation (Fig. S9). We also examined the expression of glutamine synthetase (GS), a marker of mature astroglia, and did not detect a difference between WT and *fgf20a*^−*/*−^ embryos (Fig. S10). However, as previously suggested, oligodendrogenesis could be a target of Fgf20 signalling (Chandran et al., 2003; Esain et al., 2010; Kessaris et al., 2004).

Olig2 is a key transcription factor that regulates the specification of both oligodendrocytes and somatic motor neurons (Li et al., 2011; Park et al., 2002; Rowitch et al., 2002). OPCs can be identified with specific markers such as Sox10, which is regulated downstream of Olig2 and required for the terminal differentiation of OPCs and regulation of myelin genes (Claus Stolt et al., 2002; Küspert et al., 2011; Liu et al., 2007; Pozniak et al., 2010). Previous studies have shown that *sox9*, a key factor in OPC specification, is downstream of Fgf20 signalling (Gonzalez-Quevedo et al., 2010; Stolt et al., 2003). Furthermore, Esain et al. (2010) found that Fgfr activation is required to specify OPCs and Alcama^+^ abducens motor neurons in the zebrafish hindbrain by promoting *olig2* and *sox9* expression (Zannino and Appel, 2009). Crucially, the timing of when the Fgfr pathway is required for this process overlaps with the period of Fgf20 signalling. We therefore hypothesised that Fgf20 is the ligand responsible for regulating *olig2* expression in the segment centre, and thus the production of OPCs and motor neuron subtypes from *olig2*^+^ progenitors.

We found that *olig2* expression is reduced but not abolished in *fgf20a*^−*/*−^ embryos. At 20 hpf, *olig2* was expressed homogeneously in a ventral layer 1-2 cells thick in r5 and r6 (Fig. S11A). This suggests that Fgf20a signalling is not required for the initiation of *olig2* expression in the ventral hindbrain. We investigated whether Fgf20b contributes to the remaining *olig2* expression observed in *fgf20a*^−*/*−^. Multiplexed HCR RNA-FISH in *fgf20a*^−*/*−^ for *olig2*, *fgf20b* and *etv5b* showed that *olig2* expression did not correlate with the region of Fgf20b signalling (Fig. 4A). Instead, *olig2* was initiated within a ventral region of Fgfr pathway activation, indicated by expression of *etv5b*, independent of Fgf20b signalling (Fig. 4A). *olig2* expression in *fgf20a*^−*/*−^ embryos can be further inhibited by treatment with the FGFR tyrosine kinase inhibitor SU5402 (Fig. S14). Together, these results suggest that the initiation of *olig2* expression in the ventral hindbrain is largely independent of Fgf20 signalling. Nonetheless, we found that there are altered levels, pattern and dynamics of *olig2* expression in *fgf20a*^−*/*−^ embryos. In a time course analysis, we found that *olig2* expression was upregulated at 20-36 hpf in both WT and *fgf20a*^−*/*−^ embryos (Fig. 4B-D). Quantification of *olig2* in r5 and r6 shows that the expression levels in *fgf20a*^−*/*−^ embryos were significantly lower than in WT embryos at all time points assessed (Fig. 4B-D; Fig. S11B), suggesting that Fgf20 signalling promotes *olig2* expression. In addition to the ventral expression, a distinct *olig2*^+^ region occurred in the dorsal hindbrain from around 28 hpf onwards. Unlike the ventral expression, this was unaffected in *fgf20a*^−*/*−^ embryos or by *fgf20a* overexpression (Fig. 6D; Fig. S11D), and thus Fgf20 modulated *olig2* expression exclusively in the ventral hindbrain. Crucially, analysis of the expression dynamics in r5 and r6 of WT embryos revealed a rapid, 6.5-fold increase in *olig2* upregulation over the course of 2 h, between 26 and 28 hpf in WT embryos (Fig. 4F). This occurred ∼2 h after the peak of Fgf20 signalling (Fig. 4E; Fig. S11C). In contrast, *olig2* expression in *fgf20a*^−*/*−^ embryos lacked such rapid upregulation, with a maximum of 2.2-fold increase between 26 and 28 hpf. This suggests that Fgf20 signalling upregulates ventral *olig2* expression in r5 and r6 (Fig. 4G).

In addition, *olig2* expression occurred in the segment centre of r4 and r7 by 36 hpf and 26 hpf, respectively (Fig. 4B,H; Fig. S11E), after the initiation of Fgf20 signalling. Furthermore, no *olig2* expression was detected in r4 of *fgf20a*^−*/*−^ embryos, while only low level expression was detected in r7 by 33-36 hpf – a 10 h delay and 3-fold less than the expression level in WT embryos (Fig. 4B,H; Fig. S11E). This suggests that Fgf20 signalling is essential for the timely expression of *olig2* in ventral r4 and r7. Interestingly, we observed an increase in the upregulation of *olig2* expression in r5 and r6 of *fgf20a*^−*/*−^ embryos between 33 and 36 hpf (Fig. 4D,F), whereas in WT embryos *olig2* expression stabilised from 28 hpf onwards. This suggests a compensatory mechanism in *fgf20a*^−*/*−^ embryos to restore *olig2* expression levels. Nonetheless, the later upregulation did not result in a well-defined expression pattern in the segment centre, unlike WT embryos (Fig. 4B). Interestingly, we observed that *olig2* was ectopically upregulated at the r5/6 boundary in *fgf20a*^−*/*−^ embryos by 36 hpf (Fig. S11E), suggesting that boundary cells may have a role in the compensatory mechanism.

Finally, we assessed whether the observed increase in *olig2* expression is an indirect consequence of Fgf20-dependent proliferation (Kosaka et al., 2006; Lahti et al., 2011; Ma et al., 2009; Raballo et al., 2000). We performed EdU-assay (Fig. S12A-C), phospho-histone H3 (Fig. S12D) and *Mki67* staining (Fig. S12E-G) in the hindbrain from 20-42 hpf. We found that proliferation was not patterned within that period, and no difference in proliferation was observed between WT, *fgf20a*^−*/*−^ and embryos with *fgf20a* overexpression.

### Fgf20 signalling dictates the timing of OPC specification and oligodendrocyte number

Given the role of Fgf20 in modulating *olig2* expression, we next asked to what extent it regulates the timing and production of oligodendrocytes and *alcama*^+^ motor neurons in the hindbrain. *Alcama*^+^ motor neurons in the zebrafish hindbrain are generated from *olig2*^+^ precursors at ∼36 hpf (Zannino and Appel, 2009). The timing of Fgf20 signalling is compatible with it being the primary source of Fgf signalling necessary for the production of these motor neurons (Esain et al., 2010). However, quantification of *alcama* in embryos with normal (WT), low (*fgf20a*^−*/*−^) and high [*Tg(hsp70:fgf20a;fgf20a*^−*/*−^*)*] levels of Fgf20 signalling showed that Fgf20 signalling has only a small, though significant, effect on the production of *alcama*^+^ motor neurons (Fig. S13A)*.* In contrast, inhibition of Fgfr activation at 20-30 hpf with SU5402 resulted in a major reduction in the quantity of *alcama*^+^ motor neurons (Fig. S14). Timed pulses of inhibition in *fgf20a*^−*/*−^ embryos from 16 to 24 h shows that the key window of Fgfr pathway activation that is crucial for *alcama*^+^ motor neurons is 20-24 hpf (Fig. S13B). This suggests that although Fgf20 signalling has some influence on the production of *alcama*^+^ motor neurons, Fgfr pathway activation independent of Fgf20 is responsible for their specification and production.

**Fig. 5.**
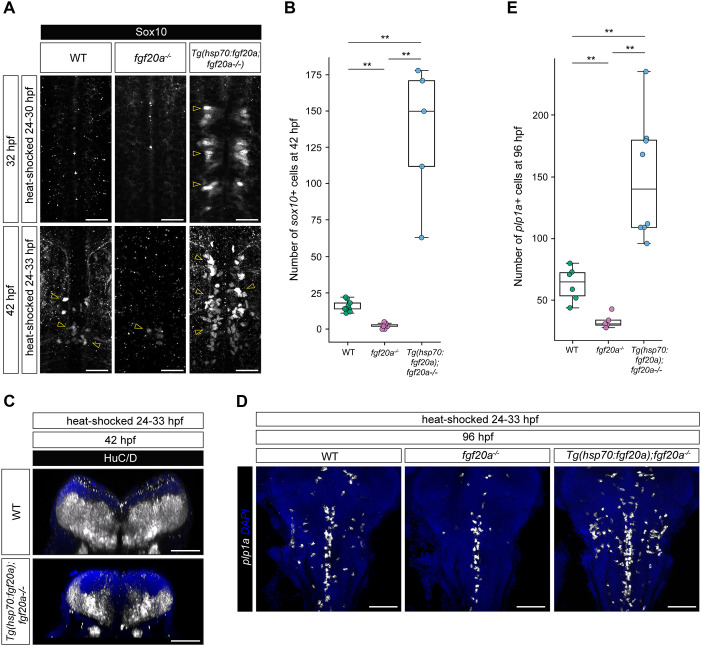
**Fgf20 signalling determines the quantity of oligodendrocytes.** (A) Immunostaining for Sox10 in WT, *fgf20a*^−*/*−^ and *Tg(hsp70:fgf20a; fgf20a*^−*/*−^*)* embryos at 32 hpf and 42 hpf. Embryos at 32 hpf were heat-shocked from 24-30 hpf. Fgf20a overexpression results in precocious specification of *sox10*^+^ OPCs in *Tg(hsp70:fgf20a; fgf20a*^−*/*−^*)* embryos with *fgf20a* overexpression. Embryos at 42 hpf were heat-shocked from 24-33 hpf. The number of *sox10*^+^ OPCs is reduced in *fgf20a*^−*/*−^ compared to WT embryos, whereas *fgf20a* overexpression results in a significant increase in the number of *sox10*^+^ OPCs. *n*=5 (WT 32 hpf); *n*=4 (*fgf20a*^−*/*−^ 32 hpf); *n*=7 [*Tg(hsp70:fgf20a; fgf20a*^−*/*−^*)* 42 hpf]; *n*=9 (WT 42 hpf); *n*=9 (*fgf20a*^−*/*−^ 42 hpf); *n*=15 [*Tg(hsp70:fgf20a; fgf20a*^−*/*−^*)* 42 hpf]. Yellow arrowheads indicate some Sox10^+^ OPCs. (B) Box plot showing quantification of A. Statistical significance was determined using Mann–Whitney *U-*test. **P≤0.01. (C) Immunostaining for HuC/D in WT and *Tg(hsp70:fgf20a; fgf20a*^−*/*−^*)* embryos at 42 hpf, heat-shocked from 24-33 hpf. Transverse view at r5. *Tg(hsp70:fgf20a; fgf20a*^−*/*−^*)* embryos with *fgf20a* overexpression result in marked reduction in the volume of the mantle zone. *n*=9 (WT 42 hpf); *n*=15 [*Tg(hsp70:fgf20a; fgf20a*^−*/*−^*)* 42 hpf]. (D) HCR RNA-FISH for *plp1a* in WT (*n*=6), *fgf20a*^−*/*−^ (*n*=5) and *Tg(hsp70:fgf20a; fgf20a*^−*/*−^*)* (*n*=8) embryos at 96 hpf, heat-shocked from 24-33 hpf. The number of *plp1a*^+^ oligodendrocytes is reduced in *fgf20a*^−*/*−^ compared to that of WT. *fgf20a* overexpression results in a significant increase in the number of *plp1a*^+^ oligodendrocytes. (E) Box plot showing quantification of D. Statistical significance was determined using Mann–Whitney *U-*test. **P≤0.01. Scale bars: 20 µm (A); 50 µm (C); 70 µm (D).

Next, we examined the effects of the amount of Fgf20 signalling on the production of OPCs. In WT embryos, Sox10^+^ OPCs were detectable in the posterior hindbrain from ∼42 hpf (Fig. 5A). We found that the number of OPCs in *fgf20a*^−*/*−^ embryos was 7-fold less than in WT embryos (Fig. 5B). Remarkably, overexpression of *fgf20a* throughout the hindbrain at 24-30 or 24-33 hpf resulted in a 9-fold increase in the number of OPCs (Fig. 5B). Furthermore, the overexpression of *fgf20a* also resulted in precocious specification of OPCs by 32 hpf (Fig. 5A). These results suggest that the number of Fgf20-expressing cells influences both the quantity and timing of OPC specification. Parallel to the gliogenic effect, in alignment with earlier observations on proneural gene expression, *fgf20a* overexpression resulted in a substantial reduction in neuronal production, as exemplified by the marked decrease in thickness of the mantle zone (Fig. 5C). This points to the overarching pro-gliogenic functions of Fgf20 signalling in the developing hindbrain.

**Fig. 6.**
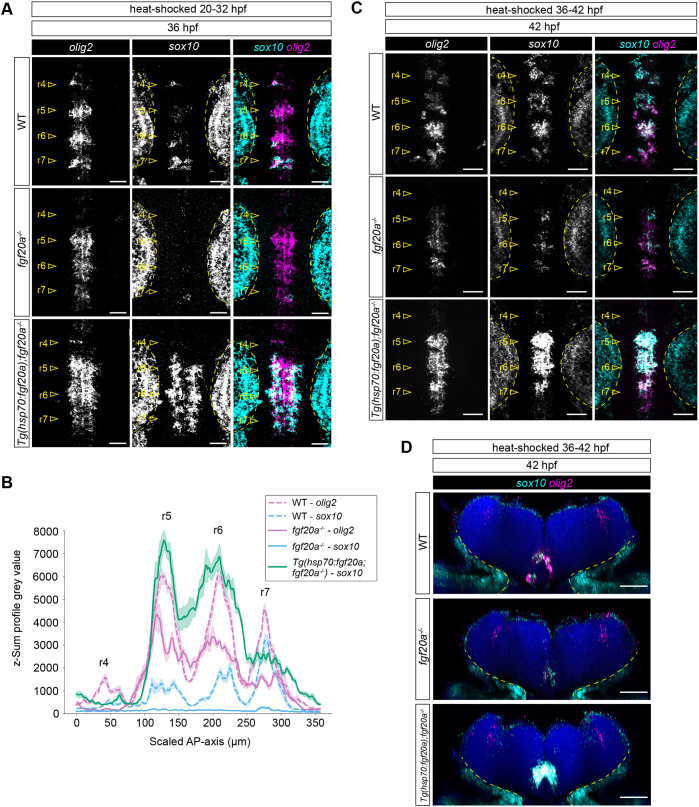
**Fgf20 signalling upregulates *olig2* and *sox10* expression in competent progenitors.** (A) HCR RNA-FISH for *olig2* (magenta) and *sox10* (cyan) in WT (*n*=6), *fgf20a*^−*/*−^ (*n*=6) and *Tg(hsp70:fgf20a; fgf20a*^−*/*−^*)* (*n*=9) embryos at 36 hpf, heat-shocked from 20-32 hpf. (B) Expression profile of *olig2* and *sox10* expression along the AP axis across r4 to r7 from A. Each line represents the mean±s.e.m. of the dataset. (C) HCR RNA-FISH for *olig2* (magenta) and *sox10* (cyan) in WT (*n*=6), *fgf20a*^−*/*−^ (*n*=6) and *Tg(hsp70:fgf20a; fgf20a*^−*/*−^*)* (*n*=6) embryos at 42 hpf, heat-shocked from 36-42 hpf. (D) Transverse view of C. Dashed lines in A, C and D demarcate the gene expression in the otic vesicle and nearby neural crest. Scale bars: 20 µm (A); 30 µm (C,D).

To examine whether the effect of Fgf20 on OPCs had the corresponding effects on oligodendrocyte production, we next quantified the number of oligodendrocytes at 96 hpf using the marker *proteolipid protein 1a* (*plp1a*) (Aggarwal et al., 2011) (Fig. 5D)*.* Indeed, we found that the number of *plp1a*^+^ oligodendrocytes in *fgf20a*^−*/*−^ was half that of WT embryos, while in embryos overexpressing *fgf20a* we observed twice as many (Fig. 5E). This shows that the role of Fgf20 signalling in OPC specification is important for later *plp1a*^+^ oligodendrocyte production. Thus, the extent of Fgf20 signalling determines the number of oligodendrocytes produced in the hindbrain.

We next used HCR RNA-FISH to probe how Fgf20 signalling influences the spatiotemporal dynamics of *sox10* and *olig2*, markers that are essential for OPC specification. We examined their expression in WT, *fgf20a*^−*/*−^ and *Tg(hsp70:fgf20a;fgf20a*^−*/*−^*)* embryos at 36 and 42 hpf, time points when *alcama*^+^ motor neurons are produced and Sox10^+^ OPCs start to be detected, respectively (Fig. 6A,B). In WT embryos, *sox10* expression was detected in the segment centres by 36 hpf, within a subset of *olig2*^+^ progenitors (Fig. 6A). Despite the observation that *olig2* levels were highest in r5 and r6 at this stage, *sox10* was most strongly expressed in r7 and co-expressed with *olig2*. This reflects the segmental differences in the lineage propensity for OPCs and the heterogeneity in cell fates among *olig2*^+^ progenitors in r5 and r6. In contrast, *sox10* was not expressed in r4-r6 of *fgf20a*^−*/*−^ at 36 hpf, while a low level of expression was detected in r7. This is congruent with the observation in WT embryos, where r7 appeared to have a higher propensity for *sox10* expression*.* By 42 hpf, a low level of *sox10* expression was detected in a subset of *olig2*^+^ progenitors in r5-7 of *fgf20a*^−*/*−^, but continued to be absent in r4 (Fig. 6C). This suggests that Fgf20 signalling is necessary for the timely expression of *sox10* in *olig2*^+^ progenitors in the ventral hindbrain.

**Fig. 7.**
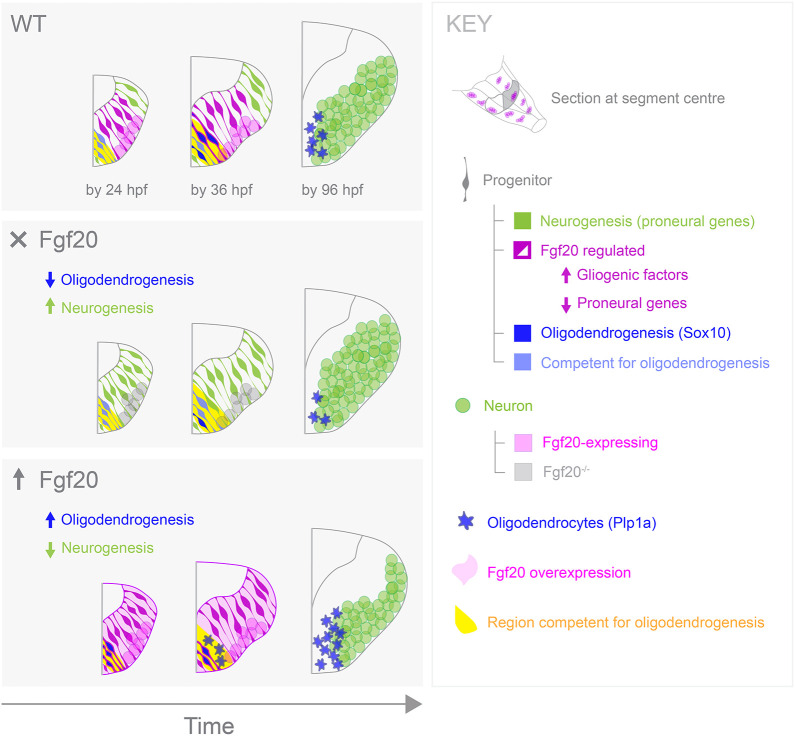
**Fgf20 signalling regulates neurogenesis-to-oligodendrogenesis transition in the developing hindbrain.** Schematics illustrating the role of Fgf20 signalling in promoting oligodendrogenesis while inhibiting neurogenesis in hindbrain progenitors. The wild-type scenario (top) is juxtaposed with conditions either lacking Fgf20 signalling (middle) or with ectopically upregulated Fgf20 signalling (bottom).

Congruent with the earlier observation from immunostaining of Sox10, ubiquitous overexpression of *fgf20a* from 20 to 36 hpf resulted in the precocious specification and differentiation of *sox10*^+^ OPCs among *olig2*^+^ progenitors (Fig. 6A). A shorter, but later, pulse of *fgf20a* overexpression of 6 h from 36-42 hpf resulted in major upregulation of both *olig2* and *sox10* expression among *olig2*^+^ progenitors (Fig. 6C,D), suggesting that *fgf20a* overexpression beyond the normal period of requirement of the signalling is sufficient to induce *olig2* and *sox10* expression in competent cells. Therefore, Fgf20 signalling has an instructive role on the dynamics of a gene regulatory network involving *olig2* and *sox10* (Liu et al., 2007). Together with the inhibition of proneural gene expression, this suggests that Fgf20 signalling induces a switch from neurogenesis to oligodendrogenesis in the developing hindbrain (Fig. 7)*.*

## DISCUSSION

In this study, we provide an example in the vertebrate nervous system in which precise tissue patterning and the induction of late-born cell types are regulated by a short-range signal emanating from earlier-born neurons. Such mechanisms provide temporal checkpoints and are particularly valuable for neural development at later stages where refined spatial and temporal patterning of pre-patterned progenitor pools is needed to further increase cell type diversity. In the zebrafish hindbrain, *fgf20*-expressing neuronal clusters pattern progenitors spatially and temporally to induce the neurogenesis-to-oligodendrogenesis switch in the centre of segments. The signal inhibits neurogenesis in the segment centre through downregulation of proneural genes, while inducing oligodendrogenesis among competent cells in that region through upregulation of OPC specification factors *olig2* and *sox9* (Gonzalez-Quevedo et al., 2010), and differentiation factor *sox10*. We show that Fgf20 is instructive for oligodendrogenesis as the amount of signalling defines the timing of induction and quantity of oligodendrocytes produced.

### Precise tissue patterning by a short-range signal from earlier-born neurons

Whereas other members of the Fgf family act at long-range and form gradients in the nervous system (Yu et al., 2009), Fgf20-signalling does not appear to pattern hindbrain segments as a morphogen. The striking spatial correlation between the position of Fgf20-expressing neurons and progenitors with Fgfr pathway activation suggests that Fgf20 signalling acts at a short-range in the hindbrain. We have shown by ubiquitous overexpression of the ligand that all hindbrain progenitors are able to respond to Fgf20 signalling. The expression pattern of the reporter genes indicate that the signalling is likely to be restricted to the basal region of the progenitors, which are proximal to the signalling source. It is unclear whether Fgf receptors are localised at a specific region along the apical-basal axis of the progenitors. Nonetheless, it has been shown previously that the high tendency of Fgf20 to homodimerise is a dominating factor in determining the signalling activity and diffusion range (Harada et al., 2009; Kalinina et al., 2009; Liu et al., 2017). The homodimerisation acts as an auto-inhibitory mechanism by occluding the receptor binding site and restricting the diffusion range by increasing affinity for heparan sulphate proteoglycans (HSPGs). This is unique to the ligands of the Fgf9/16/20 subfamily, in particular Fgf9 and Fgf20. These properties provide a potential explanation for the highly restricted spatial expression of Fgf20-regulated genes. It also points to the possibility that Fgf20 is a weak signal, as the monomeric form is required for receptor binding.

The production of a short-range signal from a localised source – comprising no more than 8-10 cells arranged in a cluster – is an effective way of achieving precise patterning within the spatial confines of pre-patterned tissues. This is exemplified by the highly restricted expression of proneural genes along both the AP and DV axes of hindbrain segments regulated by the *fgf20*-expressing neuronal clusters. This, in turn, impacts the number and proportion of cell types produced, as well as the structural organisation of the tissue. Intriguingly, it has also been suggested that, in the mouse neocortex, earlier-born neurons secrete Fgf9 to regulate the timing of the switch from neurogenesis to gliogenesis (Seuntjens et al., 2009). This points to the possibility of a common strategy of vertebrate nervous systems in using short-range Fgf signalling to regulate developmental processes that would benefit from high spatial precision. Nonetheless, it is unclear whether the *fgf9*-expressing neurons regulate tissue patterning in the mouse neocortex, as occurs in the hindbrain. The mechanism for the onset of Fgf20 expression in selective nascent neurons in the hindbrain segment centre is not known. Sip1 is a potential candidate, as Seuntjens et al. (2009) have shown that this factor is upstream of *fgf9* expression in the earlier-born neurons of the mouse neocortex.

Another potential significance of short-range localised signals in regulating neuroepithelial development is its ability to influence the timing of cell type formation when factoring in the involvement of interkinetic nuclear migration (INM). Indeed, it has been described in the zebrafish retina (Del Bene et al., 2008) that INM is crucial for modulating the rate of neurogenesis across the progenitor populations by bringing the cell bodies closer to or away from the apically located Notch signalling. An interesting avenue for future investigation is the potential influence of the rate of INM of hindbrain progenitors on the temporal dynamics of Fgf20-mediated gliogenesis.

### Fgf20 signalling induction of oligodendrogenesis

This study has provided evidence that a key function of Fgf20 signalling in the hindbrain is to induce glial cell fate. We found that Fgf20 is an instructive signal for oligodendrogenesis as it controls both the timing and the proportion of oligodendrocytes produced. The commitment of both OPCs and motor neurons from *olig2*^+^ can be attributed to the upregulation of *olig2* (Sagner et al., 2018; Scott et al., 2021; Wegener et al., 2015). It has been suggested, through single cell RNA-sequencing, that the maturation of OPCs is coupled with an increase in the level of *olig2* (Scott et al., 2021). Furthermore, gain-of-function of Olig2 in OPCs can result in precocious differentiation of these cells (Wegener et al., 2015), likely through Olig2 regulation of *sox10* expression (Küspert et al., 2011; Pozniak et al., 2010). In the zebrafish hindbrain, the initiation of *olig2* expression in the ventral hindbrain does not require Fgf20 signalling. However, Fgf20 is necessary for further upregulating *olig2* expression and the timely induction of OPCs. This process appears to be delayed in the loss-of-function of Fgf20, whereas overexpression of Fgf20 leads to precocious specification of OPCs. Expression of *olig2* in Fgf20 loss-of-function mutants has dynamics that suggests a compensatory mechanism is in place to restore the expression level. Nonetheless, the number of oligodendrocytes is ultimately significantly reduced, potentially due to the constraint of a specification time window (Economou et al., 2022). This highlights the role of Fgf20 signalling in ensuring the induction of sufficient OPCs within a time window to attain the correct proportion of oligodendrocytes.

The ubiquitous overexpression of Fgf20 in the hindbrain only induces ectopic oligodendrogenesis among competent progenitors in a ventral domain. In particular, a short burst of Fgf20 overexpression leads to overexpression of *olig2* and *sox10* exclusively in ventral *olig2*^+^ progenitors. This suggests that Fgf20 may be acting on progenitors pre-patterned by Shh signalling along the DV axis. The interplay between Fgf and Shh signalling pathway in oligodendrogenesis has been previously explored. Kessaris et al. (2004) demonstrated *in vitro* that although Fgf can induce OPCs independent of Shh, ligand-independent Fgfr activation is required for Shh-mediated OPC induction. It has been shown that in the chick spinal cord Fgfr activity is required cell-autonomously in Shh-induced *olig2*^+^ progenitors to maintain high level Olig2 expression for the commitment of OPCs (Farreny et al., 2017). In amniotes, Fgf can promote *olig2* expression and OPC induction independent of Shh in the dorsal neural tube (Chandran et al., 2003; Fogarty et al., 2005; Naruse et al., 2006; Vallstedt et al., 2005). However, the *olig2-*expressing domains in the dorsal hindbrain are not affected by Fgf20 overexpression.

We observed that the level of Fgf20 signalling affects *alcama*^+^ motor neuron production with the same directionality as OPC induction, but with a much lesser magnitude of change. Although in this study we have not investigated the role of Fgf20 specifically on bipotential *olig2*^+^ progenitors (Zannino and Appel, 2009), one potential explanation for the mild effects of Fgf20 levels on the production of *alcama*^+^ motor neurons is the opposing effect of the signal in promoting *olig2* expression while downregulating proneural gene expression. An increase in *olig2* expression could lead to an increase in the number of *alcama*^+^ motor neurons, as olig2 promotes proneural gene expression (Mizuguchi et al., 2001; Novitch et al., 2001). However, this effect could be cancelled out by Fgf20-mediated downregulation of proneural genes. Nonetheless, this highlights the gliogenic nature of the signal. Indeed, as shown in previous studies, Fgf20 upregulates the gliogenic factor *sox9* in the segment centre (Gonzalez-Quevedo et al., 2010). *Sox9* has key roles in the specification of astroglia and oligodendrocytes (Poché et al., 2008; Stolt et al., 2003) and has been implicated in the neurogenesis-to-gliogenesis transition (Vong et al., 2015). Crucially, in mouse, *sox9* overexpression can invert the usual sequence of *olig2* upregulation followed by sox10 (Vogel et al., 2020). The present study suggests that Fgf20 acts as a key regulator of the GRN for oligodendrogenesis in the ventral hindbrain to promote the production of oligodendrocytes. Future studies that explore further glial markers will help further understanding of the role of Fgf20 in regulating the neurogenesis-to-gliogenesis transition. As *etv5b* expression is upregulated by ERK1/2 downstream of Fgfr activation, it will also be interesting to determine whether ERK1/2 and/or other targets of Fgf signalling are involved in the switch to gliogenesis.

## MATERIALS AND METHODS

### Zebrafish lines and sample collection

Wild-type *Danio rerio*, *fgf20a*^−*/*−^ (Whitehead et al., 2005), *efnb3b*^−*/*−^ (Cayuso et al., 2019), *Tg(hsp70:ca-xfgfr1)* (Marques et al., 2008) and *Tg(hsp70:fgf20a;fgf20a*^−*/*−^*)* were used in this study. Fgf20a^−*/*−^ line (*fgf20a^zp3^*) contains a point mutation in *fgf20a* resulting in a Y148S amino acid substitution at the β-trefoil region which renders the protein null in function (Whitehead et al., 2005). All mutant embryos used in this study were obtained from homozygous in-crosses. Genotyping of the parents of *fgf20a*^−*/*−^ was performed using a Derived Cleaved Amplified Polymorphic Sequences (dCAPS) assay (https://zebrafish.org/fish/pdf/pcr/zp3.pdf). Genotyping of the parents of *Tg(hsp70:fgf20a;fgf20a*^−*/*−^*)* embryos was performed by fluorescent microscope screening for *cmlc2:EGFP* expression in progeny embryos. Genotyping of the parents of *Tg(hsp70:ca-xfgfr1)* was performed by fluorescent microscope screening for *acr:RFP* expression in progeny embryos. The homozygosity and efficacy of the transgenic lines were further verified by NBT/BCIP *in situ* hybridisation for *etv5b* expression in heat-shocked progeny embryos. Embryos were incubated at 31°C, 28.5°C, 25°C or 22.5°C to regulate the rate of development. They were staged according to the Zebrafish Developmental Staging Series on ZFIN. Embryos at 16-22 hpf were staged according to somite numbers; embryos at 24-42 hpf were staged according to morphological and physiological features. Pigmentation was inhibited by incubation in embryo medium containing 0.003% PTU (1-phenyl 2-thiourea) from 24 hpf.

### Generation of *Tg(hsp70:fgf20a;fgf20a^−/−^)* line

pDestTol2-hsp70:Fgf20a-P2A-H2B-Citrine;cmlc2:EGFP was created by replacing ca-Bmpr1 coding sequence from pDestTol2-hsp70:ca-Bmpr1a;cmlc2:EGFP using the restriction enzyme KpnI (Bielen and Houart, 2012) by a 2010 bp sequence consisting of Fgf20a(stop codon removed)-P2A-H2BCitrine, assembled synthetically using Invitrogen GeneArt. The *Tg(hsp70:fgf20a;fgf20a*^−*/*−^*)* line was generated by injecting the DNA construct pDestTol2-hsp70:Fgf20a-P2A-H2B-Citrine;cmlc2:EGFP (10 ng/μl working concentration) with Tol2 transposase RNA (30 ng/μl working concentration) into one-cell stage embryos from the *fgf20a*^−*/*−^ line. The injection volume was 1.5-2 nl. H2B-Citrine is not expressed in the resulting transgenic line.

### Transient labelling of cell membrane with EGFP-CAAX

mRNA of EGFP-CAAX was synthesized from pCS2-EGFP-CAAX plasmid, linearised with XmnI and transcribed with SP6 polymerase. Then 30 ng/μl of working concentration of mRNA was used for injection into zebrafish embryos at the one- to four-cell stages. Embryos were fixed in 4% paraformaldehyde at the desired stages and stored in 100% methanol at −20°C before proceeding to HCR RNA-FISH.

### Heat-shock protocol

All heat-shocks were performed at 39°C for 1 h in a Petri dish. E3 medium was preheated to 39°C before the heat-shock procedure. PTU was added to the medium for treating embryos beyond 24 hpf. At the end of the heat-shock, the E3 medium in the dish was replaced with E3 medium at room temperature (25°C) and transferred into a 28.5°C incubator. For the protocol that requires prolonged overexpression of the transgenes, repeated heat-shocks were performed. In experiments using *Tg(hsp70:caxfgfr1)*, heat-shock was performed every 3 h. In experiments using *Tg(hsp70:fgf20a;fgf20a*^−*/*−^*)*, heat-shock was performed every 2 h.

### Digoxigenin-based NBT/BCIP *in situ* hybridisation

*In situ* hybridisation and colour development with NBT/BCIP (Roche) were performed as previously described (Xu et al., 1994). The DIG riboprobes for *glula* mRNA were synthesized from PCR products amplified from cDNA of embryos at 30 hpf and 48 hpf, using reverse primers with a T7 promoter site (5′gaaatTAATACGACTCACTATAGg3′). Primers were: forward 5′-GAGATCACTTGTGGGTAGCTC-3′, reverse 5′-GAAATTAATACGACTCACTATAGGGGAGGGAAATTCAGTCCAGTAA-3′.

### HCR RNA-FISH and immunofluorescence staining

Embryos were fixed in 4% paraformaldehyde at room temperature for 3 h and stored in 100% methanol at −20°C for at least 24 h before HCR RNA-FISH or immunostaining. HCR RNA-FISH was performed according to the whole-mount zebrafish embryos and larvae protocol available on the website of Molecular Instruments (molecularinstruments.com). Following rehydration with a graded methanol wash of 75%, 50% and 25% diluted in PBST (PBS with 0.1% Tween 20), proteinease K treatment (10 μg/ml) was performed on embryos at 24 hpf or above. The duration of the treatment ranged from 4 to 30 min, dependent on the stage of the embryos. Post-fixation was performed with 4% paraformaldehyde at room temperature for 20 min.

The following HCR RNA-FISH probe sets, designed and synthesised by Molecular Instruments, were used in the study: *fgf20a*-B1, *fgf20b*-B5, *etv5b*-B2, *spry1*-B5, *neurog1*-B3, *neurod4-*B5, *ascl1a*-B1, *ascl1b*-B4, *olig2*-B3, *sema3gb*-B4, *plp1a*-B4, *sox10*-B5, *alcama*-B1, *mki67*-B1, *metrnla*-B3*.* All probe sets were 20 bases in length and generated based on *Danio rerio* mRNA sequences. HCR amplifiers corresponding to the initiator sequence with Alexa Fluor 488, 514, 546 and 647 were used for detection. Embryos were incubated with the probe for 14 h at the detection stage, then with the fluorescent HCR amplifiers for 12 h at the detection stage.

In experiments with double staining with HCR RNA-FISH and antibodies, immunofluorescence staining was performed after HCR RNA-ISH in 5× SSCT (saline-sodium citrate buffer with 0.1% Tween 20) instead of PBS/PBST. Blocking solution with 5× SSC/0.1% Tween 20 containing 5% goat serum and 1% DMSO were used. Samples were washed and stored in 5× SSC/0.1% Tween 20 before imaging.

The following primary antibodies were used in this study: anti-HuC/D (mouse monoclonal; Invitrogen A-21271; 1:400); anti-Gfap (rabbit polyclonal; Genetex GTX128741; 1:200); anti-EphA4 (rabbit polyclonal; Irving et al., 1996; 1:500); anti-phospho-histone H3 (Ser10) (rabbit polyclonal; Sigma-Aldrich 06-570; 1:200); anti-Sox3 (rabbit polyclonal; Genetex GTX132494; 1:300); anti-Sox10 (rabbit polyclonal; Genetex GTX128374; 1:200). Anti-HuC/D, anti-Gfap and anti-phospho-histone H3 were found to be compatible with HCR RNA-FISH, and anti-EphA4 and anti-Sox3 to be incompatible with HCR RNA-FISH. The following secondary antibodies were used for detection: Alexa Fluor 488 goat anti-rabbit (Invitrogen, A11008; 1:400); Alexa Fluor 488 goat anti-mouse (Invitrogen, A11029; 1:400); Alexa Fluor 546 goat anti-mouse (Invitrogen, A11030; 1:400); Alexa Fluor 546 goat anti-rabbit (Invitrogen, A11071; 1:400); Alexa Fluor 647 goat anti-rabbit (Invitrogen, A31634; 1:400). We used 0.1% Tween 20 in the blocking and washing buffer of all the aforementioned antibody stainings, except anti-Sox10, for which 0.1% Triton X-100 was used. For anti-Sox10 staining, an additional acetone fixation/permeabilisation step was performed after proteinase K treatment. Embryos were placed in 100% acetone at −20°C for 10 mins for anti-Sox10 staining and rinsed in H_2_O before and after the treatment.

### EdU labelling

The Click-iT EdU Cell Proliferation Kit for Imaging with Alexa Fluor 647 dye (Invitrogen, C10340) was used for detection of cells in their S-phase. To label cells with EdU, ∼25 dechorionated embryos were incubated in 1 ml of ice-cold E2 medium with 500 μM of EdU/10% DMSO in a 2 ml microcentrifuge tube on ice for 20 min. The incubation was performed on an orbital shaker at very low rpm. Embryos were transferred to E2 medium at 28.5°C for 15 min for recovery followed by fixation with 4% paraformaldehyde for 3 h at room temperature. Fixed embryos were stored in PBST at 4°C for the later detection of EdU. Permeabilisation was performed before EdU detection. Embryos were placed in 100% acetone at −20°C for 10 min and they were rinsed with H_2_O before and after this treatment. This was followed by 1 h of washing in PBS/1%Triton X-100/1% DMSO. For EdU detection, 500 μl of Click-iT reaction cocktail was added to each tube for 30 min incubation at room temperature, followed by two washes in PBST. Immunofluorescence staining was performed after the EdU detection.

### Pharmacological treatments

Embryos were dechorionated and treated at the specified stages with 6 μM SU5402 (Tocris; 3300). Preliminary data suggested that this concentration is sufficient to inhibit the Fgfr-pathway, indicated by the lack of *etv5b* expression by *in situ* hybridisation. Concentrations above 10 μM are toxic to the embryos. We included 0.003% PTU in treatments of embryos beyond 24 hpf. Embryos were kept in 3 ml E2 embryo medium in low-binding six-well plates during the treatment. For long treatments, the medium was changed every 6-8 h. At least two experiments were performed with 12-15 biological repeats (embryos). All replicates were visually screened on confocal microscopes, with a subset imaged with *z*-stack of the entire volume of the hindbrain*.*

### Image acquisition

Hindbrain samples were flat-mounted on slides in 70% glycerol. Lateral sections (LS) and transverse sections (TS) were generated by imaging manually dissected embryos to expose the desired axis. Unless specified, images were generated from whole-mount samples. Fluorescent-labelled samples were imaged with a 30×/1.05 NA silicone oil immersion objective (Olympus UPLSAPO30XS) on an upright or inverted Olympus FV3000 confocal laser scanning microscope. Images were acquired as 12-bit *z*-stacks of 1024×1024 resolution, *z*-step size of 1.5 μm and three times averaging. As silicon oil (*n*=1.405) has a refractive index closer to 70% glycerol (*n*=1.428) than the typical immersion oil (*n*=1.518), spherical aberration-based axial distortion is minimised (Diel et al., 2020). This enables better resolution and deeper 3D imaging. Chromogenic *in situ* hybridisation samples were imaged with 20×/0.75 NA objective on a Zeiss Axioplan2. All images are representative of imaging, biological and experimental replicates.

### Image processing and quantification

All confocal *z*-stack images shown were visualised and generated using the rendering tools ‘Ortho Slicer’ or ‘Oblique Slicer’ in Imaris software; resulting in maximum intensity projections from a selected subset of the image data. The quantifications of the confocal *z*-stack images were performed using FIJI software with semi-automated macro scripts and then further processed and plotted in Python. The image analysis pipeline is as follows. (1) Background subtraction – background value was measured by manually selecting a region within the tissue on a representative slice, followed by subtraction of this value in all slices of the *z*-stack image. It was verified that this measurement was consistent across slices. (2) Sum projection – generating an image with pixel values that are the sum of all pixels with the same *xy* coordinates in the *z*-stack image. (3) Measure pixel intensity – this is performed either by measuring the average pixel intensity in an area of the image defined by a rectangular area, or as a line profile along the AP axis (*y*-axis) of the hindbrain using the ‘Plot Profile’ function in FIJI. In the former method, total grey values of the measured region are generated by the multiplication of the average pixel intensity and the area. The latter method results in a profile plot of grey values along the *y*-axis that are the average pixel intensity of the *x*-axis of the sum projected *z*-stack image. The line thickness is manually set to include only the regions with expression. The latter method is only suitable for expression patterns without significant variation along the medial-lateral axis, and it emphasizes the difference in expression profile along the AP axis. To generate the mean profile plot with standard error of the mean (s.e.m.) from multiple datasets, the datasets were first aligned by trimming the length and re-scaled to a representative sample using Python. The mean profile plot and s.e.m. is then generated by calculating the moving average in an interval size of 1% of the length of the dataset. The result data is denoted as ‘*z*-sum profile grey value’ in the plots. Statistical significance was determined using Mann–Whitney *U*-test in Python with SciPy.

Box plots (or box-and-whiskers plots) were used to visualise and summarise the distribution of the quantification data from HCR signal. The box ranges from the lower (Q1) to the upper (Q3) quartile. The difference between Q3 and Q1 is the interquartile range (IQR), which describes the spread of the data. A line across the box marks the median. The whiskers are lines extending from Q1 and Q3 to the minimum and maximum data point within Q1−1.5×IQR and Q3+1.5×IQR, respectively. Data points beyond that range are defined as outliers.

## Supplementary Material



10.1242/develop.204256_sup1 Supplementary information

## References

[DEV204256C1] Aggarwal, S., Yurlova, L. and Simons, M. (2011). Central nervous system myelin: Structure, synthesis and assembly. *Trends Cell Biol.* 21, 585-593. 10.1016/j.tcb.2011.06.004 21763137

[DEV204256C2] Amoyel, M., Cheng, Y.-C., Jiang, Y.-J. and Wilkinson, D. G. (2005). Wnt1 regulates neurogenesis and mediates lateral inhibition of boundary cell specification in the zebrafish hindbrain. *Development* 132, 775-785. 10.1242/dev.01616 15659486

[DEV204256C3] Barnabé-Heider, F., Wasylnka, J. A., Fernandes, K. J. L., Porsche, C., Sendtner, M., Kaplan, D. R. and Miller, F. D. (2005). Evidence that embryonic neurons regulate the onset of cortical gliogenesis via cardiotrophin-1. *Neuron* 48, 253-265. 10.1016/j.neuron.2005.08.037 16242406

[DEV204256C4] Bertrand, N., Castro, D. S. and Guillemot, F. (2002). Proneural genes and the specification of neural cell types. *Nat. Rev. Neurosci.* 3, 517-530. 10.1038/nrn874 12094208

[DEV204256C5] Bielen, H. and Houart, C. (2012). BMP signaling protects telencephalic fate by repressing eye identity and its Cxcr4-dependent morphogenesis. *Dev. Cell* 23, 812-822. 10.1016/j.devcel.2012.09.006 23079599 PMC7116079

[DEV204256C6] Cayuso, J., Xu, Q., Addison, M. and Wilkinson, D. G. (2019). Actomyosin regulation by EPH receptor signaling couples boundary cell formation to border sharpness. *eLife* 8, e49696. 10.7554/eLife.49696 31502954 PMC6739871

[DEV204256C7] Cepko, C. (2014). Intrinsically different retinal progenitor cells produce specific types of progeny. *Nat. Rev. Neurosci.* 15, 615-627. 10.1038/nrn3767 25096185

[DEV204256C8] Chandran, S., Kato, H., Gerreli, D., Compston, A., Svendsen, C. N. and Allen, N. D. (2003). FGF-dependent generation of oligodendrocytes by a hedgehog-independent pathway. *Development* 130, 6599-6609. 10.1242/dev.00871 14660548

[DEV204256C9] Cheng, Y.-C., Amoyel, M., Qiu, X., Jiang, Y.-J., Xu, Q. and Wilkinson, D. G. (2004). Notch activation regulates the segregation and differentiation of rhombomere boundary cells in the zebrafish hindbrain. *Dev. Cell* 6, 539-550. 10.1016/S1534-5807(04)00097-8 15068793

[DEV204256C10] Choi, H. M. T., Chang, J. Y., Trinh, L. A., Padilla, J. E., Fraser, S. E. and Pierce, N. A. (2010). Programmable in situ amplification for multiplexed imaging of mRNA expression. *Nat. Biotechnol.* 28, 1208-1212. 10.1038/nbt.1692 21037591 PMC3058322

[DEV204256C11] Claus Stolt, C., Rehberg, S., Ader, M., Lommes, P., Riethmacher, D., Schachner, M., Bartsch, U. and Wegner, M. (2002). Terminal differentiation of myelin-forming oligodendrocytes depends on the transcription factor Sox10. *Genes Dev.* 16, 165-170. 10.1101/gad.215802 11799060 PMC155320

[DEV204256C93] Del Bene, F., Wehman, A. M., Link, B. A. and Baier, H. (2008). Regulation of neurogenesis by interkinetic nuclear migration through an apical-basal notch gradient. Cell 134, 1055-1065. 10.1016/j.cell.2008.07.017 18805097 PMC2628487

[DEV204256C12] Deneen, B., Ho, R., Lukaszewicz, A., Hochstim, C. J., Gronostajski, R. M. and Anderson, D. J. (2006). The transcription factor NFIA controls the onset of gliogenesis in the developing spinal cord. *Neuron* 52, 953-968. 10.1016/j.neuron.2006.11.019 17178400

[DEV204256C13] Dessaud, E., Yang, L. L., Hill, K., Cox, B., Ulloa, F., Ribeiro, A., Mynett, A., Novitch, B. G. and Briscoe, J. (2007). Interpretation of the sonic hedgehog morphogen gradient by a temporal adaptation mechanism. *Nature* 450, 717-720. 10.1038/nature06347 18046410

[DEV204256C14] Dias, J. M., Alekseenko, Z., Applequist, J. M. and Ericson, J. (2014). Tgfβ signaling regulates temporal neurogenesis and potency of neural stem cells in the CNS. *Neuron* 84, 927-939. 10.1016/j.neuron.2014.10.033 25467979

[DEV204256C15] Diel, E. E., Lichtman, J. W. and Richardson, D. S. (2020). Tutorial: avoiding and correcting sample-induced spherical aberration artifacts in 3D fluorescence microscopy. *Nat. Protoc.* 15, 2773-2784. 10.1038/s41596-020-0360-2 32737465

[DEV204256C16] Economou, A. D., Guglielmi, L., East, P. and Hill, C. S. (2022). Nodal signaling establishes a competency window for stochastic cell fate switching. *Dev. Cell* 57, 2604-2622.e5. 10.1016/j.devcel.2022.11.008 36473458 PMC7615190

[DEV204256C17] Elsen, G. E., Choi, L. Y., Millen, K. J., Grinblat, Y. and Prince, V. E. (2008). Zic1 and Zic4 regulate zebrafish roof plate specification and hindbrain ventricle morphogenesis. *Dev. Biol.* 314, 376-392. 10.1016/j.ydbio.2007.12.006 18191121 PMC2268115

[DEV204256C18] Esain, V., Postlethwait, J. H., Charnay, P. and Ghislain, J. (2010). FGF-receptor signalling controls neural cell diversity in the zebrafish hindbrain by regulating olig2 and sox9. *Development* 137, 33-42. 10.1242/dev.038026 20023158 PMC2796930

[DEV204256C19] Exelby, K., Herrera-Delgado, E., Garcia Perez, L., Perez-Carrasco, R., Sagner, A., Metzis, V., Sollich, P. and Briscoe, J. (2021). Precision of tissue patterning is controlled by dynamical properties of gene regulatory networks. *Development* 148, dev.197566. 10.1242/dev.197566 PMC7929933 33547135

[DEV204256C20] Farreny, M. A., Agius, E., Bel-Vialar, S., Escalas, N., Khouri-Farah, N., Soukkarieh, C., Pituello, F., Cochard, P. and Soula, C. (2017). FGFs are orchestra conductors of Shh-dependent oligodendroglial fate specification in the ventral spinal cord. *Neural Dev.* 13, 3. 10.1101/233775 PMC5842613 29519242

[DEV204256C21] Fogarty, M., Richardson, W. D. and Kessaris, N. (2005). A subset of oligodendrocytes generated from radial glia in the dorsal spinal cord. *Development* 132, 1951-1959. 10.1242/dev.01777 15790969

[DEV204256C23] Gonzalez-Quevedo, R., Lee, Y., Poss, K. D. and Wilkinson, D. G. (2010). Neuronal regulation of the spatial patterning of neurogenesis. *Dev. Cell* 18, 136-147. 10.1016/j.devcel.2009.11.010 20152184 PMC2822724

[DEV204256C24] Greenfeld, H., Lin, J. and Mullins, M. C. (2021). The BMP signaling gradient is interpreted through concentration thresholds in dorsal-ventral axial patterning. *PLoS Biol.* 19, e3001059. 10.1371/JOURNAL.PBIO.3001059 33481775 PMC7857602

[DEV204256C25] Guillemot, F. (2007). Cell fate specification in the mammalian telencephalon. *Prog. Neurobiol.* 83, 37-52. 10.1016/J.PNEUROBIO.2007.02.009 17517461

[DEV204256C26] Guillemot, F. and Hassan, B. A. (2017). Beyond proneural: emerging functions and regulations of proneural proteins. *Curr. Opin. Neurobiol.* 42, 93-101. 10.1016/j.conb.2016.11.011 28025176

[DEV204256C27] Harada, M., Murakami, H., Okawa, A., Okimoto, N., Hiraoka, S., Nakahara, T., Akasaka, R., Shiraishi, Y. I., Futatsugi, N., Mizutani-Koseki, Y. et al. (2009). FGF9 monomer-dimer equilibrium regulates extracellular matrix affinity and tissue diffusion. *Nat. Genet.* 41, 289-298. 10.1038/ng.316 19219044 PMC2676118

[DEV204256C92] Irving, C., Nieto, M. A., DasGupta, R., Charnay, P. and Wilkinson, D. G. (1996). Progressive spatial restriction of Sek-1 and Krox-20 gene expression during hindbrain segmentation. *Dev. Biol.* 173, 26-38. 10.1006/dbio.1996.0004 8575627

[DEV204256C28] Itoh, M., Kim, C.-H., Palardy, G., Oda, T., Jiang, Y.-J., Maust, D., Yeo, S.-Y., Lorick, K., Wright, G. J., Ariza-McNaughton, L. et al. (2003). Mind bomb is a ubiquitin ligase that is essential for efficient activation of notch signaling by delta. *Dev. Cell* 4, 67-82. 10.1016/S1534-5807(02)00409-4 12530964

[DEV204256C29] Jászai, J., Reifers, F., Picker, A., Langenberg, T. and Brand, M. (2003). Isthmus-to-midbrain transformation in the absence of midbrain-hindbrain organizer activity. *Development* 130, 6611-6623. 10.1242/dev.00899 14660549

[DEV204256C30] Jessell, T. M. (2000). Neuronal specification in the spinal cord:inductive signals and transcriptional codes. *Nat. Rev. Genet.* 1, 20-29. 10.1038/35049541 11262869

[DEV204256C31] Kageyama, R. and Ohtsuka, T. (1999). The Notch-Hes pathway in mammalian neural development. *Cell Res.* 9, 179-188. 10.1038/sj.cr.7290016 10520600

[DEV204256C32] Kalinina, J., Byron, S. A., Makarenkova, H. P., Olsen, S. K., Eliseenkova, A. V., Larochelle, W. J., Dhanabal, M., Blais, S., Ornitz, D. M., Day, L. A. et al. (2009). Homodimerization controls the fibroblast growth factor 9 subfamily's receptor binding and heparan sulfate-dependent diffusion in the extracellular matrix. *Mol. Cell. Biol.* 29, 4663-4678. 10.1128/mcb.01780-08 19564416 PMC2725704

[DEV204256C33] Kessaris, N., Jamen, F., Rubin, L. L. and Richardson, W. D. (2004). Cooperation between sonic hedgehog and fibroblast growth factor/MAPK signalling pathways in neocortical precursors. *Development*. 131, 1289-1298. 10.1242/dev.01027 14960493

[DEV204256C34] Kosaka, N., Kodama, M., Sasaki, H., Yamamoto, Y., Takeshita, F., Takahama, Y., Sakamoto, H., Kato, T., Terada, M., Ochiya, T. et al. (2006). FGF-4 regulates neural progenitor cell proliferation and neuronal differentiation. *FASEB J.* 20, 1484-1485. 10.1096/fj.05-5293fje 16723380

[DEV204256C35] Krumlauf, R. and Wilkinson, D. G. (2021). Segmentation and patterning of the vertebrate hindbrain. *Development* 148, dev186460. 10.1242/dev.186460 34323269 PMC7611710

[DEV204256C36] Küspert, M., Hammer, A., Bösl, M. R. and Wegner, M. (2011). Olig2 regulates Sox10 expression in oligodendrocyte precursors through an evolutionary conserved distal enhancer. *Nucleic Acids Res.* 39, 1280-1293. 10.1093/nar/gkq951 20959288 PMC3045606

[DEV204256C37] Lahti, L., Saarimäki-Vire, J., Rita, H. and Partanen, J. (2011). FGF signaling gradient maintains symmetrical proliferative divisions of midbrain neuronal progenitors. *Dev. Biol.* 349, 270-282. 10.1016/j.ydbio.2010.11.008 21074523

[DEV204256C38] Lee, S. K., Lee, B., Ruiz, E. C. and Pfaff, S. L. (2005). Olig2 and Ngn2 function in opposition to modulate gene expression in motor neuron progenitor cells. *Genes Dev.* 19, 282-294. 10.1101/gad.1257105 15655114 PMC545894

[DEV204256C39] Lee, H. S., Han, J., Lee, S. H., Park, J. A. and Kim, K. W. (2010). Meteorin promotes the formation of GFAP-positive glia via activation of the Jak-STAT3 pathway. *J. Cell Sci.* 123, 1959-1968. 10.1242/jcs.063784 20460434

[DEV204256C40] Leino, S. A., Constable, S. C. J., Streit, A. and Wilkinson, D. G. (2023). Zbtb16 mediates a switch between Fgf signalling regimes in the developing hindbrain. *Development* 150, dev201319. 10.1242/dev.201319 37642135 PMC10508701

[DEV204256C41] Li, H., Paes De Faria, J., Andrew, P., Nitarska, J. and Richardson, W. D. (2011). Phosphorylation regulates OLIG2 cofactor choice and the motor neuron-oligodendrocyte fate switch. *Neuron* 69, 918-929. 10.1016/j.neuron.2011.01.030 21382552 PMC3093612

[DEV204256C42] Liu, Z., Hu, X., Cai, J., Liu, B., Peng, X., Wegner, M. and Qiu, M. (2007). Induction of oligodendrocyte differentiation by Olig2 and Sox10: Evidence for reciprocal interactions and dosage-dependent mechanisms. *Dev. Biol.* 302, 683-693. 10.1016/j.ydbio.2006.10.007 17098222

[DEV204256C43] Liu, Y., Ma, J., Beenken, A., Srinivasan, L., Eliseenkova, A. V. and Mohammadi, M. (2017). Regulation of Receptor Binding Specificity of FGF9 by an Autoinhibitory Homodimerization. *Structure* 25, 1325-1336.e3. 10.1016/j.str.2017.06.016 28757146 PMC5587394

[DEV204256C44] Lowery, L. A. and Sive, H. (2005). Initial formation of zebrafish brain ventricles occurs independently of circulation and requires the nagie oko and snakehead/atp1a1a.1 gene products. *Development* 132, 2057-2067. 10.1242/dev.01791 15788456

[DEV204256C45] Lyons, D. A., Guy, A. T. and Clarke, J. D. W. (2003). Monitoring neural progenitor fate through multiple rounds of division in an intact vertebrate brain. *Development* 130, 3427-3436. 10.1242/dev.00569 12810590

[DEV204256C46] Ma, D. K., Ponnusamy, K., Song, M. R., Ming, G. L. and Song, H. (2009). Molecular genetic analysis of FGFR1 signalling reveals distinct roles of MAPK and PLC1 activation for self-renewal of adult neural stem cells. *Mol. Brain* 2, 1-14. 10.1186/1756-6606-2-16 19505325 PMC2700800

[DEV204256C47] Maier, E. C. and Whitfield, T. T. (2014). RA and FGF signalling are required in the Zebrafish Otic vesicle to pattern and maintain ventral otic identities. *PLoS Genet.* 10, e1004858. 10.1371/journal.pgen.1004858 25473832 PMC4256275

[DEV204256C48] Marques, S. R., Lee, Y., Poss, K. D. and Yelon, D. (2008). Reiterative roles for FGF signaling in the establishment of size and proportion of the zebrafish heart. *Dev. Biol.* 321, 397-406. 10.1016/j.ydbio.2008.06.033 18639539 PMC2752040

[DEV204256C49] Maves, L., Jackman, W. and Kimmel, C. B. (2002). FGF3 and FGF8 mediate a rhombomere 4 signaling activity in the zebrafish hindbrain. *Development* 129, 3825-3837. 10.1242/dev.129.16.3825 12135921

[DEV204256C50] Miller, F. D. and Gauthier, A. S. (2007). Timing is everything: making neurons versus glia in the developing cortex. *Neuron* 54, 357-369. 10.1016/j.neuron.2007.04.019 17481390

[DEV204256C51] Mizuguchi, R., Sugimori, M., Takebayashi, H., Kosako, H., Nagao, M., Yoshida, S., Nabeshima, Y., Shimamura, K. and Nakafuku, M. (2001). Combinatorial roles of Olig2 and Neurogenin2 in the coordinated induction of pan-neuronal and subtype-specific properties of motoneurons. *Neuron* 31, 757-771. 10.1016/s0896-6273(01)00413-5 11567615

[DEV204256C52] Naruse, M., Nakahira, E., Miyata, T., Hitoshi, S., Ikenaka, K. and Bansal, R. (2006). Induction of oligodendrocyte progenitors in dorsal forebrain by intraventricular microinjection of FGF-2. *Dev. Biol.* 297, 262-273. 10.1016/j.ydbio.2006.05.017 16782086

[DEV204256C53] Nishino, J., Yamashita, K., Hashiguchi, H., Fujii, H., Shimazaki, T. and Hamada, H. (2004). Meteorin: A secreted protein that regulates glial cell differentiation and promotes axonal extension. *EMBO J.* 23, 1998-2008. 10.1038/sj.emboj.7600202 15085178 PMC404322

[DEV204256C54] Novitch, B. G., Chen, A. I. and Jessell, T. M. (2001). Coordinate regulation of motor neuron subtype identity and pan-neuronal properties by the bHLH repressor Olig2. *Neuron* 31, 773-789. 10.1016/S0896-6273(01)00407-X 11567616

[DEV204256C55] Papalopulu, N. and Kintner, C. (1996). A posteriorising factor, retinoic acid, reveals that anteroposterior patterning controls the timing of neuronal differentiation in Xenopus neuroectoderm. *Development* 122, 3409-3418. 10.1242/dev.122.11.3409 8951057

[DEV204256C56] Park, H. C. and Appel, B. (2003). Delta-Notch signaling regulates oligodendrocyte specification. *Development* 130, 3747-3755. 10.1242/dev.00576 12835391

[DEV204256C57] Park, H. C., Mehta, A., Richardson, J. S. and Appel, B. (2002). Olig2 is required for zebrafish primary motor neuron and oligodendrocyte development. *Dev. Biol.* 248, 356-368. 10.1006/dbio.2002.0738 12167410

[DEV204256C58] Poché, R. A., Furuta, Y., Chaboissier, M. C., Schedl, A. and Behringer, R. R. (2008). Sox9 is expressed in mouse multipotent retinal progenitor cells and functions in Müller Glial cell development. *J. Comp. Neurol.* 510, 237-250. 10.1002/cne.21746 18626943 PMC4412477

[DEV204256C59] Pozniak, C. D., Langseth, A. J., Dijkgraaf, G. J. P., Choe, Y., Werb, Z. and Pleasure, S. J. (2010). Sox10 directs neural stem cells toward the oligodendrocyte lineage by decreasing Suppressor of Fused expression. *Proc. Natl. Acad. Sci. USA* 107, 21795-21800. 10.1073/pnas.1016485107 21098272 PMC3003047

[DEV204256C60] Raballo, R., Rhee, J., Lyn-Cook, R., Leckman, J. F., Schwartz, M. L. and Vaccarino, F. M. (2000). Basic fibroblast growth factor (Fgf2) is necessary for cell proliferation and neurogenesis in the developing cerebral cortex. *J. Neurosci.* 20, 5012-5023. 10.1523/jneurosci.20-13-05012.2000 10864959 PMC6772267

[DEV204256C61] Raible, F. and Brand, M. (2001). Tight transcriptional control of the ETS domain factors Erm and Pea3 by Fgf signaling during early zebrafish development. *Mech. Dev.* 107, 105-117. 10.1016/S0925-4773(01)00456-7 11520667

[DEV204256C62] Roehl, H. and Nüsslein-Volhard, C. (2001). Zebrafish pea3 and Erm are general targets of FGF8 signaling. *Curr. Biol.* 11, 503-507. 10.1016/S0960-9822(01)00143-9 11413000

[DEV204256C63] Rossi, A. M. and Desplan, C. (2020). Extrinsic activin signaling cooperates with an intrinsic temporal program to increase mushroom body neuronal diversity. *eLife* 9, e58880. 10.7554/eLife.58880 32628110 PMC7365662

[DEV204256C64] Rossi, A. M., Fernandes, V. M. and Desplan, C. (2017). Timing temporal transitions during brain development. *Curr. Opin. Neurobiol.* 42, 84-92. 10.1016/j.conb.2016.11.010 27984764 PMC5316342

[DEV204256C65] Rowitch, D. H. and Kriegstein, A. R. (2010). Developmental genetics of vertebrate glial-cell specification. *Nature*. 468, 214-222. 10.1038/nature09611 21068830

[DEV204256C66] Rowitch, D. H., Lu, Q. R., Kessaris, N. and Richardson, W. D. (2002). An “oligarchy” rules neural development. *Trends Neurosci.* 25, 417-422. 10.1016/s0166-2236(02)02201-4 12127759

[DEV204256C67] Sagner, A. (2024). Temporal patterning of the vertebrate developing neural tube. *Curr. Opin. Genet. Dev.* 86, 102179. 10.1016/j.gde.2024.102179 38490162

[DEV204256C68] Sagner, A., Gaber, Z. B., Delile, J., Kong, J. H., Rousso, D. L., Pearson, C. A., Weicksel, S. E., Melchionda, M., Mousavy Gharavy, S. N., Briscoe, J. et al. (2018). Olig2 and Hes regulatory dynamics during motor neuron differentiation revealed by single cell transcriptomics, *PLoS Biol.* 16, e2003127. 10.1371/journal.pbio.2003127 29389974 PMC5811045

[DEV204256C96] Sagner, A., Zhang, I., Watson, T., Lazaro, J., Melchionda, M. and Briscoe, J. (2021). A shared transcriptional code orchestrates temporal patterning of the central nervous system. *PLoS Biol.* 19, e3001450. 10.1371/journal.pbio.3001450 34767545 PMC8612522

[DEV204256C69] Sasai, N., Kutejova, E. and Briscoe, J. (2014). Integration of signals along orthogonal axes of the vertebrate neural tube controls progenitor competence and increases cell diversity. *PLoS Biol.* 12, e1001907. 10.1371/journal.pbio.1001907 25026549 PMC4098999

[DEV204256C70] Scott, K., O'Rourke, R., Winkler, C. C., Kearns, C. A. and Appel, B. (2021). Temporal single-cell transcriptomes of zebrafish spinal cord pMN progenitors reveal distinct neuronal and glial progenitor populations. *Dev. Biol.* 479, 37-50. 10.1016/j.ydbio.2021.07.010 34303700 PMC8410680

[DEV204256C71] Seuntjens, E., Nityanandam, A., Miquelajauregui, A., Debruyn, J., Stryjewska, A., Goebbels, S., Nave, K. A., Huylebroeck, D. and Tarabykin, V. (2009). Sip1 regulates sequential fate decisions by feedback signaling from postmitotic neurons to progenitors. *Nat. Neurosci.* 12, 1373-1380. 10.1038/nn.2409 19838179

[DEV204256C72] Shi, Y. and Liu, J. P. (2011). Gdf11 facilitates temporal progression of neurogenesis in the developing spinal cord. *J. Neurosci.* 31, 883-893. 10.1523/JNEUROSCI.2394-10.2011 21248112 PMC4112097

[DEV204256C73] Stolt, C. C., Lommes, P., Sock, E., Chaboissier, M. C., Schedl, A. and Wegner, M. (2003). The Sox9 transcription factor determines glial fate choice in the developing spinal cord. *Genes Dev.* 17, 1677-1689. 10.1101/gad.259003 12842915 PMC196138

[DEV204256C74] Sun, Y., Nadal-Vicens, M., Misono, S., Lin, M. Z., Zubiaga, A., Hua, X., Fan, G. and Greenberg, M. E. (2001). Neurogenin promotes neurogenesis and inhibits glial differentiation by independent mechanisms. *Cell* 104, 365-376. 10.1016/S0092-8674(01)00224-0 11239394

[DEV204256C75] Tambalo, M., Mitter, R. and Wilkinson, D. G. (2020). A single cell transcriptome atlas of the developing zebrafish hindbrain. *Development* 147, dev184143. 10.1242/dev.184143 32094115 PMC7097387

[DEV204256C76] Tasic, B., Yao, Z., Graybuck, L. T., Smith, K. A., Nguyen, T. N., Bertagnolli, D., Goldy, J., Garren, E., Economo, M. N., Viswanathan, S. et al. (2018). Shared and distinct transcriptomic cell types across neocortical areas. *Nature* 563, 72-78. 10.1038/s41586-018-0654-5 30382198 PMC6456269

[DEV204256C77] Terriente, J., Gerety, S. S., Watanabe-Asaka, T., Gonzalez-Quevedo, R. and Wilkinson, D. G. (2012). Signalling from hindbrain boundaries regulates neuronal clustering that patterns neurogenesis. *Development* 139, 2978-2979. 10.1242/dev.080135 22764046 PMC3403106

[DEV204256C78] Than-Trong, E. and Bally-Cuif, L. (2015). Radial glia and neural progenitors in the adult zebrafish central nervous system. *Glia* 63, 1406-1428. 10.1002/glia.22856 25976648

[DEV204256C94] Thisse, C. and Thisse, B. (2005). High throughput expression analysis of ZF-models consortium clones. ZFIN Direct Data Submission. (https://zfin.org/ZDB-PUB-051025-1).

[DEV204256C79] Tozer, S., Le Dréau, G., Marti, E. and Briscoe, J. (2013). Temporal control of BMP signalling determines neuronal subtype identity in the dorsal neural tube. *Development* 140, 1467-1474. 10.1242/dev.090118 23462473 PMC3596990

[DEV204256C80] Trivedi, V., Choi, H. M. T., Fraser, S. E. and Pierce, N. A. (2018). Multidimensional quantitative analysis of mRNA expression within intact vertebrate embryos, *Development* 145, dev156869. 10.1242/dev.156869 29311262 PMC5825878

[DEV204256C81] Vallstedt, A., Klos, J. M. and Ericson, J. (2005). Multiple dorsoventral origins of oligodendrocyte generation in the spinal cord and hindbrain. *Neuron* 45, 55-67. 10.1016/j.neuron.2004.12.026 15629702

[DEV204256C82] Vogel, J. K., Weider, M., Engler, L. A., Hillgärtner, S., Schmitt, C., Hermans-Borgmeyer, I. and Wegner, M. (2020). Sox9 overexpression exerts multiple stage-dependent effects on mouse spinal cord development. *Glia* 68, 932-946. 10.1002/glia.23752 31724774

[DEV204256C83] Voltes, A., Hevia, C. F., Engel-Pizcueta, C., Dingare, C., Calzolari, S., Terriente, J., Norden, C., Lecaudey, V. and Pujades, C. (2019). Yap/Taz-TEAD activity links mechanical cues to progenitor cell behavior during zebrafish hindbrain segmentation. *Development* 146, dev176735. 10.1242/dev.176735 31273051

[DEV204256C84] Vong, K. I., Leung, C. K. Y., Behringer, R. R. and Kwan, K. M. (2015). Sox9 is critical for suppression of neurogenesis but not initiation of gliogenesis in the cerebellum. *Mol. Brain* 8, 25. 10.1186/s13041-015-0115-0 25888505 PMC4406026

[DEV204256C85] Walshe, J., Maroon, H., McGonnell, I. M., Dickson, C. and Mason, I. (2002). Establishment of hindbrain segmental identity requires signaling by FGF3 and FGF8. *Curr. Biol.* 12, 1117-1123. 10.1016/s0960-9822(02)00899-0 12121619

[DEV204256C86] Wegener, A., Deboux, C., Bachelin, C., Frah, M., Kerninon, C., Seilhean, D., Weider, M., Wegner, M. and Nait-Oumesmar, B. (2015). Gain of Olig2 function in oligodendrocyte progenitors promotes remyelination. *Brain* 138, 120-135. 10.1093/brain/awu375 25564492 PMC4441088

[DEV204256C87] Whitehead, G. G., Makino, S., Lien, C. L. and Keating, M. T. (2005). Developmental biology: fgf20 is essential for initiating zebrafish fin regeneration. *Science (1979)* 310, 1957-1960. 10.1126/science.1117637 16373575

[DEV204256C88] Wiellette, E. L. and Sive, H. (2004). Early requirement for fgf8 function during hindbrain pattern formation in Zebrafish. *Dev. Dyn.* 229, 393-399. 10.1002/dvdy.10464 14745965

[DEV204256C95] Xu, Q., Holder, N., Patient, R. and Wilson, S. W. (1994). Spatially regulated expression of three receptor tyrosine kinase genes during gastrulation in the zebrafish. *Development* 120, 287-299. 10.1242/dev.120.2.287 8149909

[DEV204256C89] Yu, S. R., Burkhardt, M., Nowak, M., Ries, J., Petráek, Z., Scholpp, S., Schwille, P. and Brand, M. (2009). Fgf8 morphogen gradient forms by a source-sink mechanism with freely diffusing molecules. *Nature* 461, 533-536. 10.1038/nature08391 19741606

[DEV204256C90] Zannino, D. A. and Appel, B. (2009). Olig2+ precursors produce abducens motor neurons and oligodendrocytes in the zebrafish hindbrain. *J. Neurosci.* 29, 2322-2333. 10.1523/JNEUROSCI.3755-08.2009 19244509 PMC2720165

[DEV204256C91] Zhao, J., Lin, Q., Kim, K. J., Dardashti, F. D., Kim, J., He, F. and Sun, Y. (2015). Ngn1 inhibits astrogliogenesis through induction of miR-9 during neuronal fate specification. *eLife* 4, e06885. 10.7554/eLife.06885 26271009 PMC4577824

