# Supplementary material for: Short-range Fgf signalling patterns hindbrain progenitors to induce the neurogenesis-to-oligodendrogenesis switch
Source: Development. 2024 Dec 13;151(24):dev204256. doi: 10.1242/dev.204256 (PMC11664172; doi:10.1242/dev.204256)
Supplement: Supplementary information [file develop-151-204256-s1.pdf]

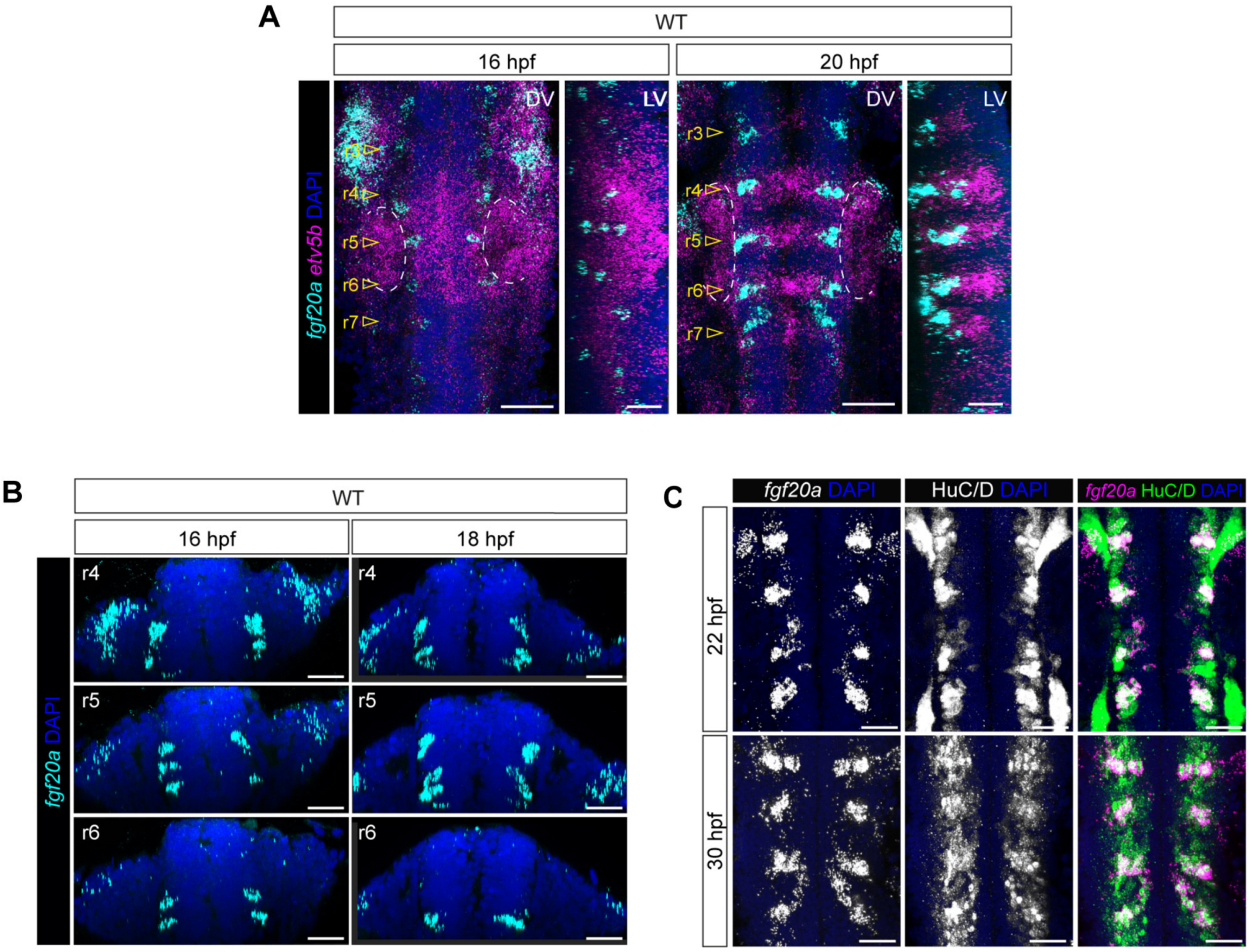

Fig. S1. Fgf20a-expressing neurons in the hindbrain.

(A) HCR RNA-FISH for *fgf20a* (cyan) and *etv5b* (magenta) in WT embryo at 16 hpf (n=12) and 20 hpf (n=12). Three-channel confocal micrograph. DV=dorsal view. LV=lateral view. The broad *etv5b* expression derives from earlier Fgf3/8 signalling in the hindbrain which becomes restricted to the hindbrain segment centre by 20 hpf, regulated by Fgf20a signalling. Dotted lines demarcate the expression in the otic vesicles.

(B) Transverse views of (A), showing HCR RNA-FISH for *fgf20a* (cyan) and DAPI (blue). Three-channel confocal micrograph. Fgf20a-expressing neurons have different DV identities.

(C) Double-staining of HCR RNA-FISH for *fgf20a* (magenta) and immunostaining for the pan-neuronal marker, HuC/D (green), in WT embryos at 22 hpf (n=12) and 30 hpf (n=12). Three-channel confocal micrograph. Fgf20a-expressing neurons are subsets of earlier-born neurons in the segment centre. DAPI (blue). Scale bar: 30 µm

Development • Supplementary information

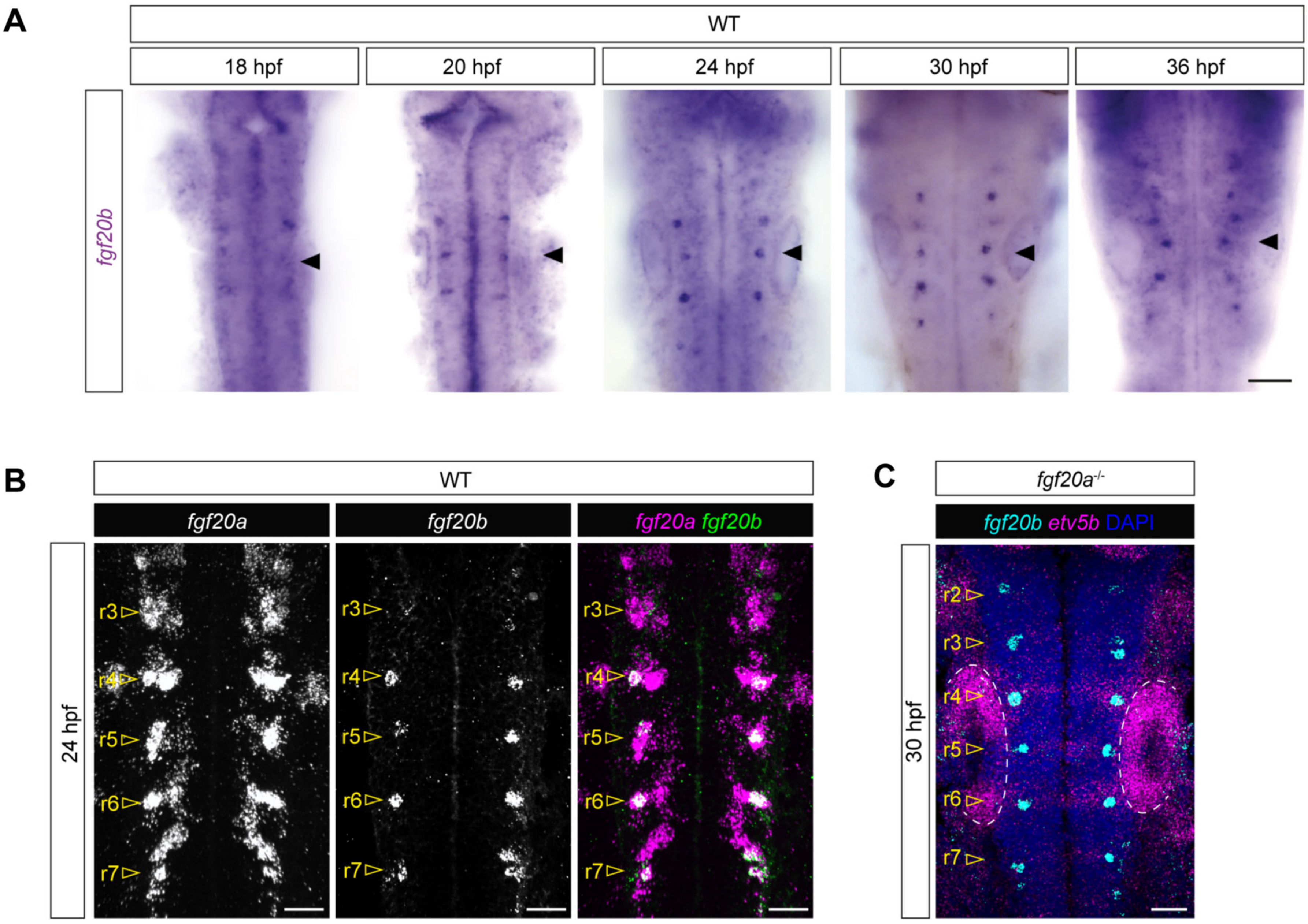

Fig. S2. Fgf20b-expressing neurons in the hindbrain.

(A) BCIP-NBT in-situ hybridisation for *fgf20b* in WT embryos at stages from 18-36 hpf. Arrows indicate r5. Dorsal view. n≥20 per stages. Scale bar: 50 µm

(B) HCR RNA-FISH for *fgf20a* (magenta) and *fgf20b* (green) in WT embryo at 24 hpf (n=12). Three-channel confocal micrograph. Fgf20b is expressed in 1-2 of the Fgf20a-expressing neurons per cluster. Dorsal view. Scale bar: 30 µm

(C) HCR RNA-FISH for *fgf20b* (cyan) and *etv5b* (magenta) in *fgf20a*$^{-/-}$ embryo at 30 hpf (n=12). DAPI (blue). Three-channel confocal micrograph. Fgf20b signalling contributes to a low level of Fgfr pathway activation in the segment centre. Dotted lines demarcate the expression in the otic vesicles. Scale bar: 30 µm

Development • Supplementary information

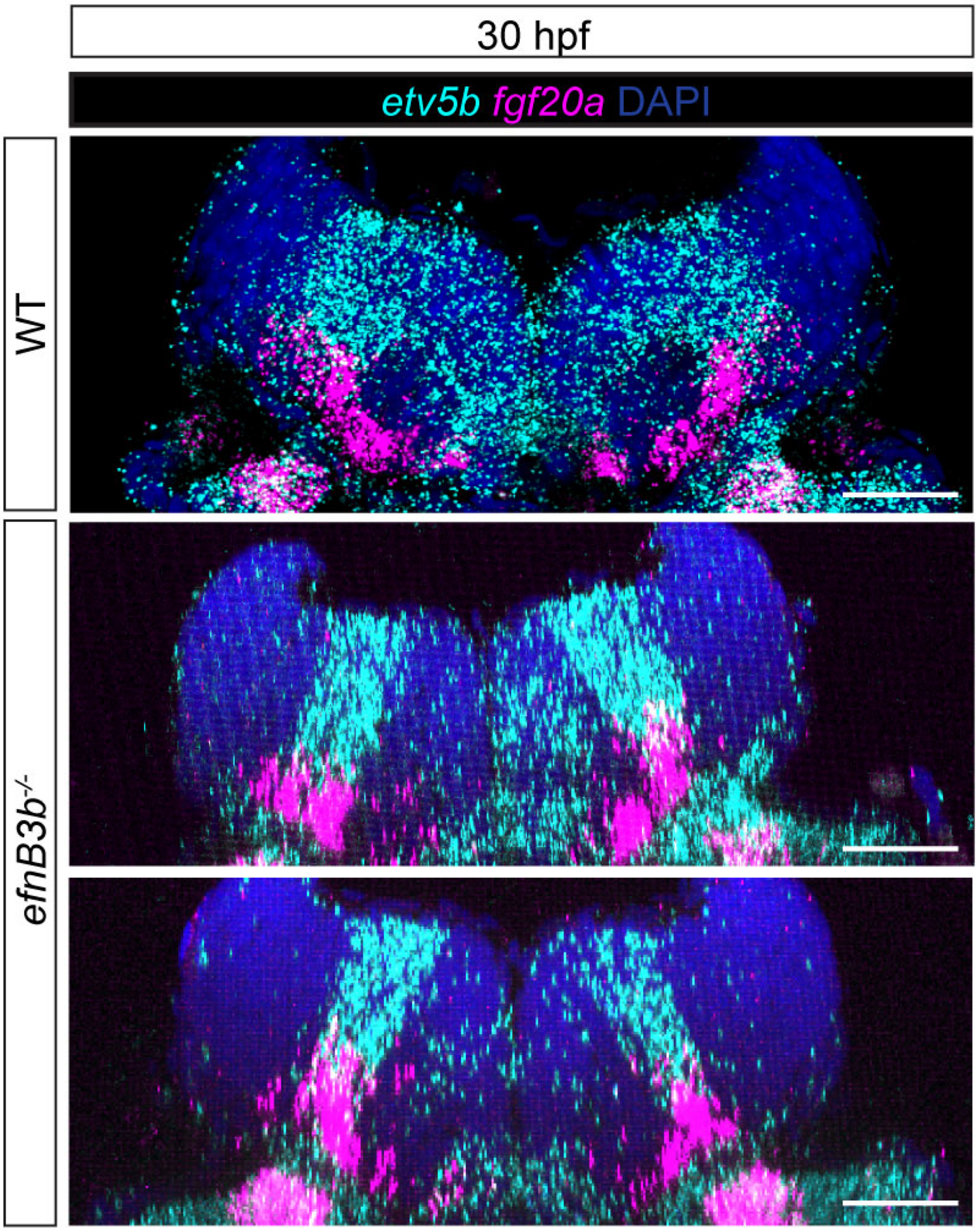

Fig. S3. Altered *etv5b* expression pattern in *efnB3b*$^{-/-}$ mutants correlates with the mispositioned Fgf20-expressing neurons.

HCR RNA-FISH for *etv5b* (cyan) and *fgf20a* (magenta) in WT (n≥12) and *efnB3b*$^{-/-}$ embryos (n≥20) at 30 hpf. DAPI (blue). Three-channel confocal micrographs. Transverse view of r5. Two examples of variations of mispositioned *fgf20*-expressing neurons in *efnB3b*$^{-/-}$ embryos are shown, displaying tight spatial correlation between altered *etv5b* expression in the ventricular zone and positioning of the *fgf20*-expressing neurons, indicative of Fgf20 signalling acting at a short range. Scale bar: 30 µm

Development • Supplementary information

```
FGF20(Human)      MAPLAE-VGGFLGGLEGLGQQVGSHFLLPPAGERPPLLGERRSAAERSARGGPGAAQLAH      59
Fgf20a            MGAVGELVGSFSHGLESFA--HGSHFVLTPPGDSGALMNEHLVSAERLSRS--TSPDLTH      56
Fgf20b            MAAMGE-IGTFLQSLEGFGQ-VGSQFFLPPLGENLGLLNDHLFPAERLSRS--TSADLTH      56
                  *. :.* :* *  .**.:.   **:*.* * *:   *:.::   *** :*.   : :*:*

FGF20(Human)      LHGILRRRQLYCRTGFHLQILPDGSVQGTRQDHSLFGILEFISVAVGLVSIRGVDSGLYL      119
Fgf20a            LKGILRRRQLYCRTGYHLEILPDGSVQGTRKDHSRFGILEFISLAVGMVSIRGVDSGLYL      116
Fgf20b            LKGILRRRQLYCRTGFHLEILPDGSVQGTRKDHSRFGILEFISLAVGLISIRGVDSGLYL      116
                  *:*************:**:***********:*** ********:***::***********

FGF20(Human)      GMNDKGELYGSEKLTSECIFREQFEENWYNTYSSNIYKHGDTGRRYFVALNKDGTPRDGA      179
Fgf20a            GMNSKGELYGSEKLSAECVFREQFEENWYNTYSSNLYRHGERGSHYYVALNKDGTSRDGA      176
Fgf20b            GMNDKGELYGSEKLTAECVFREQFEENWYNTYSSNLYKHGERGARYFVALNKDGTPRDGT      176
                  ***.**********::**:****************:*:**: * :*:******** ***:

FGF20(Human)      RSKRHQKFTHFLPRPVDPERVPELYKDLLMYT    211
Fgf20a            RSRRHQRFTHFLPRPVDPERVPELYKDVLGH-    207
Fgf20b            KSRRHQKFTHFLPRPVDPEKVPELYKEVLGHS    208
                  :*:***:************:******::* :
```

Hydrophobic interactions

Hydrogen bonds

Aromatic and pi-cation interactions

Fig. S4. Conserved residues of Fgf20 that contributes to the inhibitory homodimerisation of the ligands.
Multiple protein sequence alignment between human FGF20 and zebrafish Fgf20a and Fgf20b. * = perfect alignment; : = a site belonging to group exhibiting strong similarities; . = site belonging to a group exhibiting weak similarity. Residues contributing to homodimerisation through hydrophobic interaction (cyan,blue), hydrogen bond (yellow, red) and aromatic/pi-cation interaction (green,black) are conserved.

Development • Supplementary information

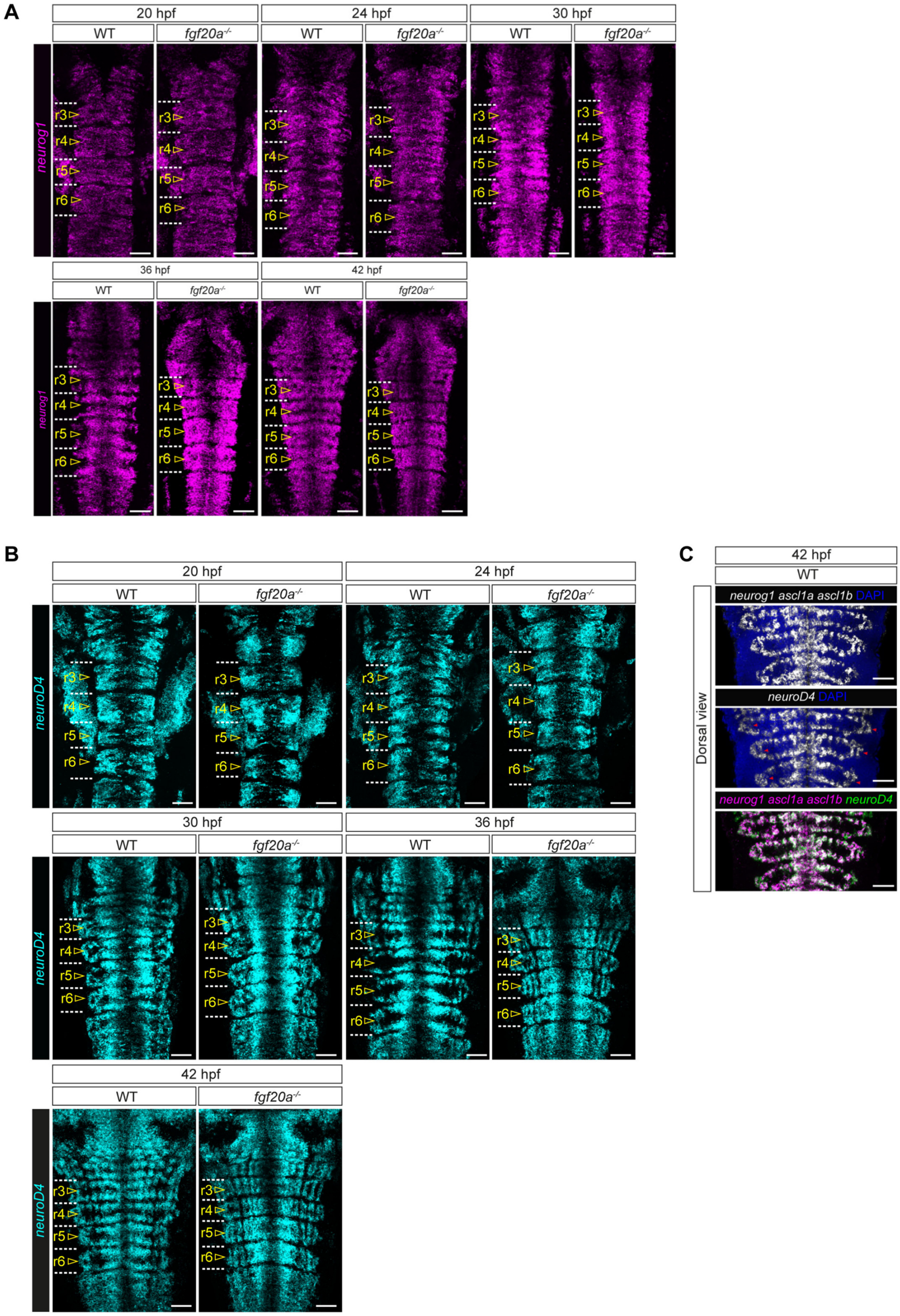
A
20 hpf
24 hpf
30 hpf
WT
fgf20a-/-
neurog1
r3
r4
r5
r6
36 hpf
42 hpf
B
neuroD4
C
42 hpf
Dorsal view
neurog1 ascl1a ascl1b DAPI
neuroD4 DAPI
neurog1 ascl1a ascl1b neuroD4

Fig. S5. *Neurog1* and *neuroD4* expression in WT and *fgf20a*$^{-/-}$ embryos.
(A) HCR RNA-FISH for *neurog1* (magenta) in WT and *fgf20a*$^{-/-}$ at stages from 20-42 hpf.

(B) HCR RNA-FISH for *neuroD4* (cyan) in WT and *fgf20a*$^{-/-}$ at stages from 20-42 hpf. In WT embryos, proneural gene patterning with high level expression flanking the hindbrain boundaries and inhibition in the segment centre emerges over time. This patterning fails to emerge in *fgf20a*$^{-/-}$ embryos. Dotted horizontal lines indicate position of hindbrain boundaries. Dorsal view. Section thickness: 30-40 µm. n=12 per stages. Scale bar: 30 µm (C) *Neurog1*, *ascl1a*, *ascl1b* and late proneural gene *neuroD4* in WT at 42 hpf. DAPI (blue). Five-channel confocal micrograph. The early proneural gene expression is highly correlated to *neuroD4* expression, suggesting that *neuroD4* is downstream of these early proneural genes. Red arrows indicate the most dorsal extent of *neuroD4* expression that is not patterned along the AP-axis. n=8

Development • Supplementary information

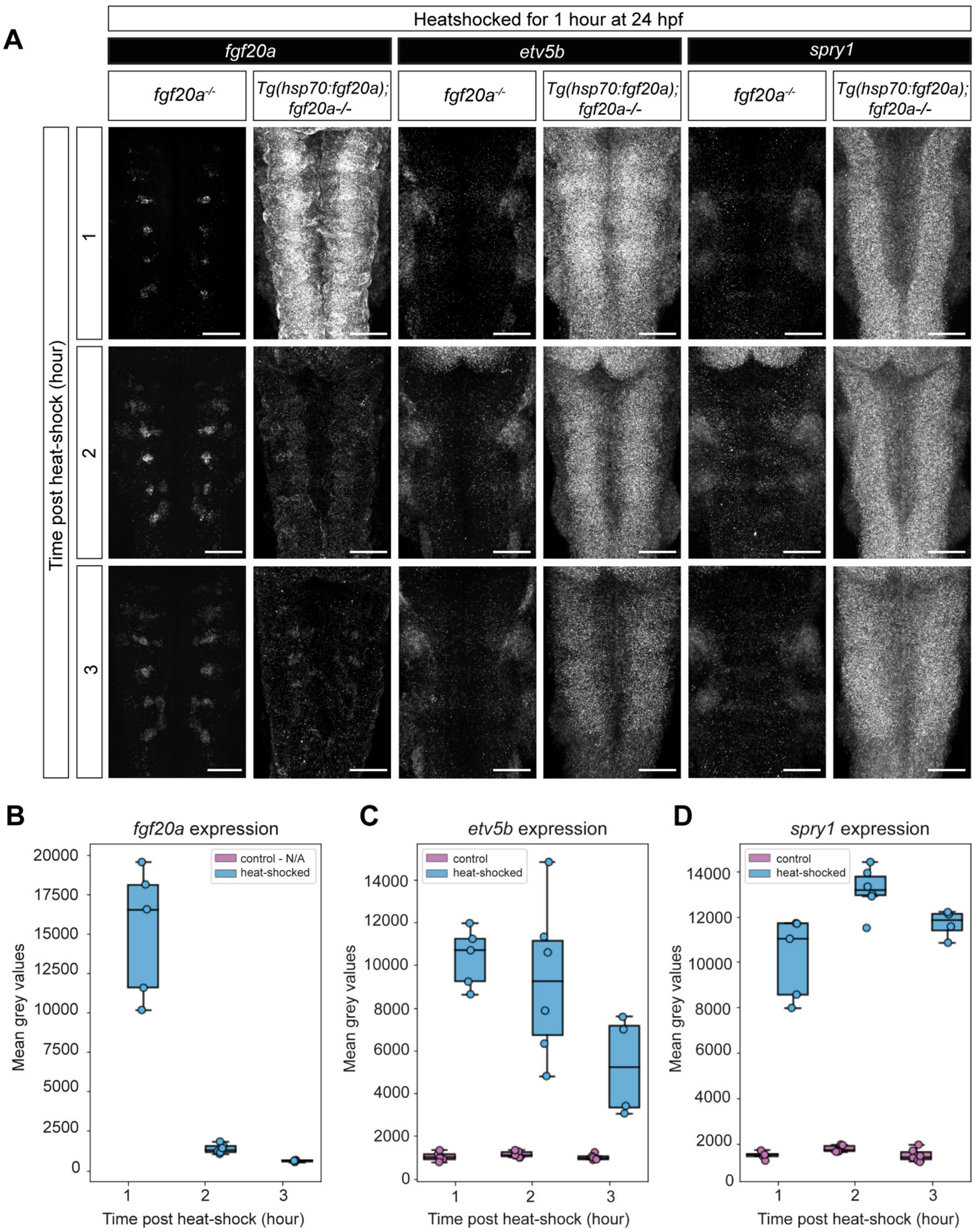

Fig. S6. Dynamics of *fgf20a* overexpression and Fgfr-MAPK pathway activation in *Tg(hsp70:fgf20a;fgf20a$^{-/-}$)* embryos.

(A) HCR RNA-FISH for *fgf20a, etv5b* and *spry1* in *fgf20a$^{-/-}$* and *Tg(hsp70:fgf20a;fgf20a$^{-/-}$)* embryos heat-shocked for 1 h at 24 hpf and fixed at 1 h, 2 h and 3 h post-heat-shock. Quantification of the expression of *fgf20a* (B), *etv5b* (C) and *spry1* (D) are shown in box plots. Four-channel confocal micrographs. Fgf20a transcripts are rapidly downregulated between 1 h and 2 h post-heat-shock. The dynamics of *etv5b* expression follow more closely to the dynamics of *fgf20a* expression than that of *spry1* expression. n≥5 per genotypes per conditions. Scale bar: 50 µm

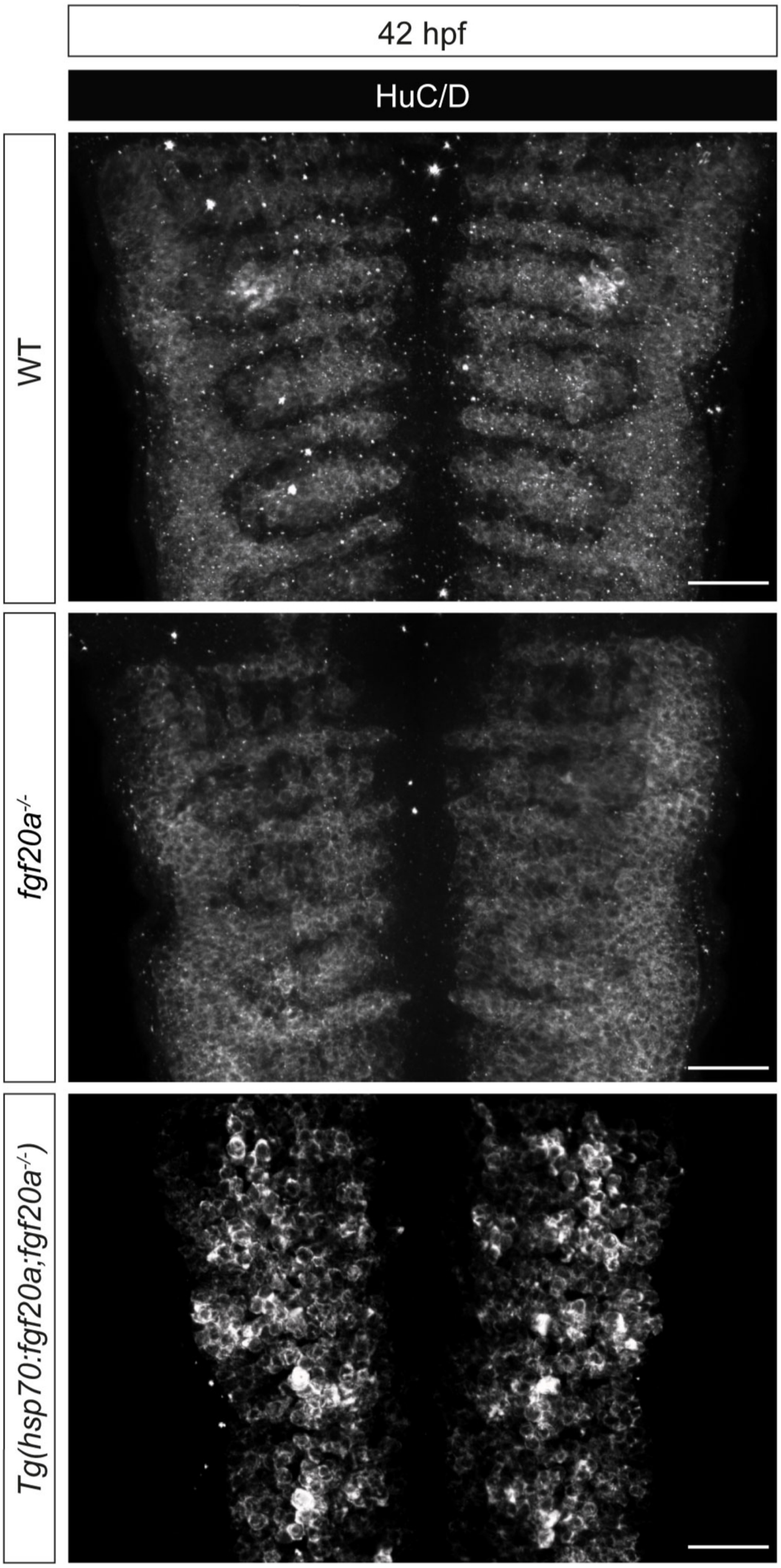

Fig. S7. Structural organisation of neurons in the mantle zone.

Immunostaining for HuC/D in WT (n=25), *fgf20a*$^{-/-}$ (n=17) and *Tg(hsp70:fgf20a;fgf20a*$^{-/-}$*)* (n=14) embryos at 42 hpf. *Tg(hsp70:fgf20a;fgf20a*$^{-/-}$*)* embryos were heat-shocked from 24- 30 hpf. Scale bar: 30 µm

Development • Supplementary information

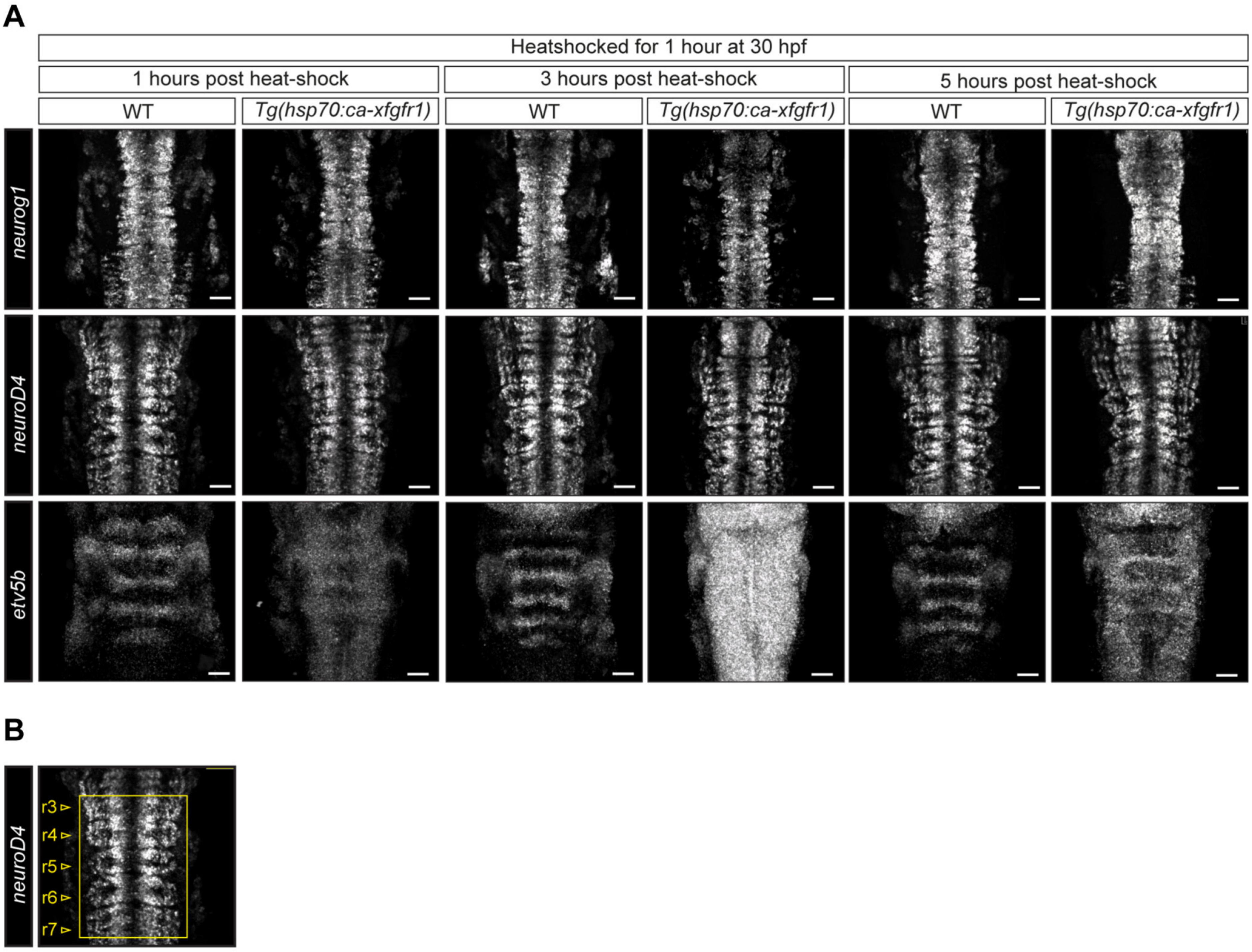

Fig. S8. Dynamics of *neuorg1* and *neuroD4* expression in response to Fgfr pathway activation.

(A) HCR RNA-FISH for *neurog1, neuroD4* and *etv5b* in WT and *Tg(hsp70:ca-fgfr1)* embryos heat-shocked for 1 h at 30 hpf and fixed at 1 h, 3 h and 5 h post-heat-shock. Four-channel confocal micrographs. n=4 (WT, 1 h); n=8 (*Tg(hsp70:ca-fgfr1)*, 1 h); n=5 (WT, 3 h); n=9 (*Tg(hsp70:ca-fgfr1)*, 3 h); n=5 (WT, 5 h); n=9 (*Tg(hsp70:ca-fgfr1)*, 5 h). Scale bar: 30 µm (B) The region of quantification of the dataset in (A) for the box plots in Fig. 3D-F.

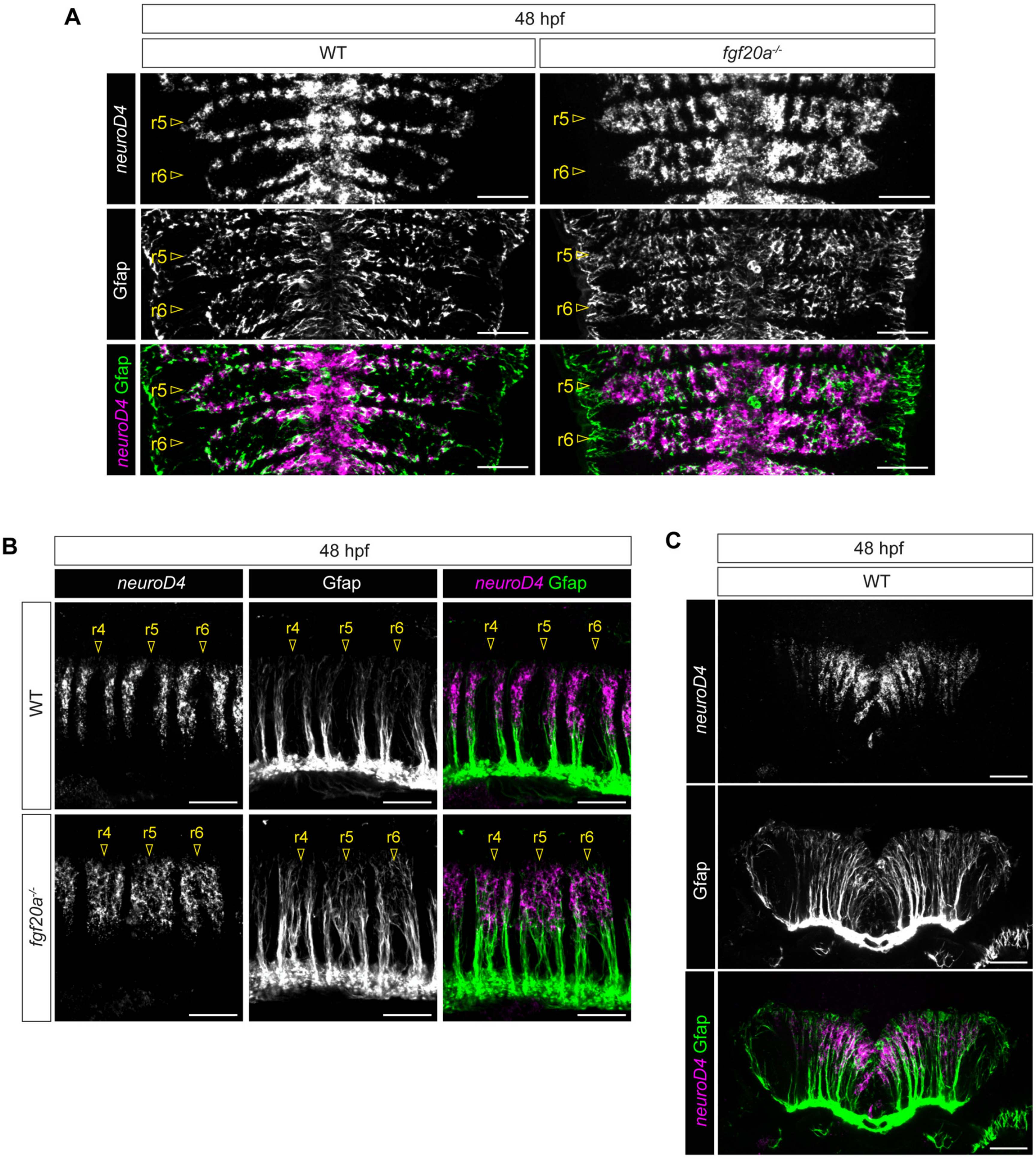

Fig. S9. *Gfap* colocalises with *neuroD4* in hindbrain progenitors.

(A) HCR RNA-FISH for gfap (green) and *neuroD4* (magenta) in WT (n=12) and *fgf20a*$^{-/-}$ embryos (n=12) at 48 hpf. Three-channel confocal micrographs. Dorsal view of r5 and r6. *Gfap* is colocalised with *neuroD4,* suggesting *gfap* is upregulated in neural progenitors committed to neuronal differentiation.

(B) Lateral sections of (A). Section thickness: 10 µm. (C) Transverse sections of WT of (A) at r5 region. Section thickness: 7.6 µm. Scale bar: 30 µm

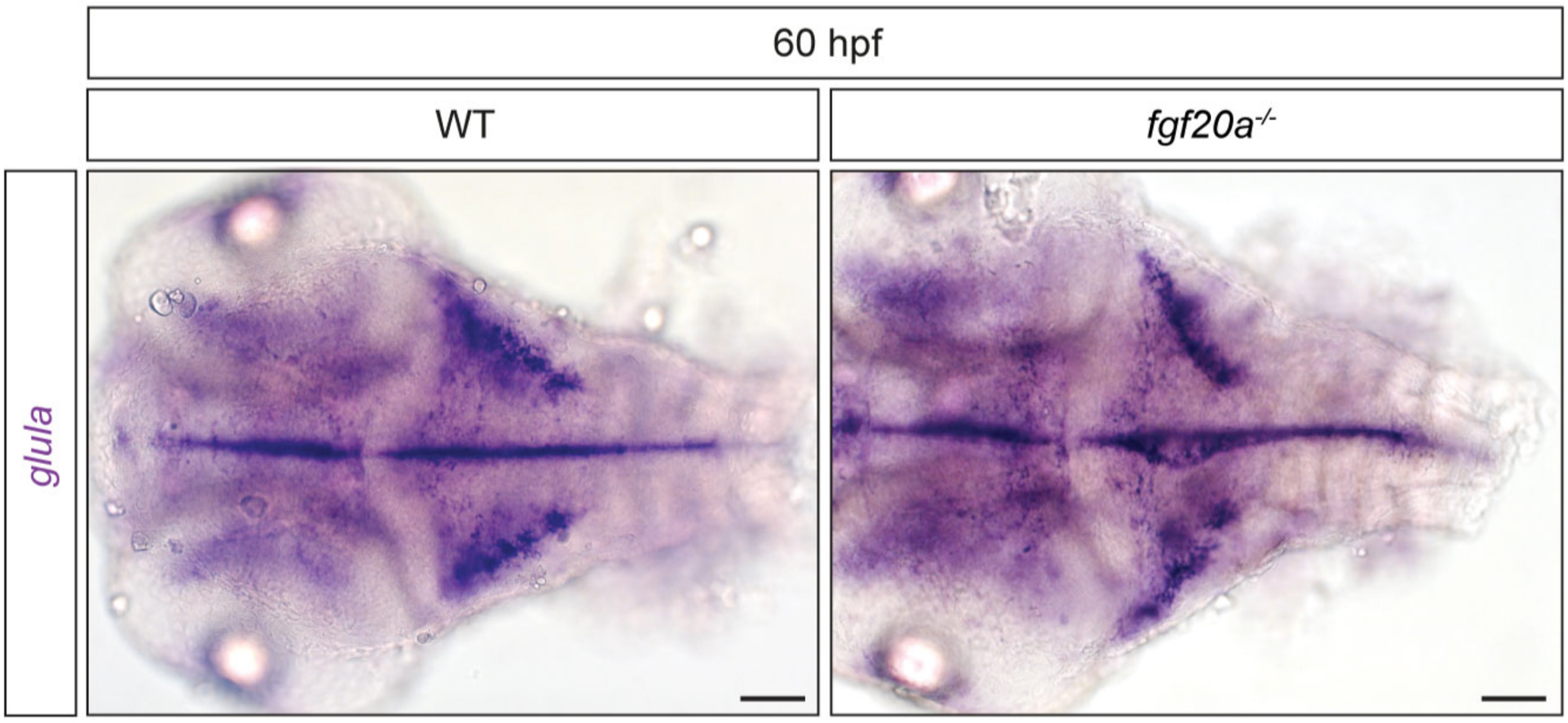

Fig. S10. Glula+ glial cells are unaltered in *fgf20a*$^{-/-}$. BCIP-NBT for *glula* in WT and *fgf20a*$^{-/-}$embryos at 60 hpf. n=12 per genotype. Scale bar: 50 µm

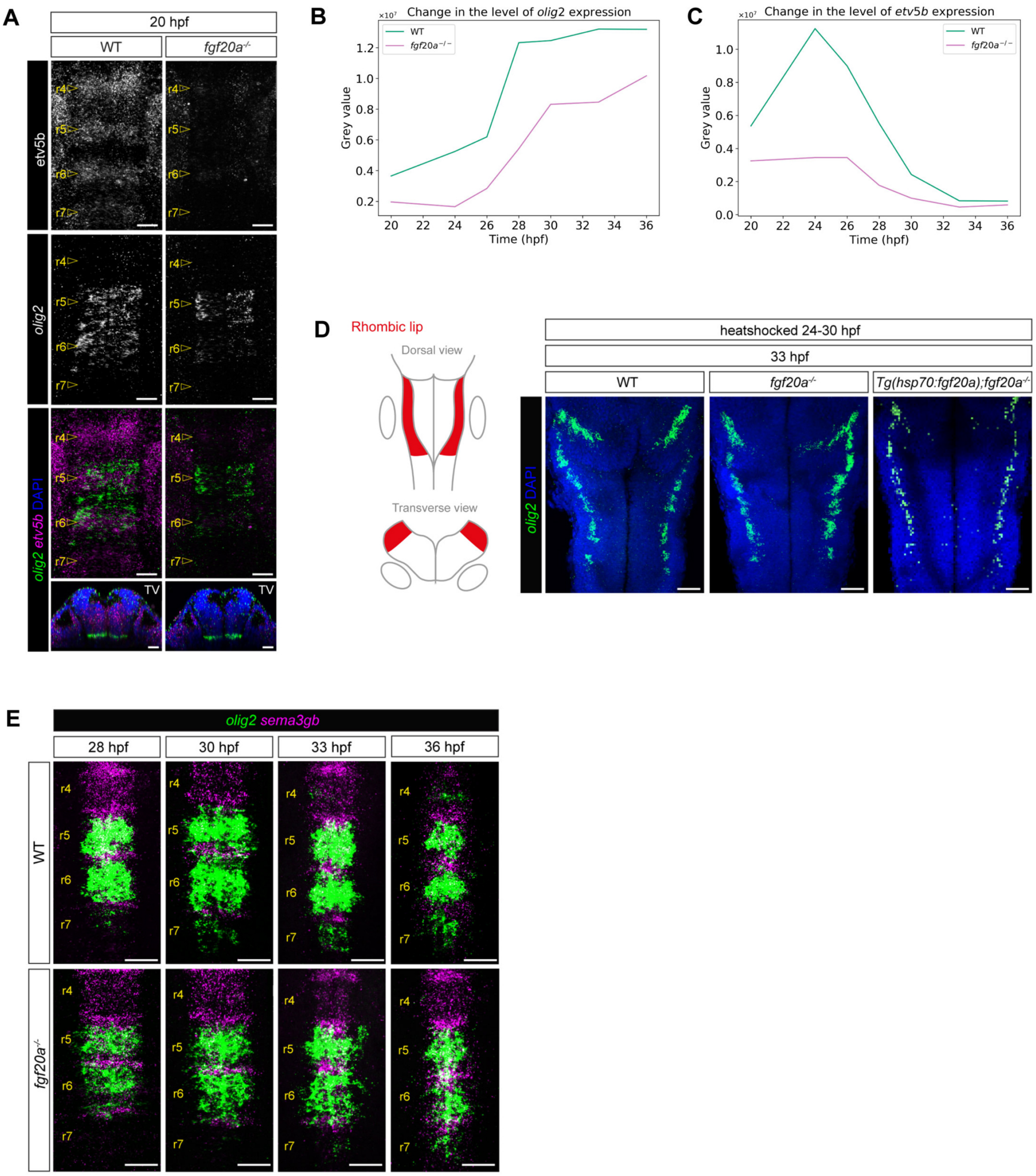

Fig. S11. *Olig2* expression in the ventral hindbrain is modulated by Fgf20 signalling. (A) HCR RNA-FISH for *olig2* (green) and *etv5b* (magenta) in WT and *fgf20a*$^{-/-}$ embryos at 20 hpf. DAPI (blue). Four-channel confocal micrograph. Dorsal view across r4 to r7. Dotted lines indicate the boundary between the ventricular zone and the mantle zone. Scale bar: 20 µm (B) Line plot of Fig. 4D.

(C) Line plot of Fig. 4E.

(D) HCR RNA-FISH for *olig2* (green) in WT, *fgf20a*$^{-/-}$ and *Tg(hsp70:fgf20a;fgf20a*$^{-/-}$*)* embryos at 33 hpf that were subjected to heat-shock from 24-30 hpf. DAPI (blue). Dorsal view of sections at the level of the rhombic lip only. *Olig2* expression in the rhombic lip (indicated in red in schematics) at the dorsal hindbrain is not regulated by Fgf20 signalling. n≥7 per genotype. Scale bar: 30 µm

(E) HCR RNA-FISH for *olig2* (green) and *sema3gb* (magenta) in WT and *fgf20a*$^{-/-}$ embryos at stages from 28-36 hpf. Dorsal view across r4 to r7. The hindbrain boundary marker *sema3gb* demarcates the rhombomeres. n≥6 per stage per genotype. Scale bar: 30 µm

Development • Supplementary information

Supp. Fig. 12

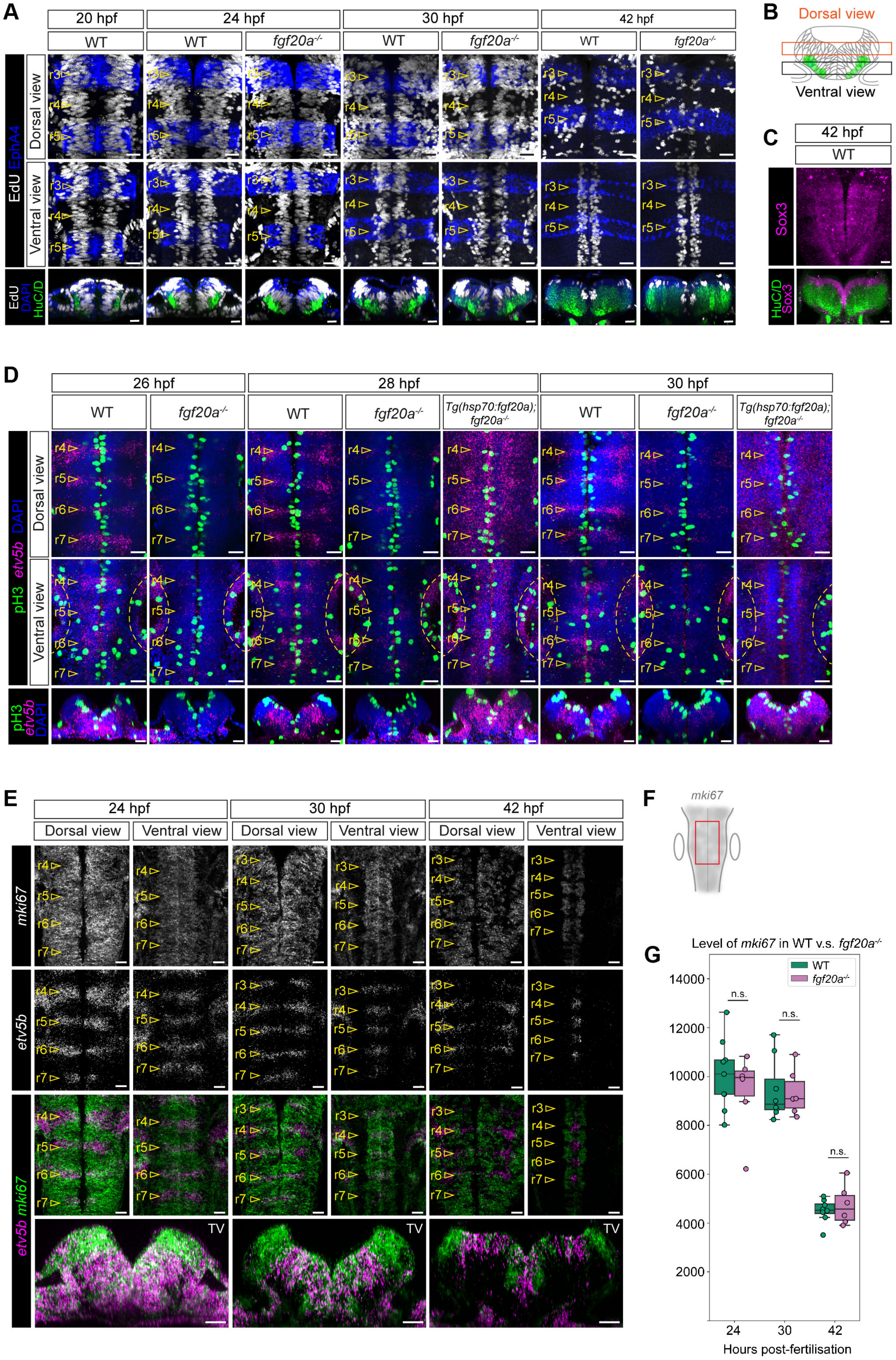

Development • Supplementary information

Fig. S12. Proliferation in the hindbrain is not patterned along the AP-axis nor influenced by Fgf20 signalling.

(A) EdU assay of the hindbrain. Triple-staining for EdU (grey), immunostaining for r3/r5 marker – EphA4 (blue), and immunostaining for HuC/D, in WT and *fgf20a*$^{-/-}$ embryos at stages across 20-42 hpf. Three-channel confocal micrographs. Dorsal view, ventral view and transverse view of 10-15 µm are shown. n≥20 per stage per genotype.

(B) Schematic of a transverse view of the hindbrain, indicating the position of the dorsal view and ventral view in (A), (D) and (E).

(C) Transverse section of double-immunostaining for the progenitor marker – Sox3
(magenta) and HuC/D (green) in WT at 42 hpf. n=12.

(D) pH3 staining in the hindbrain. Double-staining of HCR RNA-FISH for *etv5b* (magenta) and immunostaining for pH3 (green) in WT, *fgf20a*$^{-/-}$ and *Tg(hsp70:fgf20a;fgf20a*$^{-/-}$*)* embryos at 26, 28 and 30 hpf. DAPI (blue). Tg(hsp70:fgf20a;fgf20a-/-) embryos at 28 hpf were heat shocked for 1 h at 26 hpf.Tg(hsp70:fgf20a;fgf20a-/-) embryos at 30 hpf were heat shocked twice from 24 hpf to 28 hpf. Dotted lines demarcate the expression in the otic vesicles.
n=12 per stage per genotype. Scale bar: 20 µm.

(E) *Mki67* expression in the hindbrain. HCR RNA-FISH for *mki67* (green) and *etv5b* (magenta) in WT embryos at 24, 30 and 42 hpf. n=12 per stage.

(F) Schematic for the region of quantification of the dataset in (E) for the box plot in (G). The quantification was taken from a wholemount sum projection.

(G) Box plot showing quantification of (E). Statistical significance was determined using Mann–Whitney U test. n.s. = not significant, p>0.05. Scale bar: 20 µm.

Development • Supplementary information

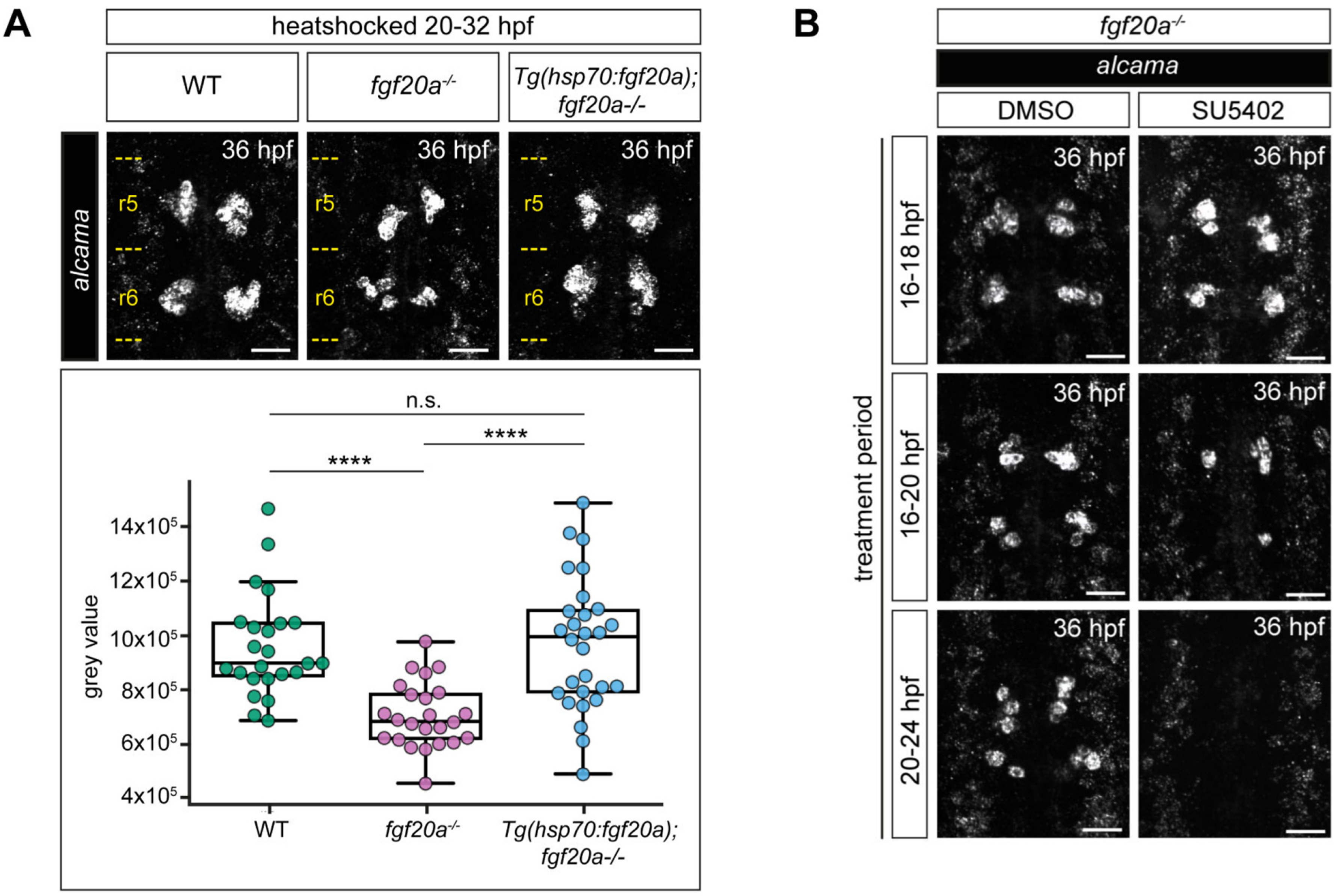

Fig. S13. Fgf20 signalling has little influence on *alcama+* motor neuron production.

(A) HCR RNA-FISH for *alcama* in WT, *fgf20a$^{-/-}$* and *Tg(hsp70:fgf20a;fgf20a$^{-/-}$)* embryos at 36 hpf, heat-shocked between 20-32 hpf. Quantifications of the expression are shown in the box plot. Each data point represents the sum grey value of an *alcama+* neuronal cluster. The production of *alcama+* neurons is slightly compromised in *fgf20a$^{-/-}$*, showing a small but significant reduction in quantity compared to that of WT embryos. However, the overexpression of Fgf20 does not result in significant increase in the quantity of *alcama+* neurons, suggesting that Fgf20 signalling has little effect on the production of *alcama+* motor neurons. n=6 (imaged) for each genotype. Statistical significance was determined using Mann–Whitney U test. n.s. = not significant, p>0.05; ****= P≤ 0.0001.

(B) HCR RNA-FISH for *alcama* in *fgf20a$^{-/-}$* embryos at 36 hpf, treated with SU5402 (6 µM) from 16-18 hpf, 16-20 hpf and 20-24 hpf. DMSO was used in the control embryos. An unknown source of Fgf signalling between 20-24 hpf is critical for the production of *alcama+* motor neurons. n≥12 per conditions. Scale bar: 15 µm.

Development • Supplementary information

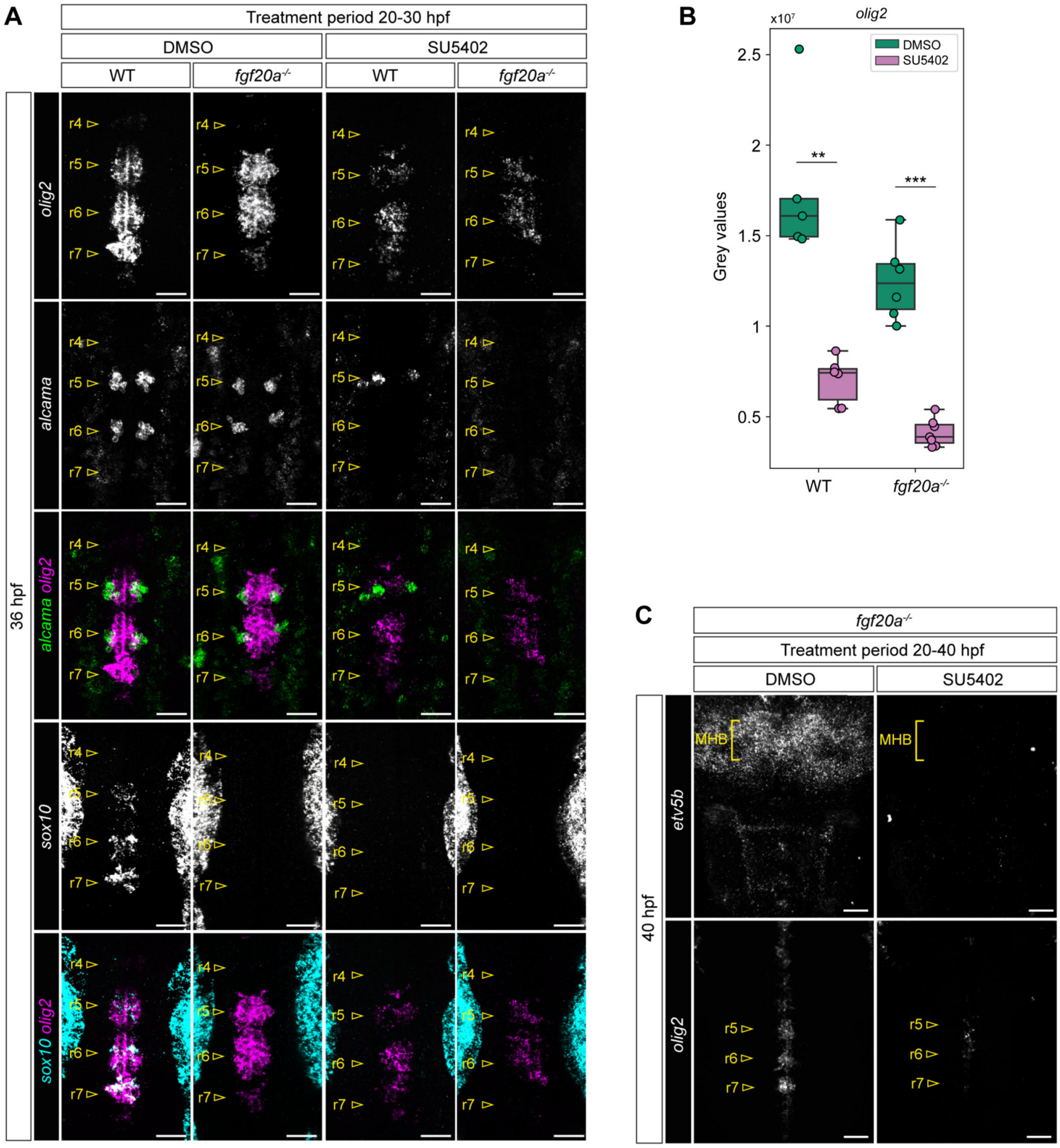

Fig. S14. The effects of pharmacologic inhibition of Fgfr pathway on *olig2*, *sox10* and *alcama+* motor neurons.
(A) HCR RNA-FISH for *olig2* (magenta), *alcama* (green) and *sox10* (cyan) in WT and *fgf20a*$^{-/-}$ embryos at 36 hpf, treated with SU5402 (10µM) from 20-30 hpf. DMSO was used in the control embryos. SU5402 treatment significantly inhibits the upregulation of *olig2* in both WT and *fgf20a*$^{-/-}$ embryos. Given that the SU5402 treatment further inhibits *olig2* expression in *fgf20a*$^{-/-}$, this suggests that unknown source of Fgf signalling and Fgfr pathway activation are required for the *olig2* upregulation observed in *fgf20a*$^{-/-}$. In WT embryos, Fgf20 signalling between 30-36 hpf post SU5402 treatment leads to a mild recovery in *alcama+* motor neuron production in r5 (66% of embryos) but no recovery in *sox10* expression. Taken together, this suggests that Fgf20 signalling has little contribution to *alcama+* motor neuron production and is essential for the timely specification of *sox10+* OPCs. n≥12 per genotype per conditions. Scale bar: 20 µm
(B) Box plot showing quantification of *olig2* expression in (A). The sum grey value of the *olig2* expression across r4 to r7 in the ventral hindbrain was measured. n=4 (WT, DMSO); n=6 (WT, SU5402); n=6 (*fgf20a*$^{-/-}$, DMSO); n=7 (*fgf20a*$^{-/-}$, SU5402). Statistical significance was determined using Mann–Whitney U test. n **= p≤0.01, ***= p≤0.001.
(C) HCR RNA-FISH for *olig2* and *etv5b* in *fgf20a*$^{-/-}$ at 40 hpf, treated with SU5402 (10 µM) from 20-40 hpf. SU5402 treatment abolishes Fgfr pathway activity as indicated by the loss of *etv5b* expression at the midbrain-hindbrain boundary (MHB) where there is a high level of Fgf8 signalling. The initial *olig2* expression in the SU5402-treated embryos is maintained. This suggests that Fgfr pathway activity is not required for sustaining *olig2* expression. n≥12 per condition. Scale bar: 30 µm

Development • Supplementary information

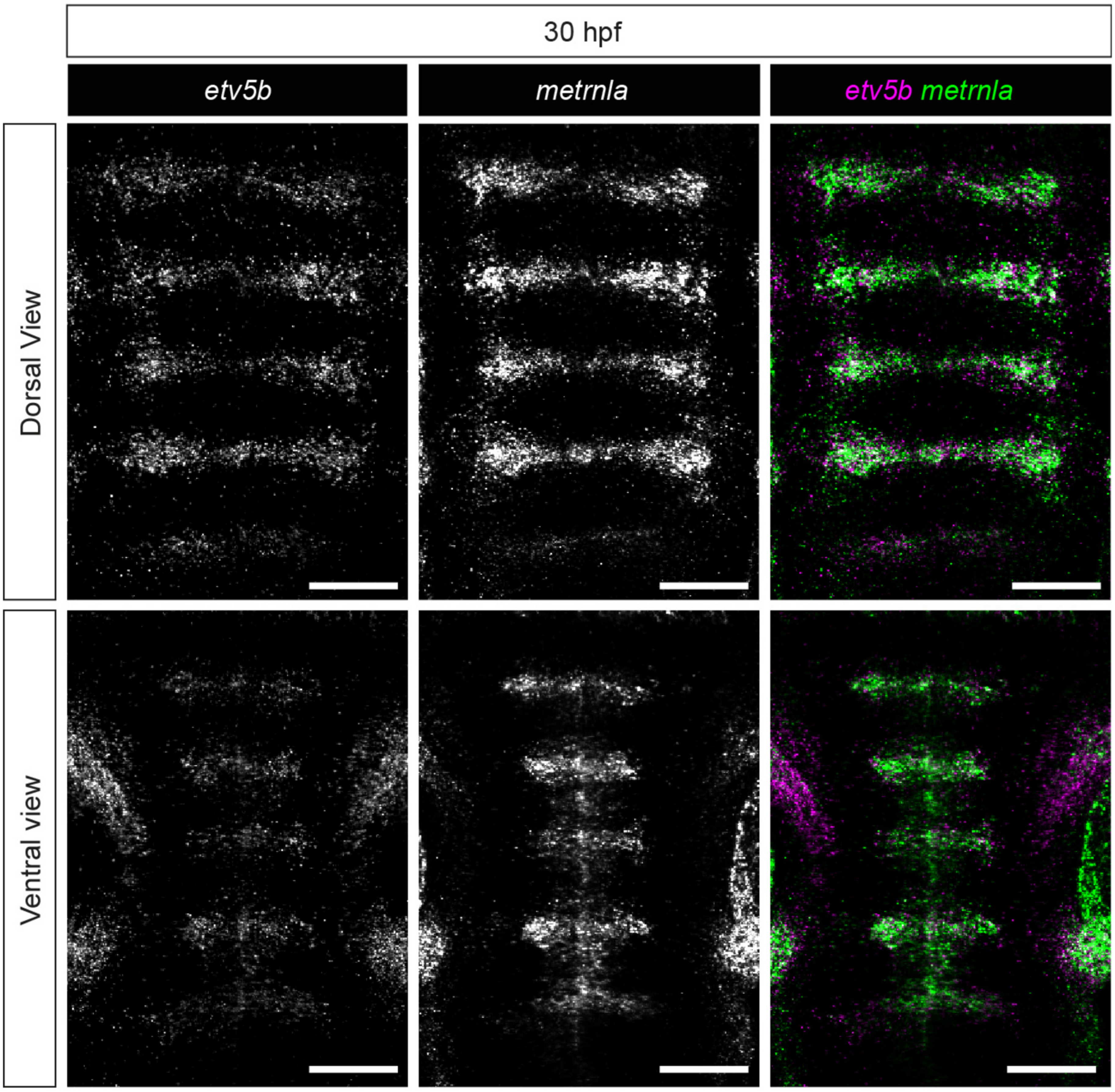

Fig. S15. Coexpression of etv5b and metrnla in wild type embryos.
Multiplexed HCR for *etv5b* (magenta) and *metrnla* (green) in wild type embryos at 30 hpf. Showing hindbrain segments from r3-r7. Dorsal views and ventral views are sections of 20 µm. n≥12 per condition. Scale bar: 30 µm

Development • Supplementary information
