# Supplementary material for: Short-range Fgf signalling patterns hindbrain progenitors to induce the neurogenesis-to-oligodendrogenesis switch
Source: Development. 2024 Dec 13;151(24):dev204256. doi: 10.1242/dev.204256 (PMC11664172; doi:10.1242/dev.204256)
Supplement: Supplementary file 1 [file develop-151-204256_review_history.pdf]

Short-range Fgf signalling patterns hindbrain progenitors to induce the neurogenesis-to-oligodendrogenesis switch

Tim J. Yeung and David G. Wilkinson
DOI: 10.1242/dev.204256

Editor: Steve Wilson

Review timeline

Original submission:	17 July 2024
Editorial decision:	28 August 2024
First revision received:	10 October 2024
Accepted:	14 November 2024

Original submission

First decision letter

MS ID#: DEVELOP/2024/204256

MS TITLE: Short-range Fgf signalling patterns hindbrain progenitors to induce the neurogenesis-to-oligodendrogenesis switch

AUTHORS: David G. Wilkinson; Tim Justina Yeung

I have now received all the referees' reports on the above manuscript, and have reached a decision. The referees' comments are appended below, or you can access them online: please go to:

As you will see, the referees express considerable interest in your work, but have some suggestions for improvements. If you are able to revise the manuscript along the lines suggested, I will be happy receive a revised version of the manuscript. If it would be helpful, you are welcome to contact us to discuss your revision in greater detail. Please send us a point-by-point response indicating your plans for addressing the referees’ comments, and we will look over this and provide further guidance.

Reviewer 1

Advance summary and potential significance to field

In this paper the authors present evidence for local Fgf signalling provided by earlier born neurons in the regulation of the switch from neurogenesis to gliogenesis in the developing zebrafish hindbrain. A well-defined series of gene expression analyses reveal neuronal expression of Fgf20 accompanied by FGFR signalling target gene expression in neighbouring neural progenitor cells. Using multiple approaches to increase or reduce Fgf20/Fgf signalling, they demonstrate that such signalling mediates a switch from neurogenesis to gliogenesis, involving downregulation of early proneural genes and induction of oligodendrocyte differentiation factors. This paper is very well written, quantitative data are clearly and consistently provided in the figure legends and the experiments are well executed. Overall, these data support a model in which neurons locally regulate the specification of remaining neural progenitors, mediating the switch from neurogenesis to gliogenesis. A related observation has been reported in the developing mouse cortex, where later born neurons upregulate Fgf9 expression, which can promote gliogenesis, but the role of FGF

© 2024. Published by The Company of Biologists under the terms of the Creative Commons Attribution License (https://creativecommons.org/licenses/by/4.0/).

signalling levels in controlling the patterning, timing and quantity of glial cells is uniquely explored in this new paper.

The suggestions for the authors are largely for clarification and additional information and may help to develop interpretation of their data and the Discussion.

Comments for the author

i) Given the expression of ERK1/2 target Etv5 in neural progenitors about to become gliogenic, it would be predicted that an ERK1/2 or MEK inhibitor would have the same impact as the FGFR inhibitor SU5402. Can the authors comment on this potential experiment, which could rule out roles for other FGFR downstream pathways in the switch from neurogenesis to gliogenesis.

ii) The authors present evidence that supports FGF20 ligand acting locally at the basal side neural progenitor cells (Figs 1D, E). Given interkinetic nuclear migration characteristic of the proliferative neuroepithelium could it be that when the nucleus is located basally it presents a greater surface area for ligand-receptor binding and that this "basal visiting" achieves sufficient levels of signalling to direct OPC specification? This would be an interesting possibility as such cells would be in G1 and about to enter S-phase of the cell cycle and so well placed to alter cell fate as they undergo DNA replication.

iii) Can the authors clarify how Fgf20 signalling leads to induction of gliogenesis genes. Onset of Olig2 expression is FGFR independent, but maintenance relies on Fgf20, with Fgf20 KO leading to half the number of Olig2 expressing cells and Fgf20 over-expression generating twice as many as wildtype this seems like regulation of proliferation of an existing Olig2 expressing cell-population. And yet further experiments indicate no effect on proliferation. The conclusion seems to be that levels of FGF signalling are important - can this be supported further? For example, distinct threshold levels of FGF signalling induce specific combinations of PEA3 family genes (PMID: 11413000) with Etv5 (Erm) induced by low level and Etv4 (Pea3) higher levels. Is the expression pattern of Etv4 known in the zebrafish hindbrain? Does it increase at gliogenesis and after initial Etv5 expression?

iv) Any ideas for what the compensatory mechanism might be in other hindbrain cell populations? Is Fgf20b upregulated in the Fgf20a KO?

v) Can the authors comment on what might regulate the onset of Fgf20 in newborn neurons - Fgf20 expressing neurons accumulate over time from 16hpf, and locally induce Etv5. Is Fgf20 expression prefigured by other factors such as Sip1 or a related gene, (which acts in a similar context in mouse cortical neurons to restrain upregulation of Fgf9 PMID: 19838179)?

Reviewer 2

Advance summary and potential significance to field

A hallmark feature of neural development is the neuron-glia switch, whereby neurogenesis generally precedes gliogenesis. Because the timing of the switch can affect the numbers and types of neurons and glia that are produced, the mechanisms that control the switch are critically important. One model that has emerged to explain the switch is that subpopulations of neurons produce signals to terminate neurogenesis and initiate gliogenesis. The authors test this model using the zebrafish hindbrain as an experimental model. The main finding of the study is that Fgf20a, produced by small groups of early-born neurons, triggers a switch to oligodendrocyte precursor cell (OPC) production in spatially defined regions of the hindbrain.

The manuscript is nicely written, the figures are informative and well-organized, and most of the conclusions and claims made by the authors are sufficiently supported by the data. The information presented here is new and will likely be an important contribution to the field. Our recommendations to the authors are relatively minor.

Comments for the author

© 2024. Published by The Company of Biologists under the terms of the Creative Commons Attribution License (https://creativecommons.org/licenses/by/4.0/).

1. There are some important details missing from the methods. First, the specific alleles of the mutant genes used in this study need to be identified. Second, there needs to be a description of how embryo genotypes were determined. Were they produced by crosses of homozygous viable adults so the genotypes were known? If they were produced by crosses of heterozygous adults, were embryos sorted by morphological criteria or were they genotyped using a DNA-based method? Third, what methods were used, such as blinding, to reduce bias in the data collection and analysis?

2. The heading for Figure 1, "Short-range Fgf20 signaling", is quite subjective and these images don't really support that point. The difference in etv5b expression shown in panel D makes it appear that Fgf20 signaling reaches across the dorsoventral extent of the hindbrain. The emphasis on short range signaling from these data seems a bit misleading.

3. In figure 1 panels D and E are switched.

4. In Figure 5 the authors show that transgenic overexpression of Fgf20a promotes formation of excess OPCs and a thinner hindbrain mantle zone, with apparently fewer neurons. They use these data to support the statement on p. 9, "suggesting that Fgf20 promotes OPCs at the expense of neuronal production." This seems like a reasonable idea but there are no data to support it, and we would encourage the authors to consider a more nuanced interpretation. The main idea behind the authors' model, we think, is that neurons and OPCs arise from common progenitors through some sort of binary switch. Indeed, the paper by Zannino and Appel (2009) shows that alcama+ motor neurons and OPCs arise from a common population of olig2+ precursors. Thus, one possible prediction from the authors' model is that Fgf20 inhibits alcama+ motor neuron production. However, that is not what they find. In fact, mutant embryos have fewer alcama+ motor neurons and overexpression does not block their formation. Thus, the neuron populations affected by the overexpression experiment are not alcama+ motor neurons. An alternative explanation is that Fgf20a overexpression affects neurons and OPCs independently. Sorting out these distinctions would require lineage tracing. We do not think that it is necessary to do those experiments for this paper, but it would be helpful for the authors to more fully consider this point.

5. For experiments described in Figure 6, the authors use OPC "delamination" as an indicator of differentiation state and indicate that one of their manipulations does not result in delamination. This is a pretty weak and not very informative point. First, the images are not of sufficient magnification and resolution to assess delamination. Second, we are not convinced that it is a very meaningful indicator of differentiation or that it provides much insight to the experimental result.

6. The legend for Figure 6 states, "Fgf20 signaling upregulates olig2 and sox10 expression in competent progenitors". In text associated with Figure 3 the authors state that "all hindbrain neural progenitors are competent to respond to fgf20". Because olig2 and sox10 upregulation are restricted, this appears to be two different uses of the term "competent". It might be helpful for the authors to clarify what they mean by progenitors competent to upregulate olig2 and sox10 in response to Fgf20.

7. In the first paragraph of the discussion section, the authors state that "fgf20-expressing neuronal cluster pattern progenitors spatially and temporally" and that fgf20 induces "oligodendrogenesis among competent cells in that region through upregulation of OPC specification factors, olig2 and sox9, and differentiation factor, sox10". However, the authors do not show sox9 expression data.

<u>Reviewer 3</u>

Advance summary and potential significance to field

In this manuscript, the authors study the role of Fgf20 in spatially and temporally patterning progenitor cells during Zebrafish hindbrain development. The authors find that Fgf20 is expressed by early born neurons starting at 16 hpf, and that this leads to activation of target genes Etv5b and Spry only in neighboring progenitor cells by 20 hpf. Ubiquitous overexpression of Fgf20 can result in

© 2024. Published by The Company of Biologists under the terms of the Creative Commons Attribution License (https://creativecommons.org/licenses/by/4.0/).

broad expression of Etv5b, suggesting that all progenitors are capable of responding to Fgf20. They also show that at later time points (30-36 hpf) Fgf20 is required for repressing neural progenitor genes (Neurog1/2), and activating oligodendrocyte progenitor genes (Olig2/Sox10) at the segment centre. Loss of function in fgf20 knockouts leads to an increase in Nerog1/2 expression in segment center between 30-36 hpf, and a reduction of Olig2/Sox10 expression, while ubiquitous overexpression of Fgf20 has the inverse effect. All together, the authors conclude that Fgf20 signaling from early born neurons acts at short range to induce the neurogenesis-to-gliogenesis switch.

The model proposed by the authors- that newborn neurons can spatially and temporally regulate the developmental potential of progenitor cells- could potentially be an exciting advance in the field. Such a mechanism would be an elegant way to control the number of neurons and glia that are generated in different segments across a developing tissue. However, some aspects of this model, especially in regards to temporal control, are either not fully supported by data, or not well explained.

Comments for the author

Detailed comments with suggested experiments for revisions are below:

1. The Results section of the manuscript is difficult to follow. A schematic that shows the relevant structures in the developing hindbrain over the relevant time span would be helpful. Including a developmental timeline of important events - when fgf20 is activated, when proneural genes are downregulated, when OPC genes are activated - would also make the results more clear/easier to follow along.

2. In Figure 1, the authors arrive to the conclusion that Fgf20 works at a short range, rather than as a gradient. This is based on two pieces of evidence: i) Fgf20 target genes, Etv5b and Spry1 are activated only in progenitors right next to the Fgf20 expressing neurons in wt conditions. ii) When Fgf20 is ubiquitously overexpressed with heatshock, it can activate ubiquitous expression of Etv5b in all progenitors. Therefore, the authors conclude that while all progenitors are competent to respond to Fgf20, only the cells next to Fgf20 do so because Fgf20 is a weak signal that acts at a short range.
However, another interpretation of these results is that Etv5b and Spry1 are targets that require high Fgf20 to get activated. The Shh gradient in chick/mouse spinal cords activates some targets at high concentrations and other targets at low concentrations. What is the evidence that Fgf20 cannot activate other targets at low levels, farther away from the source?

3. The authors hypothesize that Fgf20 homodimerization could be contributing to its short-range activity. Could the authors perform western blots to show that Fgf20 in the hindbrain (or whole animal if amount of tissue is limiting) is present as a homodimer?

4. In Figure 3, it would be helpful to see the wildtype levels of Etv5 also quantified in A and E, because of point 6.

5. A clarification of Etv5b function in progenitors is needed. It seems that Etv5b is expressed during neurogenesis but not during gliogenesis (expression goes down over time between 24-36 hpf in Figure 4). Yet, overexpressing Fgf20, which should suppress neurogenesis and induce gliogenesis, results in upregulation of Etv5b at all timepoints where this data is shown. It is unclear if this fits the authors' model or if this is not relevant to the model for reasons not explained.

6. In Figure 3, the authors claim that the magnitude of proneual gene downregulation is dependent on the level of Fgf20. There are two points about this that are unclear:

a. The cellular resolution is unclear because of the nature of RNA-FISH data. It is therefore difficult to interpret if the authors mean that in any given cell, higher levels of Fgf20 = higher downregulation of proneural genes in surrounding cells. Or do they mean that when Fgf20 is expressed in more cells there is overall more downregulation of proneural genes in the hindbrain. If the second, this does not necessarily mean that the level of Fgf20 is what controls downregulation. If this experiment is possible, one way to test the levels claim could be to express Fgf20 at higher

© 2024. Published by The Company of Biologists under the terms of the Creative Commons Attribution License (https://creativecommons.org/licenses/by/4.0/).

levels in the newborn neurons using a targeted approach and then test whether there is earlier and greater reduction of proneural genes in the segment centre/surrounding region.

b. There is also temporal variability in many of these experiments, that confound interpretations. Do the authors think that high level of Fgf20 signaling is sufficient to reduce proneuronal gene expression? Fgf20 is expressed starting at 16 hpf, but proneurnal genes are excluded from the segment center ~30-36 hpf (double FISH for Fgf20a and proneural genes over this time course could be helpful). Is this because an increase in Fgf20 signal during this time window is necessary for reaching a threshold that is sufficient to suppress pro-neural genes at 36 hpf? Or is there another time-dependent factor that primes the progenitors, so that Fgf20 can downregulate proneural genes between 30 hpf and 36 hpf? This could be tested by inducing high expression of Fgf20 before 30 hpf (maybe around 20 hpf) to see if higher levels are sufficient to downregulate proneural genes at earlier time points (such as 24 hpf). All heatshock experiments in Fig 3 are done at later timepoints, making it difficult to distinguish between the two possibilities mentioned here.

7. Why is the timing of heatshock different in Fig 3A, Fig 3E, and supplemental figures 6, 8. These experiments cumulatively show i) timing of peak Fgfr/Fgf20 activity after heatshock, and ii) how this effects expression of proneural genes. Keeping the timing consistent seems important as the developmental process being studied is temporally controlled.

8. How do the authors explain the difference in timing of peak Etv5b activation after heatshock induction of Fgfr (3 hours) vs. Fgf20 (1hr)? supplemental figure 6 vs. supplemental figure 8/figure 3.

9. Related to point 7: Figure 5A shows that ubiquitously inducing Fgf20a from 24-30 hpf leads to precocious expression of Sox10 at 32 hpf. Does this also lead to precocious reduction of Neurog? Does this result in reduction of neurons and increase in oligodendrocytes?

10. Overall, the temporal nature of Fgf20 function is not clear. Clarifying this point by inducing heatshock-Fgf20 at one early and one late timepoint, and testing multiple outcomes after each heatshock: Etv5b, Neurog, Olig2/Sox10 expression, panneuronal, oligodendrocyte markers would help clarify the authors' model.

If the author's model is that levels of Fgf20 determine when and where the neurogenic to gliogenic switch happens, then Fgf20 should be sufficient to drive this transition even at early timepoints upon overexpression. However, if overexpression of Fgf20 at early timepoints is not sufficient to drive the switch, this could suggest that the temporal control of the switch is dependent on other factors. In that case, Fgf20 is required for the switch and controls where the switch happens, but does not necessarily control when it happens.

First revision

Author response to reviewers' comments

We thank the reviewers for their helpful comments. We have made changes to the manuscript and figures to address the points that have been raised, in particular to clarify the presentation and extend the interpretations. These changes have significantly improved the manuscript.

Reviewer 1: SUMMARY OF THE ADVANCE MADE IN THIS PAPER AND ITS POTENTIAL SIGNIFICANCE TO THE FIELD
In this paper the authors present evidence for local Fgf signalling provided by earlier born neurons in the regulation of the switch from neurogenesis to gliogenesis in the developing zebrafish hindbrain. A well-defined series of gene expression analyses reveal neuronal expression of Fgf20 accompanied by FGFR signalling target gene expression in neighbouring neural progenitor cells. Using multiple approaches to increase or reduce Fgf20/Fgf signalling, they demonstrate that such signalling mediates a switch from neurogenesis to gliogenesis, involving downregulation of early proneural genes and induction of oligodendrocyte differentiation factors. This paper is very well

© 2024. Published by The Company of Biologists under the terms of the Creative Commons Attribution License (https://creativecommons.org/licenses/by/4.0/).

written, quantitative data are clearly and consistently provided in the figure legends and the experiments are well executed. Overall, these data support a model in which neurons locally regulate the specification of remaining neural progenitors, mediating the switch from neurogenesis to gliogenesis. A related observation has been reported in the developing mouse cortex, where later born neurons upregulate Fgf9 expression, which can promote gliogenesis, but the role of FGF signalling levels in controlling the patterning, timing and quantity of glial cells is uniquely explored in this new paper.

The suggestions for the authors are largely for clarification and additional information and may help to develop interpretation of their data and the Discussion.

SUGGESTIONS TO AUTHORS

i) Given the expression of ERK1/2 target Etv5 in neural progenitors about to become gliogenic, it would be predicted that an ERK1/2 or MEK inhibitor would have the same impact as the FGFR inhibitor SU5402. Can the authors comment on this potential experiment, which could rule out roles for other FGFR downstream pathways in the switch from neurogenesis to gliogenesis.

We agree that it would be interesting to narrow down the pathway responsible for the neurogenesis-to- gliogenesis switch downstream of Fgfr. We had attempted to visualise ERK activation in the hindbrain with P-Erk (Sigma, M8159) antibody but was not successful in the detection. Preliminary observations using a transgenic line with an improved ERK reporter (Wilcockson et al., 2023) had also been attempted. However, owing to the density and arrangement of the tissue, in combination with the nature of the reporter, we think that further optimisation of imaging techniques and analysis strategy will be required for the characterisation. Nonetheless, the MEK inhibitor PD-0325901 that is known to act well in zebrafish (Anastasaki et al., 2012) would be a good choice for probing the requirement of ERK. The current study serves as a solid groundwork for this, as the results reveal the temporal requirement of the pathway and the phenotypes expected under successful perturbations.
We now mention in the Discussion (p.14) the question of whether ERK1/2 are involved.

ii) The authors present evidence that supports FGF20 ligand acting locally at the basal side neural progenitor cells (Figs 1D, E). Given interkinetic nuclear migration characteristic of the proliferative neuroepithelium could it be that when the nucleus is located basally it presents a greater surface area for ligand-receptor binding and that this "basal visiting" achieves sufficient levels of signalling to direct OPC specification? This would be an interesting possibility as such cells would be in G1 and about to enter S-phase of the cell cycle and so well placed to alter cell fate as they undergo DNA replication.

This is an excellent point and we have added it to the discussion (p.12). Indeed, it has been described in the zebrafish retina (Del Bene et al., 2008) that interkinetic nuclear migration (INM) is crucial for modulating the rate of neurogenesis across the progenitor populations by bringing the cell bodies closer to or away from the apically located Notch signalling, which inhibits neurogenesis and thus maintain progenitors for later specifications. INM mediated signalling would be an attractive model in this context as it complements the short-range property of Fgf20 signalling. For instance, it would be interesting to investigate whether the speed/rate of INM of hindbrain progenitors influences the temporal dynamics of the logistic increase in olig2 expression (Fig. 4D, Supp Fig. 11B).

iii) Can the authors clarify how Fgf20 signalling leads to induction of gliogenesis genes. Onset of Olig2 expression is FGFR independent, but maintenance relies on Fgf20, with Fgf20 KO leading to half the number of Olig2 expressing cells and Fgf20 over-expression generating twice as many as wildtype this seems like regulation of proliferation of an existing Olig2 expressing cell-population. And yet further experiments indicate no effect on proliferation. The conclusion seems to be that levels of FGF signalling are important - can this be supported further? For example, distinct threshold levels of FGF signalling induce specific combinations of PEA3 family genes (PMID: 11413000) with Etv5 (Erm) induced by low level and Etv4 (Pea3) higher levels. Is the expression pattern of Etv4 known in the zebrafish hindbrain? Does it increase at gliogenesis and after initial

© 2024. Published by The Company of Biologists under the terms of the Creative Commons Attribution License (https://creativecommons.org/licenses/by/4.0/).

Etv5 expression?

As the reviewer points out, we showed that the amount of Fgf signalling has a significant impact on the level of olig2 expression in our context not via the indirect input of regulating proliferation. This is further highlighted by the results shown in Fig.6C,D where the overexpression of Fgf20 at a late stage (36 hpf - around 12 hours after the peak of Fgf20 signalling and 10 hours after the upregulation of olig2 in WT condition), leads to the upregulation of olig2 and sox10 among competent progenitors. The regulatory pathway downstream of fgfr acting on olig2 and sox10 expression is not explored in this study. Regarding the possibility of Fgf signalling acting through Etv4/Pea3 in this context, expression data in the ZFIN database indicate that etv4 is not expressed in the hindbrain during the relevant period.

iv) Any ideas for what the compensatory mechanism might be in other hindbrain cell populations? Is Fgf20b upregulated in the Fgf20a KO?

Unfortunately, we did not generate image datasets suitable for quantitative analysis to compare fgf20b expression between WT and fgf20a-/-. Nonetheless, regarding the possibility of Fgf20b compensating for Fgf20a, if there is any upregulation of the ligand at a later stage it does not induce a significant Fgfr pathway response based on the level of etv5b expression, as quantified in Fig. 4E. However, we did observe in fgf20a-/- ectopic upregulation of olig2 at the hindbrain boundaries by 36 hpf (Supp Fig. 11E). This points to a possibility that hindbrain boundary cells, which serve as stem cell pools, can contribute to the compensatory mechanism. We now comment on this (p.9).

v) Can the authors comment on what might regulate the onset of Fgf20 in newborn neurons - Fgf20 expressing neurons accumulate over time from 16hpf, and locally induce Etv5. Is Fgf20 expression prefigured by other factors such as Sip1 or a related gene, (which acts in a similar context in mouse cortical neurons to restrain upregulation of Fgf9 PMID: 19838179)?

We have not investigated the interesting question of potential regulators of fgf20 expression in neurons but Sip1 is a good candidate and we have added a comment regarding this in the Discussion (p.12).

Reviewer 2: SUMMARY OF THE ADVANCE MADE IN THIS PAPER AND ITS POTENTIAL SIGNIFICANCE TO THE FIELD
A hallmark feature of neural development is the neuron-glia switch, whereby neurogenesis generally precedes gliogenesis. Because the timing of the switch can affect the numbers and types of neurons and glia that are produced, the mechanisms that control the switch are critically important. One model that has emerged to explain the switch is that subpopulations of neurons produce signals to terminate neurogenesis and initiate gliogenesis. The authors test this model using the zebrafish hindbrain as an experimental model. The main finding of the study is that Fgf20a, produced by small groups of early-born neurons, triggers a switch to oligodendrocyte precursor cell (OPC) production in spatially defined regions of the hindbrain.

The manuscript is nicely written, the figures are informative and well-organized, and most of the conclusions and claims made by the authors are sufficiently supported by the data. The information presented here is new and will likely be an important contribution to the field. Our recommendations to the authors are relatively minor.

SUGGESTIONS TO AUTHORS
1. There are some important details missing from the methods. First, the specific alleles of the mutant genes used in this study need to be identified. Second, there needs to be a description of how embryo genotypes were determined. Were they produced by crosses of homozygous viable adults so the genotypes were known? If they were produced by crosses of heterozygous adults, were embryos sorted by morphological criteria or were they genotyped using a DNA-based method? Third, what methods were used, such as blinding, to reduce bias in the data collection and analysis?

© 2024. Published by The Company of Biologists under the terms of the Creative Commons Attribution License (https://creativecommons.org/licenses/by/4.0/).

Thank you for pointing this out. We have added details on the identification of mutants to the Methods section (p.22).

2. The heading for Figure 1, "Short-range Fgf20 signaling", is quite subjective and these images don't really support that point. The difference in etv5b expression shown in panel D makes it appear that Fgf20 signaling reaches across the dorsoventral extent of the hindbrain. The emphasis on short range signaling from these data seems a bit misleading.

In Fig 1D, etv5b expression is not reaching across the DV axis. Instead, it is expressed only in regions where the apical-basal axis of progenitors is perpendicular to the Fgf20-expressing neurons. We have improved Fig 1A to make more clear the axes in the zebrafish hindbrain.

3. In figure 1 panels D and E are switched.

Many thanks, this has been corrected.

4. In Figure 5 the authors show that transgenic overexpression of Fgf20a promotes formation of excess OPCs and a thinner hindbrain mantle zone, with apparently fewer neurons. They use these data to support the statement on p. 9, "suggesting that Fgf20 promotes OPCs at the expense of neuronal production." This seems like a reasonable idea but there are no data to support it, and we would encourage the authors to consider a more nuanced interpretation. The main idea behind the authors' model, we think, is that neurons and OPCs arise from common progenitors through some sort of binary switch. Indeed, the paper by Zannino and Appel (2009) shows that alcama+ motor neurons and OPCs arise from a common population of olig2+ precursors. Thus, one possible prediction from the authors' model is that Fgf20 inhibits alcama+ motor neuron production. However, that is not what they find. In fact, mutant embryos have fewer alcama+ motor neurons and overexpression does not block their formation. Thus, the neuron populations affected by the overexpression experiment are not alcama+ motor neurons. An alternative explanation is that Fgf20a overexpression affects neurons and OPCs independently. Sorting out these distinctions would require lineage tracing. We do not think that it is necessary to do those experiments for this paper, but it would be helpful for the authors to more fully consider this point.

This is an important point and we have modified the text to clarify our interpretation (p.13). Indeed we were not lineage tracing the effects of Fgf20 signalling on olig2+ cells that are precursors for both alcama+ motor neurons and OPCs. One potential explanation for the mild effects of Fgf20 levels on the production of alcama+ motor neurons is the opposing effects of Fgf20 signalling in: (i) promoting olig2 expression in competent progenitors, which potentially increases the number of alcama+ motor neurons, as olig2 promotes proneural gene expression (Mizuguchi et al., 2001; Novitch et al., 2001); (ii) downregulating proneural gene expression, which could decrease the number of alcama+ motor neurons. Further studies with more detailed dissection of the gene regulatory network, with consideration of the temporal window of alcama+ motor neurons specification and using lineage tracing, will be necessary to understand the effects of Fgf20 on bipotential olig2+ cells. Here, we were able to probe the regulatory effects of Fgf20 at a larger scale across the hindbrain to find that it downregulates proneural genes while upregulating gliogenic factors.

5. For experiments described in Figure 6, the authors use OPC "delamination" as an indicator of differentiation state and indicate that one of their manipulations does not result in delamination. This is a pretty weak and not very informative point. First, the images are not of sufficient magnification and resolution to assess delamination. Second, we are not convinced that it is a very meaningful indicator of differentiation or that it provides much insight to the experimental result.

We have altered the text and figures to exclude comments on delamination.

© 2024. Published by The Company of Biologists under the terms of the Creative Commons Attribution License (https://creativecommons.org/licenses/by/4.0/).

6. The legend for Figure 6 states, "Fgf20 signaling upregulates olig2 and sox10 expression in competent progenitors". In text associated with Figure 3 the authors state that "all hindbrain neural progenitors are competent to respond to fgf20". Because olig2 and sox10 upregulation are restricted, this appears to be two different uses of the term "competent". It might be helpful for the authors to clarify what they mean by progenitors competent to upregulate olig2 and sox10 in response to Fgf20.

Many thanks for pointing this out. Indeed, 'competent' has a specialised meaning in the context of cell differentiation, as well as a broader colloquial meaning. We have adjusted the text to clarify that "competent progenitors" is referring to those that are competent to become glial in response to Fgf signalling. The description in association with Figure 3 (p.7) is referring to the finding that all hindbrain progenitors are able to receive Fgf20 signalling, and we now use the terms 'ability' and 'receptive'.

7. In the first paragraph of the discussion section, the authors state that "fgf20-expressing neuronal cluster pattern progenitors spatially and temporally" and that fgf20 induces "oligodendrogenesis among competent cells in that region through upregulation of OPC specification factors, olig2 and sox9, and differentiation factor, sox10". However, the authors do not show sox9 expression data.

Although the sox9 expression data are not shown in this manuscript, our previous study by Gonzalez-Quevedo *et al*. (2010) has demonstrated that sox9 is upregulated in the hindbrain segment centre by fgf20a using BCIP/NBT in-situ hybridisation in WT and *fgf20a-/-*. We have added the reference here to clarify this point (p.11).

Reviewer 3: SUMMARY OF THE ADVANCE MADE IN THIS PAPER AND ITS POTENTIAL SIGNIFICANCE TO THE FIELD
In this manuscript, the authors study the role of Fgf20 in spatially and temporally patterning progenitor cells during Zebrafish hindbrain development. The authors find that Fgf20 is expressed by early born neurons starting at 16 hpf, and that this leads to activation of target genes Etv5b and Spry only in neighboring progenitor cells by 20 hpf. Ubiquitous overexpression of Fgf20 can result in broad expression of Etv5b, suggesting that all progenitors are capable of responding to Fgf20. They also show that at later time points (30-36 hpf) Fgf20 is required for repressing neural progenitor genes (Neurog1/2), and activating oligodendrocyte progenitor genes (Olig2/Sox10) at the segment centre. Loss of function in fgf20 knockouts leads to an increase in Nerog1/2 expression in segment center between 30-36 hpf, and a reduction of Olig2/Sox10 expression, while ubiquitous overexpression of Fgf20 has the inverse effect. All together, the authors conclude that Fgf20 signaling from early born neurons acts at short range to induce the neurogenesis-to-gliogenesis switch.

The model proposed by the authors- that newborn neurons can spatially and temporally regulate the developmental potential of progenitor cells- could potentially be an exciting advance in the field. Such a mechanism would be an elegant way to control the number of neurons and glia that are generated in different segments across a developing tissue. However, some aspects of this model, especially in regards to temporal control, are either not fully supported by data, or not well explained.

SUGGESTIONS TO AUTHORS
Detailed comments with suggested experiments for revisions are below:

1. The Results section of the manuscript is difficult to follow. A schematic that shows the relevant structures in the developing hindbrain over the relevant time span would be helpful. Including a developmental timeline of important events - when fgf20 is activated, when proneural genes are downregulated, when OPC genes are activated - would also make the results more clear/easier to follow along.

© 2024. Published by The Company of Biologists under the terms of the Creative Commons Attribution License (https://creativecommons.org/licenses/by/4.0/).

Thank you for this suggestion. We have added a summary diagram (Fig 7) to clarify the results.

2. In Figure 1, the authors arrive to the conclusion that Fgf20 works at a short range, rather than as a gradient. This is based on two pieces of evidence: i) Fgf20 target genes, Etv5b and Spry1 are activated only in progenitors right next to the Fgf20 expressing neurons in wt conditions. ii) When Fgf20 is ubiquitously overexpressed with heat shock, it can activate ubiquitous expression of Etv5b in all progenitors. Therefore, the authors conclude that while all progenitors are competent to respond to Fgf20, only the cells next to Fgf20 do so because Fgf20 is a weak signal that acts at a short range.
However, another interpretation of these results is that Etv5b and Spry1 are targets that require high Fgf20 to get activated. The Shh gradient in chick/mouse spinal cords activates some targets at high concentrations and other targets at low concentrations. What is the evidence that Fgf20 cannot activate other targets at low levels, farther away from the source?

We agree that there could be unidentified targets of Fgf signalling that are activated further away from the source, though Etv5 and Spry are thought to be activated by low level signalling in comparison with other targets such as Etv4. The key issue here is that 'short' and 'long' range are relative terms. Sources of Fgf8 and Fgf3 upregulate Etv5 expression over many cell diameters; for example acting from r4 to activate etv5b across most of the hindbrain and induce segmentation of r5 and r6. In contrast, Fgf20-expressing neurons upregulate etv5b only in hindbrain progenitor cells in their immediate vicinity. Other segment centre markers induced by Fgf20a, such as *sox9b* (Gonzalez-Quevedo et al., 2010) and *metrnla* (new Supplementary Figure 15) have striking spatial overlaps with etv5b expression. The spatial complementarity between etv5b and proneural genes was also shown (Fig. 2G) (Gonzalez-Quevedo et al., 2010); thus any lower level targets would be within the neurogenic zone and not likely to be relevant to the inhibition of neurogenesis and promotion of gliogenesis that is the topic of the current study. Together, these data suggest that Fgf20 has a short functional range for this developmental context.

3. The authors hypothesize that Fgf20 homodimerization could be contributing to its short-range activity. Could the authors perform western blots to show that Fgf20 in the hindbrain (or whole animal if amount of tissue is limiting) is present as a homodimer?

We believe that analysis of how Fgf20 acts at short-range is beyond the scope of the manuscript, and as discussed in the manuscript has been explored by others in other contexts. Furthermore, we would encounter significant technical difficulties as we have not found an available antibody for Fgf20 that works in zebrafish.

4. In Figure 3, it would be helpful to see the wildtype levels of Etv5 also quantified in A and E, because of point 6.

We have added the etv5b expression level of the controls in A and E as suggested.

5. A clarification of Etv5b function in progenitors is needed. It seems that Etv5b is expressed during neurogenesis but not during gliogenesis (expression goes down over time between 24-36 hpf in Figure 4). Yet, overexpressing Fgf20, which should suppress neurogenesis and induce gliogenesis, results in upregulation of Etv5b at all timepoints where this data is shown. It is unclear if this fits the authors' model or if this is not relevant to the model for reasons not explained.

We used etv5b as a readout of Fgf20/Fgfr activity in the hindbrain as it is an early response gene as previously described in the literature (cited in the manuscript). It provides a very good indication of the timing of activity of the signalling pathway as seen in our results (Supp Fig. 6, Fig 4E). It is beyond the scope of this study to identify the role of *etv5b* in the gene regulatory network that regulates neurogenesis/gliogenesis in the progenitors.
Nonetheless, this is not crucial for interpreting the results on the role of Fgf20 signalling in regulating neurogenesis/gliogenesis. The reviewer interprets the results as showing that Etv5b is

© 2024. Published by The Company of Biologists under the terms of the Creative Commons Attribution License (https://creativecommons.org/licenses/by/4.0/).

expressed during neurogenesis but not during gliogenesis. We observed that the initiation of proneural gene downregulation matches the timing of peak Fgf20/Fgfr activity at around 24 hpf (Supp Fig. 5, Fig 4E). The previous study by Gonzalez-Quevedo et al. (2010) has shown that the gliogenic factor *sox9b* is upregulated in the segment centre by 24 hpf. And in this study, we showed that olig2, which is crucial to oligodendrogenesis is also upregulated by Fgf20 signalling from 20 hpf onwards. Therefore, etv5b upregulated by Fgf20 is expressed at the time where downregulation of neurogenesis and initiation of gliogenesis happens.

6. In Figure 3, the authors claim that the magnitude of proneural gene downregulation is dependent on the level of Fgf20. There are two points about this that are unclear:

a. The cellular resolution is unclear because of the nature of RNA-FISH data. It is therefore difficult to interpret if the authors mean that in any given cell, higher levels of Fgf20 = higher downregulation of proneural genes in surrounding cells. Or do they mean that when Fgf20 is expressed in more cells there is overall more downregulation of proneural genes in the hindbrain. If the second, this does not necessarily mean that the level of Fgf20 is what controls downregulation. If this experiment is possible, one way to test the levels claim could be to express Fgf20 at higher levels in the newborn neurons using a targeted approach and then test whether there is earlier and greater reduction of proneural genes in the segment centre/surrounding region.

Thank you for this comment – we now realise that use of the term 'level' was misleading. We were using it to mean the overall amount of Fgf20 signalling in the hindbrain segments, rather than the amount of signal experienced by individual cells. In the experiments shown in Fig.3 we are ectopically activating Fgfr throughout the hindbrain, so the increased Fgf signalling we are referring to is predominantly due to the greater number of expressing cells. This more widespread Fgfr activation is at a sufficient level per cell to suppress neurogenesis in the sites where Fgf signalling is not active in the normal situation. We have altered the presentation of this section, and related discussion, to it testing the effect of more widespread Fgf signalling due to an increased number of cells expressing Fgf20.

b. There is also temporal variability in many of these experiments, that confound interpretations. Do the authors think that high level of Fgf20 signaling is sufficient to reduce proneuronal gene expression? Fgf20 is expressed starting at 16 hpf, but proneurnal genes are excluded from the segment center ~30-36 hpf (double FISH for Fgf20a and proneural genes over this time course could be helpful). Is this because an increase in Fgf20 signal during this time window is necessary for reaching a threshold that is sufficient to suppress pro-neural genes at 36 hpf? Or is there another time-dependent factor that primes the progenitors, so that Fgf20 can downregulate proneural genes between 30 hpf and 36 hpf? This could be tested by inducing high expression of Fgf20 before 30 hpf (maybe around 20 hpf) to see if higher levels are sufficient to downregulate proneural genes at earlier time points (such as 24 hpf). All heatshock experiments in Fig 3 are done at later timepoints, making it difficult to distinguish between the two possibilities mentioned here.

Our observations do not tally with the timing of events described by the reviewer. We observe proneural gene downregulation starting by 24 hpf (Supp Fig. 5), which matches with the timing of Fgf20 signalling read-out from expression of the early response gene *etv5b*, which peaks at 24 hpf (Fig.4E). The patterning of neurogenesis becomes more pronounced over time, presumably due to a combination of further downregulation of the proneural genes by continued Fgf20 signalling in the segment centre and further upregulation of the proneural genes in the neurogenic zone flanking the hindbrain boundaries (Fig. 3C).

7. Why is the timing of heatshock different in Fig 3A, Fig 3E, and supplemental figures 6, 8. These experiments cumulatively show i) timing of peak Fgfr/Fgf20 activity after heatshock, and ii) how this effects expression of proneural genes. Keeping the timing consistent seems important as the developmental process being studied is temporally controlled.

© 2024. Published by The Company of Biologists under the terms of the Creative Commons Attribution License (https://creativecommons.org/licenses/by/4.0/).

The experiments shown in the figures mentioned have different purposes which together led to the conclusion that Fgfr/Fgf20 activity downregulates proneural gene expression in the developmental stages that we were studying. The experiment shown in Supp Fig 6 was to evaluate the properties and efficacy of the heat-shock promoter controlled Fgf20a transgenic line *Tg(hsp:fgf20a$^{-/-}$;fgf20a)* that we have generated. It allows us to establish the protocol required (included in Materials and Methods) for prolonged overexpression of Fgf20. The experiments shown in Fig 3A and 3E are redundantly showing that Fgf signalling activity via Fgfr in hindbrain downregulates proneural gene expression. The experiments shown in Supp Fig 8, which correspond to the quantification shown in Fig 3B-D, allow us to probe how the system responds to the removal of the inhibitor.
Altogether, this helps us to establish the role of Fgfr/activity in downregulating proneural gene expression in the hindbrain.

8. How do the authors explain the difference in timing of peak Etv5b activation after heat shock induction of Fgfr (3 hours) vs. Fgf20 (1hr)? supplemental figure 6 vs. supplemental figure 8/figure 3.

This is an interesting point. We do not have an explanation, but it could relate to different properties of the constitutively activated mutant of Fgfr compared with Fgf20 e.g. in stability, expression dynamics. However, this does not impact the conclusions of the study.

9. Related to point 7: Figure 5A shows that ubiquitously inducing Fgf20a from 24-30 hpf leads to precocious expression of Sox10 at 32 hpf. Does this also lead to precocious reduction of Neurog? Does this result in reduction of neurons and increase in oligodendrocytes?

We show in Fig. 3E that ubiquitously inducing Fgf20a from 24-33 hpf leads to significant downregulation of Neurog1 expression at 36 hpf compared to the level in the control. This resulted in the reduction of neurons as indicated by the decrease in thickness of the mantle zone that is made up of neurons (Fig. 5C), as well as an increase in oligodendrocytes (Fig. 5D,E).

10. Overall, the temporal nature of Fgf20 function is not clear. Clarifying this point by inducing heatshock-Fgf20 at one early and one late timepoint, and testing multiple outcomes after each heatshock: Etv5b, Neurog, Olig2/Sox10 expression, panneuronal, oligodendrocyte markers would help clarify the authors' model.
If the author's model is that levels of Fgf20 determine when and where the neurogenic to gliogenic switch happens, then Fgf20 should be sufficient to drive this transition even at early timepoints upon overexpression. However, if overexpression of Fgf20 at early timepoints is not sufficient to drive the switch, this could suggest that the temporal control of the switch is dependent on other factors. In that case, Fgf20 is required for the switch and controls where the switch happens, but does not necessarily control when it happens.

We have shown that early Fgf20 overexpression (from 20-32 hpf) induces increased sox10+ OPCs (Fig.6A), as does a later pulse of Fgf20 overexpression from 36-42 hpf (Fig.6B). Furthermore, early overexpression of Fgf20 leads to precocious specification of OPCs, with Sox10 expression detected at 32 hpf compared with 36 hpf in wild type (Fig.5A; Fig.6A). The premature and increased generation of oligodendrocytes is evidence that Fgf20 is an instructive factor that drives the switch.

11. Minor comment: Figure 1 legend: D and E are switched.

Thank you, we have corrected this.

Second decision letter

© 2024. Published by The Company of Biologists under the terms of the Creative Commons Attribution License (https://creativecommons.org/licenses/by/4.0/).

MS ID#: DEVELOP/2024/204256

MS TITLE: Short-range Fgf signalling patterns hindbrain progenitors to induce the neurogenesis-to-oligodendrogenesis switch

AUTHORS: David G. Wilkinson; Tim Justina Yeung
ARTICLE TYPE: Research Article

Thank you for reviewing the above paper. After careful consideration, we have decided to accept this manuscript.

I am happy to tell you that your manuscript has been accepted for publication in Development, pending our standard publication integrity checks. Please see comments from reviewer 3 which suggest optional addition of 2 sentences about possible caveats of cluster annotation and explanation for editing in unexpected cells. I don't view these as critical but they may help the readers to better understand your study. If you decide to add in 1 or both sentences please let the Development office and myself know with tracked changes. Alternatively or additionally, I encourage you to publish the peer review with your paper which will allow readers to see this suggestion.

Reviewer 1

Advance summary and potential significance to field

The authors have addressed my earlier comments very well and have revised and developed the Discussion accordingly. This is a carefully conducted study that provides new insights into FGF signalling regulation of the gliogenic switch during neural differentiation and it is appropriate for publication in Development.

Reviewer 2

Advance summary and potential significance to field

I appreciate the efforts that the authors made for revision and I have no further comments on the manuscript. Congratulations to the authors for this substantial and important contribution to the field.

Reviewer 3

Advance summary and potential significance to field

This paper advances the field by presenting a mechanism by which the numbers of neurons and glia generated in developing brain regions can be regulated. Using a combination of loss of function and gain of function studies in the zebrafish hindbrain, the authors show that newborn neurons produce fgf20, which signals at short range to promote a neurogenic to gliogenic switch in progenitors. This mechanism leads to an elegant spatiotemporal regulation of the proportion of neurons and glia produced by progenitor cells in specific brain regions. As the neurogenic to gliogenic switch is an important and conserved feature of brain development, this work makes a significant contribution that is of broad interest.

Comments for the author

The authors responses and clarifications have sufficiently addressed my concerns. I do not have additional suggestions.

© 2024. Published by The Company of Biologists under the terms of the Creative Commons Attribution License (https://creativecommons.org/licenses/by/4.0/).
